# Supplementary material for: A tailored approach towards informing relatives at risk of inherited cardiac conditions: study protocol for a randomised controlled trial
Source: BMJ Open. 2019 Jul 9;9(7):e025660. doi: 10.1136/bmjopen-2018-025660 (PMC6615798; doi:10.1136/bmjopen-2018-025660)
Supplement: Supplementary data [file bmjopen-2018-025660supp001.pdf]

Supplementary material S1: Information letter and informed consent form probands and relatives

Dear Sir or Madam,

Recently you visited the department of Clinical Genetics for genetic counselling concerning a possible inherited cardiac disease in your family. A clinical geneticist or genetic counsellor informed you about participation in a research study examining how to inform relatives at risk of inheriting the genetic predisposition. In this letter, we would like to inform you about this research study.

What is the goal of this research study?
At the current moment, we ask people in whom a genetic cause for a cardiac disease is identified to inform their relatives about the possibility of genetic testing, supported by a letter written by the clinician. This research study aims to investigate whether another approach to informing relatives would be more effective. Using this approach, people who are the first in their family in whom the hereditary predisposition is identified can decide which relatives they prefer to inform themselves and which relatives they prefer to have contacted directly by the clinician. In addition, a website will be used to further inform relatives. This research study will investigate which approach is the most effective.

Who is conducting the study?
This research study will be conducted by the department of Clinical Genetics and the department of Medical Psychology of the Amsterdam UMC, in collaboration with the department of Clinical Genetics of the University Medical Centre Groningen (UMCG) and the University Medical Centre Utrecht (UMCU). The study is funded by the Dutch Heart Foundation.

What does study participation involve?
If the DNA test shows that no genetic variant causing the disease is identified, no further activity is needed for this study. If the DNA test shows that a genetic variant causing the disease is identified, the clinician will inform you about advice for your relatives. To investigate what the most effective approach is, one group of participants will be asked to inform relatives using the approach that is currently used, while the other group of participants will be asked to inform relatives using the other approach. Which group you are part of will be determined randomly.

In addition, we will ask you to fill out two questionnaires. These questionnaires can be administered online or by mail. If you prefer to receive the questionnaire by mail, the questionnaire can be returned using the provided return envelope. These questionnaires ask you for some general information, such as your age and gender, and about your experiences with informing relatives and your opinion regarding the approach that was used. You will receive the first questionnaire two months after you receive the DNA test result. The second questionnaire will be sent to you after nine months.

What are advantages and disadvantages of study participation?
There will be no direct benefit to you for your participation in this study. We hope that the information obtained from this study may contribute to research on improving approaches to informing relatives at risk of inherited cardiac diseases. Participation in this research study will take time: we will ask you to fill out two questionnaires. We expect that this will cost you 20 to 30 minutes per questionnaire. In addition, we will ask questions regarding personal information, such as the disease in your family and your opinion and feelings concerning informing your relatives about this disease. You will not receive an incentive for study participation.

What if I do not want to participate anymore?
Your participation in this research is entirely voluntary. There are no consequences if you decide not to participate. You may also change your mind later and stop participating during the study even if you earlier agreed to do so. If you decide to stop participation, please let us know. The data collected up to that moment will be used for the research study. The researcher will inform you if there is new information about the study that might be important for you. In that case, you will be asked if you still consent to participate in the study.

How will my personal data be handled?
By participating in this study, you will provide us personal data. This data will be collected for the research study. A research code will be assigned to all research documents. This means that your name and other personal data are not visible on research documents. Only the researchers know which research code belongs to you. Some institutes (the Safety Committee and the Health Care Inspectorate) are allowed to look into your clinical and personal data. These institutes are allowed to do this to control whether the research study is conducted in a proper and reliable manner. They will treat your data confidentially. If you sign the informed consent form, you will provide consent for collection, storage and inspection of your medical and personal data. Your data will be stored for 15 years.

Do you have any further questions regarding this research study?
If you have any questions about this research study or about study participation, please contact the executing researcher Lieke van den Heuvel by telephone (<telephone number>) or e-mail (<e-mail address>). If you prefer to discuss study participation with an independent clinician, or if you would like additional information, advice or support, please contact the independent expert (see attachment).

What if I want to participate?

The researcher, Lieke van den Heuvel, will contact you by telephone to provide further information about the research study. If you decide to participate in this study after this telephone contact, a consent form will be sent to you by mail. You can return this consent form by using the attached return envelope. A postage stamp is not necessary.

Thank you for your time!

Sincerely,

Lieke van den Heuvel, MSc

Also on behalf of the research team and all the clinical geneticists and genetic counsellors involved from Amsterdam UMC, University Medical Centre Utrecht and University Medical Centre Groningen

Attachment: Information about your hospital 'UMCG/Amsterdam UMC/UMCU'

Questions, suggestions or complaints
If you have any questions, suggestions or a complaint about this research study, please contact Lieke van den Heuvel. She will conduct this research study. She can be contacted by telephone (<telephone number>) or by e-mail (<e-mail address>).

Independent advice or support
If you prefer to discuss study participation with someone who is not involved in this research study, because you need further information or advice, you may contact <independent researcher> (<e-mail address>). He/she is a clinical geneticist at the department of Clinical Genetics at UMCG/Amsterdam UMC/UMCU.

Certificate of consent
For participation in a research study regarding informing relatives at risk of an inherited cardiac disease

- I am satisfied about the information I received about this research study. I have had sufficient time to consider participation in this study. I have had the opportunity to ask questions about it and any questions that I have asked have been answered to my satisfaction.
- I can stop study participation at any time, without providing any reason and without any consequences.

<u>By signing this form, I provide consent for:</u>

1. Informing my clinician (clinical geneticist/genetic counselor) about my participation in this research study.
2. Collecting of data using questionnaire(s), and inspection of my medical record until 12 months after signing this consent form and the use of these data for the research study described in this letter.
3. Being approached for other research projects in the future.
4. Storing of my research data for 15 years after the research study has been finished
5. Inspection of the research data by the Safety Committee and the Dutch Health Care Inspectorate

I want to participate in this research study

Name of participant: ____

Date: ____/____/___

Signature of participant:____

I would like to be informed about the group results of this research study:

- ☐ Yes
- ☐ No

Statement by the researcher: I have accurately informed this person about the aforementioned research project. The person may stop participating at any time during the study without any consequences. Any questions from the person about study participation were answered sufficiently

Name of researcher:____

Date: ____/____/____

Signature of researcher: ____

Supplementary Material S1

Dear Sir or Madam,

Recently you visited the department of Clinical Genetics for genetic counselling concerning an inherited cardiac disease in your family. A clinical geneticist or genetic counsellor informed you about participation in a research study examining how to inform relatives at risk of inheriting the genetic predisposition. In this letter, we would like to inform you about this research study.

What is the goal of this research study?
At the current moment, we ask people in whom a genetic cause for a cardiac disease is identified to inform their relatives about the possibility of genetic testing, supported by a letter written by the clinician. This research study aims to investigate whether another approach to informing relatives would be more effective. Using this approach, people who are the first in their family in whom the hereditary predisposition is identified can decide which relatives they prefer to inform themselves and which relatives they prefer to have contacted directly by the clinician. In addition, a website will be used to further inform relatives. This research study will investigate which approach is the most effective.

Who is conducting the study?
This research study will be conducted by the Department of Clinical Genetics and the Department of Medical Psychology of the Amsterdam UMC, in collaboration with the Departments of Clinical Genetics of the University Medical Centre Groningen (UMCG) and the University Medical Centre Utrecht (UMCU). The study is funded by the Dutch Heart Foundation.

What does study participation involve?
For this research study, we ask you to fill out a questionnaire. This questionnaire can be administered online or by mail. If you prefer to receive the questionnaire by mail, the questionnaire can be returned by using the provided return envelope. These questionnaires ask you for some general information, such as your age and gender, and about your experiences with being informed about the inherited cardiac disease in your family and your opinion regarding the approach that was used. This questionnaire will also ask you some questions about how you feel, at the current moment and in general, and how you generally cope with complex situations.

In addition, it is important for this research study to receive some information about your medical background. Because of this, we ask you to provide consent to inspect your medical record up to 12 months after completion of this research study. Important information includes, for example, the inherited cardiac disease diagnosed in your family and information

about your family history regarding cardiac diseases. This information will be handled confidentially.

Your clinician (the clinical geneticist/genetic counsellor) will be informed if you decide to participate in this study. You might be approached for other research studies in the future.

What are advantages and disadvantages of study participation?
There will be no direct benefit to you for your participation in this study. We hope that the information obtained from this study will contribute to research on improving approaches to inform relatives at risk of inherited cardiac diseases. Participation in this research study will take time: we will ask you to fill out two questionnaires. We expect that this will cost you 20 to 30 minutes per questionnaire. In addition, we will ask questions regarding personal information, such as the disease in your family and your opinion and feelings concerning informing your relatives about this disease. You will not receive an incentive for study participation.

What if I do not want to participate anymore?
Your participation in this research is entirely voluntary. There are no consequences if you decide not to participate. You may also change your mind later and stop participating during the study even if you earlier agreed to do so. If you decide to stop participation, please let us know. The data collected up to that moment will be used for the research study. The researcher will inform you if there is new information about the study that might be important for you. In that case, you will be asked if you still consent to participate in the study.

How will my personal data be handled?
By participating in this study, you will provide us personal data. This data will be collected for the research study. A research code will be assigned to all research documents. This means that your name and other personal data are not visible on research documents. Only the researchers know which research code belongs to you. Some institutes (the Safety Committee and the Health Care Inspectorate) are allowed to look into your clinical and personal data. These institutes are allowed to do this to control whether the research study is conducted in a proper and reliable manner. They will treat your data confidentially. If you sign the informed consent form, you will provide consent for collection, storage and inspection of your medical and personal data. Your data will be stored for 15 years.

Do you have any further questions regarding this research study?
If you have any questions about this research study or about study participation, please contact the executing researcher Lieke van den Heuvel by telephone (<telephone number>) or e-mail (<e-mail address>). If you prefer to discuss study participation with an independent clinician, or if you would like additional information, advice or support, please contact the independent expert (see attachment).

What if I want to participate?
The researcher, Lieke van den Heuvel, will contact you by telephone to provide further information about the research study. If you decide to participate in this study after this telephone contact, a consent form will be sent to you by mail. You can return this consent

form using the attached return envelope. A postage stamp is not necessary. Subsequently, you will receive the questionnaire online or by mail.

Thank you for your time!

Sincerely,

Lieke van den Heuvel, MSc

Also on behalf of the research team and all the clinical geneticists and genetic counsellors involved from Amsterdam UMC, University Medical Centre Utrecht and University Medical Centre Groningen

Attachment: Information about your hospital ‘UMCG/Amsterdam UMC/UMCU’

Questions, suggestions or complaints
If you have any questions, suggestions or a complaint about this research study, please contact Lieke van den Heuvel. She will conduct this research study. She can be contacted by telephone (<telephone number>) or by e-mail (<e-mail address>).

Independent advice or support
If you prefer to discuss study participation with someone who is not involved in this research study, because you need further information or advice, you may contact <independent researcher> (<e-mail address>). He/she is a clinical geneticist at the department of Clinical Genetics at UMCG/Amsterdam UMC/UMCU.

Supplementary Material S1

Certificate of consent
For participation in a research study regarding informing relatives at risk of an inherited cardiac disease

- I am satisfied about the information I received about this research study. I have had sufficient time to consider participation in this study. I have had the opportunity to ask questions about it, and any questions that I have asked have been answered to my satisfaction.
- I can stop study participation at any time, without providing any reason and without any consequences.

<u>By signing this form, I provide consent for:</u>

6. Informing my clinician (clinical geneticist/genetic counselor) about my participation in this research study.
7. Collection of data using questionnaire(s), and inspection of my medical record until 12 months after signing this consent form and the use of these data for the research study described in this letter.
8. Being approached for other research projects in the future.
9. Storage of research data for 15 years after the research study has been finished
10. Inspection of the research data by the Safety Committee and the Dutch Health Care Inspectorate

I want to participate in this research study

Name of participant: ____

Date: ____/____/___

Signature of participant:____

I would like to be informed about the group results of this research study:

- ☐ Yes
- ☐ No

Statement by the researcher: I have accurately informed this person about the aforementioned research project. The person may stop participating at any time during the study without any consequences. Any questions from the person about study participation were answered sufficiently

Name of researcher:____

Date: ____/____/____

Signature of researcher: ____
