# Supplementary material for: A tailored approach towards informing relatives at risk of inherited cardiac conditions: study protocol for a randomised controlled trial
Source: BMJ Open. 2019 Jul 9;9(7):e025660. doi: 10.1136/bmjopen-2018-025660 (PMC6615798; doi:10.1136/bmjopen-2018-025660)
Supplement: Supplementary data [file bmjopen-2018-025660supp002.pdf]

Supplementary material S2: Overview of questionnaire items to assess secondary outcomes per time-point

Table 1 Questionnaire items probands per time-point

Questionnaire time-point	Items	Questionnaires
T1	Sociodemographic, clinical, family and personality factors	Self-constructed items<br>Trait scale of STAI<br>Shortened version of TMSI
	Advices for relatives at risk, eligible relatives at risk, the number of informed relatives at risk, risk perception and experiences with informing relatives at risk	Eleven self-constructed items
	Evaluation of used approach (incl. website)	Thirteen self-constructed items
	Perceived impact on family communication with relatives at risk	Adapted version of ODCF<br>One self-constructed item
	Impact on psychological functioning of proband	HADS<br><br>Adapted version of CWS
T2	Number of informed relatives at risk, risk perception and experiences with informing relatives at risk	Three self-constructed items
	Evaluation of approach used	One self-constructed item
	Perceived impact on family communication with relatives at risk	Adapted version of ODCF<br>One self-constructed item
	Impact on psychological functioning of proband	HADS<br>Adapted version of CWS

Table 2 Questionnaire items relatives

Questionnaire time-point	Items	Questionnaires
T1	Sociodemographic, family and personality factors	Eight self-constructed items<br>Trait scale of STAI<br>Shortened version of TMSI
	Evaluation of used approach (incl. website), risk perception	Thirteen self-constructed items
	Perceived impact on family communication with index patient	Adapted version of ODCF<br>One self-constructed item
	Impact on psychological functioning of family member	HADS<br>Adapted version of CWS

Table 3 Self-constructed items (telephone interview) - Experiences with informing relatives at risk (probands)

1. Did the genetic counsellor give you an advice for your relatives?<br>2. What was this advice? (open question)<br>3. For which relatives was this advice meant? (open question)	Yes/No

4. Have relatives at risk been informed about the advice of the genetic counsellor? If yes, which relatives have been informed?<br>5. Who informed your relatives?	Yes/No

Table 4 Self-constructed items (telephone interview) - Risk perception (probands)

1. How do you consider the risk of your relatives on being a carrier of the familial variant?<br>2. How do you estimate the risk of your relatives on developing symptoms of the ICC?	0% - 100%<br>1-10<br>0% - 100%<br>1-10

Table 5 Self-constructed items - Evaluation of the used approach (probands)

T1	*Closed questions*<br>*Below you can see statements regarding your experiences with how your relatives have been informed. Please rate each statement on a scale of 1-5 (1 totally disagree to 5 totally agree) how much each statement applies to you.*	
	1. I feel supported by the genetic counsellor in informing my relatives	1 2 3 4 5
	2. I think the used approach to inform relatives at risk is acceptable	1 2 3 4 5
	3. I felt a little coerced to inform my relatives	1 2 3 4 5
	4. The way my relatives are informed, can be improved	1 2 3 4 5
	5. I am satisfied with the way my relatives are informed	1 2 3 4 5
	Other:_______________________________	
-------	---	
T2	1. Did your opinion regarding the used approach change?<br>a. Yes, my opinion regarding the used approach became more positive<br>b. Yes, my opinion regarding the used approach became more negative<br>c. No, my opinion regarding the used approach is still positive<br>d. No, my opinion regarding the used approach is still negative<br>e. No, my opinion regarding the used approach is still neutral	
-------	---	
T1/T2	1. Do you think another approach to inform relatives at risk would have been better?	Yes/No
	2. Are there relatives for which you would have preferred another approach to inform them?	Yes/No
-------	---	
T1/T2	*Open questions*<br>1. What are advantages of the approach used to inform your relatives?<br>2. What are disadvantages of the approach used to inform your relatives?	

Table 6 Self-constructed items - Impact on family relationships (probands)

1. Are there relatives with whom your relationship has changed after they are informed about their risk on the inherited cardiac disease?

a. Yes, our relationship improved<br>b. Yes, our relationship worsened<br>c. No, our relationship is still not good/not bad<br>d. No, our relationship is still good<br>e. No, our relationship is still bad

Table 7 Self-constructed items - Evaluation of the used approach (relatives)

Closed questions<br>1. How were you informed about the hereditary predisposition in your family?<br>a. With an information letter from the hospital, received from a relative<br>b. With a letter written by a relative<br>c. In person by a relative<br>d. With an information letter from the hospital, received from a genetic counsellor/clinical geneticist<br>e. In person by a genetic counsellor/clinical geneticist<br>2. What information did you receive (multiple answers are possible)?<br>a. The risk to be a carrier of the hereditary predisposition for the inherited cardiac disease in my family<br>b. The possibility to make an appointment for predictive DNA testing at an outpatient clinic Clinical Genetics<br>c. The advice to be regularly monitored by a cardiologist in the hospital<br>d. Something else, namely________________________________	
Below you can see statements regarding your experiences with how you have been informed about the inherited cardiac disease in your family. Please rate each statement on a scale of 1-5 (1 totally disagree to 5 totally agree) how much each statement applies to you.	
1. I appreciated to be informed about my risk on the inherited cardiac disease	1 2 3 4 5
2. I am satisfied with the way I have been informed	1 2 3 4 5
3. I preferred to have received more information before I contacted the clinical genetic centre	1 2 3 4 5
4. I understand why I have been informed	1 2 3 4 5
5. The way I have been informed, can be improved	1 2 3 4 5
6. I felt free to decide myself whether I wanted to contact the clinic genetic centre	1 2 3 4 5
7. I would have preferred not to be informed about my risk on the inherited cardiac disease in my family	1 2 3 4 5
8. I would have preferred to not know about the inherited cardiac disease in my family	1 2 3 4 5
Other:________________________________	
1. Do you think another approach to be informed would have been better?	Yes/No
Open questions	

1. What are advantages of the way you have been informed?<br>2. What are disadvantages of the way you have been informed?	

Table 8 Self-constructed items - Impact on family relationships (relatives)

1. Did your relationship with your relative change after they were informed about their risk on the inherited cardiac disease?<br>a. Yes, our relationship improved<br>b. Yes, our relationship worsened<br>c. No, our relationship is still not good/not bad<br>d. No, our relationship is still good<br>e. No, our relationship is still bad
