# Supplementary material for: A tailored approach towards informing relatives at risk of inherited cardiac conditions: study protocol for a randomised controlled trial
Source: BMJ Open. 2019 Jul 9;9(7):e025660. doi: 10.1136/bmjopen-2018-025660 (PMC6615798; doi:10.1136/bmjopen-2018-025660)
Supplement: Author's manuscript [file bmjopen-2018-025660.draft_revisions.pdf]

BMJ Open

BMJ Open is committed to open peer review. As part of this commitment we make the peer review history of every article we publish publicly available.

When an article is published we post the peer reviewers' comments and the authors' responses online. We also post the versions of the paper that were used during peer review. These are the versions that the peer review comments apply to.

The versions of the paper that follow are the versions that were submitted during the peer review process. They are not the versions of record or the final published versions. They should not be cited or distributed as the published version of this manuscript.

BMJ Open is an open access journal and the full, final, typeset and author-corrected version of record of the manuscript is available on our site with no access controls, subscription charges or pay-per-view fees (http://bmjopen.bmj.com).

If you have any questions on BMJ Open's open peer review process please email info.bmjopen@bmj.com

BMJ Open

A tailored approach towards informing relatives at risk of inherited cardiac conditions: study protocol for a randomized controlled trial

Journal:	*BMJ Open*
Manuscript ID	bmjopen-2018-025660
Article Type:	Protocol
Date Submitted by the Author:	27-Jul-2018

Complete List of Authors:	van den Heuvel, Lieke; Amsterdam University Medical Centres, Department of Clinical Genetics<br>Hoedemaekers, Yvonne; University Medical Centre Groningen, Department of Clinical Genetics<br>Baas, Annette; University Medical Center Utrecht, Department of Genetics<br>van Tintelen, J; Amsterdam University Medical Centres, Department of Clinical Genetics<br>Smets, Ellen; Amsterdam University Medical Centres, Department of Medical Psychology<br>Christiaans, Imke; Amsterdam University Medical Centres, Clinical Genetics

Keywords:	Inherited cardiac conditions, Cardiogenetics, Informing relatives at risk, Family-mediated approach, Tailored approach, Randomized controlled trial

SCHOLARONE™
Manuscripts

For peer review only

A tailored approach towards informing relatives at risk of inherited cardiac conditions: study protocol for a randomized controlled trial

L.M. van den Heuvel[1], Y.M. Hoedemaekers[2], A.F. Baas[3], J.P. van Tintelen[1], E.M.A. Smets[4] & I. Christiaans[1*]

[1]Department of Clinical Genetics, Amsterdam University Medical Centres / University of Amsterdam, Amsterdam, the Netherlands;

[2]Department of Clinical Genetics, University Medical Centre Groningen / University of Groningen, Groningen, the Netherlands;

[3]Department of Genetics, University Medical Centre Utrecht / University Utrecht, Utrecht, the Netherlands;

[4]Department of Medical Psychology, Amsterdam University Medical Centres / University of Amsterdam, Amsterdam, the Netherlands.

* Corresponding author

Department of Clinical Genetics,

Amsterdam University Medical Centre, location AMC

Meibergdreef 9, 1105 AZ, Amsterdam, The Netherlands

Telephone: +31 20-5667217

E-mail: i.christiaans@amc.nl

For peer review only

For peer review only - http://bmjopen.bmj.com/site/about/guidelines.xhtml

Abstract

Introduction In current practice, probands are asked to inform relatives about the possibility of predictive DNA testing if a pathogenic variant causing an inherited cardiac condition (ICC) is identified. Previous research on uptake of genetic counselling and predictive DNA testing in relatives suggests that not all relatives are sufficiently informed. We developed a randomized controlled trial to evaluate the effectiveness of a tailored approach, in which probands, together with the genetic counsellor, decide which relatives they inform themselves and which relatives they prefer to be informed by the genetic counsellor. Here, we present the study protocol of this randomized controlled trial. **Methods** A multicentre randomized controlled trial with parallel-group design will be conducted, in which a tailored approach (i.e., intervention group) will be compared to a control group of usual care. Adult probands diagnosed with an ICC in whom a likely pathogenic or pathogenic variant is identified, will be randomly assigned to the intervention or control group (total sample n = 85 probands). The primary outcome is uptake of genetic counselling and predictive DNA testing in relatives (total sample n = 340 relatives). Secondary outcomes are appreciation of the used approach and impact on family- and psychological functioning, which will be assessed using questionnaires. Relatives of included probands who attend genetic counselling will be asked to fill out a questionnaire as well. **Ethics and dissemination** Ethical approval was obtained from the Medical Ethical Committee of the Amsterdam University Medical Centres (MEC 2017-145), the Netherlands. All participants provide informed consent prior to participation in this study. Results of the study on primary and secondary outcome measures will be published in peer-reviewed journals. **Registration details** This trial is registered at the Netherlands Trial Register NTR6657.

Key words Inherited cardiac conditions, cardiogenetics, informing relatives at risk, family-mediated approach, tailored approach, randomized controlled trial

For peer review only - http://bmjopen.bmj.com/site/about/guidelines.xhtml

Strengths and limitations of this study

Strength: To our knowledge, this is the first randomized controlled trial to evaluate a tailored intervention towards informing relatives at risk, compared to a control group of standard care.

Strength: This randomized controlled trial investigates the uptake of genetic counselling and predictive DNA testing, as well as the acceptance and impact on psychological and family functioning of the tailored versus the standard approach.

Strength: Both probands and relatives will be included in this study to assess their experiences with and their attitudes towards the used approach .

Limitation: In this randomized controlled trial, it is not possible to blind participants or genetic counsellors for the chosen intervention. Neither the executing investigator can be blinded for randomization.

Limitation: Only relatives of probands included in the study who attend genetic counselling can be approached for participation in the study.

For peer review only

Introduction

Inherited cardiac conditions (ICCs), such as cardiomyopathies and primary arrhythmia syndromes, generally demonstrate an autosomal dominant inheritance pattern, and a wide variety of symptoms can manifest at any age (1, 2). A feared outcome is sudden cardiac death (SCD), often at young age, which can be the first symptom of disease (3, 4). With an incomplete penetrance and high variability in expression, even within families, carriers of the familial variant may remain undetected but can still be at risk for SCD, while treatment options are available to prevent disease progression or potential life-threatening arrhythmias (5).

Predictive DNA testing is therefore offered to first-degree relatives of probands (i.e., the first person in a family diagnosed with an ICC) in whom a pathogenic variant is identified, who are at 50% risk of inheriting the genetic variant (5, 6). Predictive DNA testing is offered in a stepwise manner (i.e., cascade screening), with the aim to identify asymptomatic carriers of the familial variant to facilitate timely treatment. Non-carriers of the familial variant generally do not need cardiac monitoring and can be reassured about their own risk and that of their offspring (6).

In current practice in the Netherlands, probands are asked to inform their relatives, supported by a family letter written by the genetic counsellor. This is referred to as the family-mediated approach (7). Previous research, however, shows that uptake (i.e., the number of relatives at risk attending genetic counselling and/or predictive DNA testing) is relatively low in ICCs, especially in cardiomyopathies. Reported uptakes are less than 50%, despite family letters being provided to a majority of relatives by the proband (8-10). Previous research in other genetic patient populations, such as hereditary types of cancer, shows similar percentages (11-13).

For peer review only - http://bmjopen.bmj.com/site/about/guidelines.xhtml

Some relatives who do not attend genetic counselling will have deliberately decided against predictive DNA testing. However, these low uptake percentages also suggest that part of the relatives might be unaware or insufficiently aware of the risks involved and/or the possibilities for genetic counselling and subsequent surveillance and treatment. This is supported by research on family communication in ICCs. Patients are not always able to inform or correctly inform their relatives because of several reasons, including disengagement with relatives, lack of understanding of the importance of the information, preoccupation with their own grief, difficulties in conveying the complex information to relatives, or the wish to prevent burdening on relatives by informing them about genetic risks (8, 14-18).

A few studies have been published on more active approaches towards informing relatives at risk, in which HCPs directly contact at-risk relatives (19-22). These studies suggest that a more active approach can nearly double the uptake of genetic counselling and predictive DNA testing of relatives (19-22). However, these studies were performed in a research setting (e.g., in relatives already registered in research databases for the genetic disease), hampering direct translation of these results to a diagnostic setting. To our knowledge, more active approaches in patients with ICCs have not been studied so far. However, a study of Ormondroyd et al suggests that relatives eligible for predictive DNA testing for hypertrophic cardiomyopathy and long QT syndrome would support a more active approach to inform relatives at risk (15).

Although studies on more active approaches did not report any psychological harm in relatives on group level, these approaches could cause more unwarranted worries or pressure on relatives to opt for predictive DNA testing (19-21). An active approach towards informing relatives at risk could also breach the autonomy and confidentiality of probands, and may harm the right not to know of relatives (23-25). Furthermore, healthcare professionals (HCPs) are often unaware of interpersonal dynamics within families and personal circumstances of

For peer review only - http://bmjopen.bmj.com/site/about/guidelines.xhtml

relatives at risk. Active approaches may therefore have a negative impact on family relationships or may cause psychological distress in both probands and relatives (26).

Because of this, a tailored approach in which probands may decide together with the genetic counsellor which at-risk relatives they will inform themselves and which relatives they prefer to be informed by the genetic counsellor, could be optimal. With this approach, the probands' expert knowledge of relatives' functioning and family dynamics could be used appropriately and the autonomy of the proband would be preserved. At the same time more relatives at risk would be sufficiently informed (24, 26). Furthermore, probands for whom informing relatives would be difficult or burdensome might be relieved or supported by this approach (26).

Objectives

The primary aim of this randomized controlled trial is to assess whether uptake of genetic counselling and testing of relatives at risk of an ICC will be increased by using a tailored approach of information provision for relatives, instead of usual care (i.e., family-mediated approach). Secondary objectives are to evaluate how such a tailored approach is appreciated by both probands and relatives, compared to usual care. In addition, this study aims to assess the perceived impact on family relationships and psychological functioning of both probands and relatives. The protocol presented here has been described on the basis of the SPIRIT statement (27).

Methods

Design

A multicentre randomized controlled trial with a parallel-group design will be conducted in three university hospitals in the Netherlands (i.e., the Academic Medical Centre (AMC), the

For peer review only - http://bmjopen.bmj.com/site/about/guidelines.xhtml

University Medical Centre Utrecht (UMCU) and the University Medical Centre Groningen (UMCG)), comparing the effects of a tailored approach towards informing relatives at risk of ICCs to usual care in both probands and relatives.

Participants

Probands aged 18 years or older with an ICC or suspicion thereof, attending pre-test genetic counselling at the cardiogenetics outpatient clinics will be asked to participate if they: (1) are the first of their family to visit the cardiogenetic outpatient clinic for counselling about genetic testing for ICCs; (2) they have at least one alive adult relative; and (3) are able to read and write Dutch. Only probands in whom a likely pathogenic or pathogenic variant (i.e., class 4 - likely pathogenic - or class 5 - pathogenic variant) is detected will be definitively included.

In addition, eligible adult first- (or second-) degree relatives of enrolled probands who make an appointment at the cardiogenetic outpatient clinics will be invited to fill out a questionnaire to measure secondary outcomes. Inclusion criteria are defined as follows: (1) First-degree adult (i.e., 18 years or older) relatives, and second-degree adult relatives in case of a deceased connecting first-degree relative who was affected or suspected to be affected, of probands enrolled in the study; and (2) able to read and write Dutch.

Procedure

Figure 1 shows a flow-chart of the study procedure.

Recruitment & consent

During pre-test genetic counselling, the genetic counsellor will inform the probands about the study and will hand over an information letter. In addition, probands will be asked if the executing researcher can contact them to provide further information about the study.

For peer review only

For peer review only - http://bmjopen.bmj.com/site/about/guidelines.xhtml

Subsequently, probands will be contacted by telephone by the executing researcher. If probands are still interested in participation, written informed consent forms will be sent by post mail, including a return envelope. As described above, only probands in whom a likely pathogenic or pathogenic variant is detected, will be definitively included in the study.

Relatives at risk of enrolled probands attending pre-test genetic counselling in one of the participating centres, will be invited to participate in the study as well. The same recruitment procedure will be used.

Randomization

Probands with an ICC in whom a likely pathogenic or pathogenic variant is identified, will be randomly assigned to either the intervention or the control group prior to receiving their test result. Block randomization will be used, with variable blocks ranging from size two to six. Randomization will be stratified for gender, disease type (i.e., cardiomyopathies or primary arrhythmia syndromes) and hospital. To ensure allocation concealment, computer software will be used for randomization, with an allocation rate of 1:1 (28). Relatives of probands included in the study will be assigned to the group to which the proband was assigned.

Neither participants nor the genetic counsellors will and can be blinded for group assignment. The executing researcher cannot be blinded either, because of slight differences between questionnaires administered in the intervention- and control group. Part of the outcome data will be collected using telephone interviews. To minimize bias, these interviews will be conducted by a research assistant following a structured script.

Intervention group

In the intervention group, a tailored approach towards informing relatives at risk, will be provided. In this approach, probands with a likely pathogenic or pathogenic variant will

For peer review only
For peer review only - http://bmjopen.bmj.com/site/about/guidelines.xhtml

discuss with the genetic counsellor which relatives are at risk of inheriting the familial variant and are subsequently asked which of these relatives they prefer to inform themselves at first using a family letter written by the genetic counsellor, and which relatives they prefer to be directly informed by the genetic counsellor with a similar family letter.

In both cases, after one month the family letter will be sent directly by the genetic counsellor to all relatives at risk, for whom the proband has provided consent to contact them. The proband will be asked to provide contact details of these relatives.

The family letter is standardised for all three participating centres. For the intervention group, the letter also includes a link to a website specifically designed for this study where relatives can find additional information (www.familieleden.erfelijkehartziekten.nl). The information on this website is tailored to the relatives' situation (i.e., specified for disease type, hospital, whether they have a child wish and/or children, and which information they prefer to receive) by asking relatives at their first visit to the website to fill out a short questionnaire.

Control group

In the control group, the standard care approach will be used. If a likely pathogenic or pathogenic variant is identified, probands assigned to the control group will be asked by the genetic counsellor to inform relatives at risk about the genetic test result, the consequences of this result for relatives and the advice regarding predictive DNA testing and/or cardiac monitoring. Probands will be supported in informing relatives at risk by a family letter written by the genetic counsellor. This family letter is also standardised for all three participating centres. However, this letter does not include the link to the specific website for tailored information, but includes a link to a general website on ICCs (www.erfelijkehartziekten.nl).

For peer review only - http://bmjopen.bmj.com/site/about/guidelines.xhtml

Measurement time-points

For secondary outcome measures, participating probands will be asked to complete a questionnaire one month after receiving the genetic test result (T1) and a questionnaire after nine months after the test result (T2). Before T1 and T2, a short structured telephone interview will be conducted about participants' knowledge of which relatives are at risk of ICCs and which relatives are informed, because these items are expected to be too complex to answer in a questionnaire (28). Participating relatives will complete one questionnaire after attending genetic counselling.

Measures

Primary outcome measure

To assess the effect of a tailored approach towards informing relatives at risk, the difference between the intervention- and control group in uptake of genetic counselling and predictive DNA testing of relatives at risk will be measured. To do so, the number of relatives attending genetic counselling as well as the number of relatives that is genetically tested in the first year after detection of the likely pathogenic or pathogenic variant in the proband, will be collected in the laboratories of each participating centre. DNA test results of relatives counselled in non-participating centres are also taken into account, because in the Netherlands predictive DNA testing of relatives is always performed in the laboratory where the proband was tested.

The numbers of relatives attending genetic counselling and predictive DNA testing will be compared to the total number of relatives at risk of inheriting the variant eligible for genetic counselling and predictive DNA testing based on family pedigrees. For relatives who attended genetic counselling, but decided against predictive DNA testing, subsequent attendance of cardiac monitoring will be checked.

For peer review only - http://bmjopen.bmj.com/site/about/guidelines.xhtml

Relatives at risk eligible for genetic counselling and predictive DNA testing are first-degree relatives, and second-degree relatives in case of a connecting deceased first-degree relative suspected of an ICC. For cardiomyopathies, relatives at risk are eligible for genetic counselling and predictive DNA testing from the age of 10 years and over, following Dutch clinical guidelines. For primary arrhythmias, depending on the specific arrhythmic disorder, relatives at risk are eligible for predictive DNA testing from birth.

Furthermore, conditional uptake of relatives at risk, defined as the number of relatives that is genetically tested relative to the number that attends genetic counselling, will be calculated. Uptake will be measured at randomisation condition (i.e., intervention- or control group) and family level.

Secondary outcome measures

Secondary outcome measures will be conducted by using validated as well as self-constructed questionnaire items. An overview of these items is shown in the Supplementary Material. Secondary outcome measures include the following:

- Appreciation of the used information provision strategy and preferences regarding the approach used to inform relatives at risk: This will be evaluated in both probands and relatives by using self-constructed items on a 5 point Likert scale (i.e., 1 = 'Totally disagree' to 5 = 'Totally agree') in a questionnaire (probands 5 items, range 5-25; relatives 6 items, range 6-30). Probands will be asked to answer an additional self-constructed item during the structured telephone interview on whether they preferred to inform their relatives differently. In the intervention group, two additional self-constructed items will be administered to assess decisional conflict in probands, including whether probands thought it was hard to choose whether they wanted to inform their relatives themselves or by the counsellor, and whether they were satisfied by their decision on a 5

For peer review only - http://bmjopen.bmj.com/site/about/guidelines.xhtml

point Likert scale (i.e., 1 = ‘Totally disagree’ to 5 = ‘Totally agree’; range 2-10). Probands will be asked to fill out these items at T1. At T2, a self-constructed item will be administered whether their opinion regarding the used approach has changed. Finally, probands (i.e., T1 and T2) and relatives will be asked whether they visited the website www.erfelijkehartziekten.nl and if yes, how they evaluated this website, using four self-constructed items on a 5 point Likert scale (i.e., 1 = ‘Totally disagree’ to 5 = ‘Totally agree’; range 4-20).

- Impact on family communication: To assess the impact on family functioning of the tailored approach versus the usual care approach, probands (i.e., on T1 and T2) and relatives will be asked to fill out an adapted version of the Openness to Discuss Cancer in the Family (ODCF) scale, assessing communication about genetic risks within families with nine items on a 5 point Likert scale (i.e., 1 = ‘Totally disagree’ to 5 = ‘Totally agree’; range 9-45) (29). Psychometric characteristics of the original ODCF scale are satisfactory (29). In addition, a self-constructed item will be administered asking whether probands and relatives experienced changes in their relationships with relatives as a consequence of the information process.
- Impact on psychological functioning: To assess the impact on psychological functioning, two validated questionnaires will be administered in both probands (i.e., T1 and T2) and relatives. Participants will be asked to fill out an adapted version of the Cancer Worry Scale (CWS) (30). The CWS was developed and previously used in studies with patients with hereditary types of cancer. It consists of eight items on a 4 point Likert scale (i.e., 1= ‘Almost never’ to 4 = ‘Almost always’; range 8-32). Psychometric characteristics were assessed in a sample of breast cancer survivors, which supported the reliability and validity of the CWS (30).

For peer review only - http://bmjopen.bmj.com/site/about/guidelines.xhtml

In addition, the Hospital Anxiety and Depression Scale (HADS) will be administered to assess whether participants experience anxious or depressed feelings after being informed about the hereditary disease (31). The HADS contains two 7-item subscales on a 4 point Likert scale with diverse answer options, assessing anxiety and depression both with a score range of 0-21. Psychometric characteristics were assessed as good (31).

Participants' characteristics

To assess whether randomization succeeded and whether characteristics of participating probands and relatives influence primary and secondary outcome measures, sociodemographic and clinical factors will be collected, including gender, education level, ethnicity, living situation and parenthood, family history and diagnosis of the probands at T1. Relatives will be asked additionally what their degree of kinship is with the proband.

For the same reason, psychosocial and personality factors will be assessed in both probands (i.e., at T1) and relatives as well. Coping style will be assessed by using the shortened version of the Threatening Medical Situations Inventory (TMSI) (32, 33). This questionnaire assesses a monitoring and a blunting coping style related to a medical threat and is previously evaluated in an oncogenetic patient population (32, 34). The shortened version of the TMSI contains two subscales, both consisting of six items on a 5 point Likert scale (i.e., 1 = 'Totally not applicable' to 5 = 'Totally applicable'; range 6-30). Reliability and validity are satisfactory (32, 33).

The Trait subscale of the State Trait Anxiety Inventory (STAI) will be administered to assess trait anxiety in both probands and relatives. The STAI is a frequently used questionnaire in research settings, and consists of 20 items on a 4 point Likert scale (i.e., 1 =

For peer review only - http://bmjopen.bmj.com/site/about/guidelines.xhtml

For peer review only

'Not at all' to 4 = 'Very much so'; range 20-80) (35). The reliability and validity for the Dutch translation of the STAI are assessed as good (36).

Self-efficacy, and perceived motivators and barriers regarding informing relatives at risk will be assessed by using an adapted version of the 'motivation' and 'self-efficacy' subscales of the Informing Relatives Inventory (IRI) (37). The IRI was developed and evaluated in an oncogenetic patient population, showing satisfactory reliability and validity (37). The 'motivation' subscale consists of 30 items on a 5 point Likert scale (i.e., 'No role' to 'A large role'; range 30-150); the 'self-efficacy' subscale of 7 items on a 4 point Likert scale (i.e., 1= 'Not sure at all' to 4 = 'Very sure'; range 7-21).

Risk perception regarding the risk of relatives carrying the variant and developing the disease will be assessed by using self-constructed items. These items ask participants to rate the perception of the risk of relatives on carrying the variant and on developing the disease, on a scale from 1 (lowest risk) to 10 (highest risk) as well as from 0% (lowest risk) to 100% (highest risk).

Health literacy, defined as the ability to obtain, process and understand basic health information and services, will be assessed in probands and relatives, using the items on the 'functional health literacy' and 'communicative health literacy' subscales of the 3HL questionnaire (38). Both subscales contain five items on a 4 point Likert scale (i.e., 1 = 'Never' to 4 = 'Often'; range per subscale 5-20). The reliability for both scales was assessed as high and the validity as satisfactory (38).

Sample size calculation

The study aims to detect a difference of 15% in uptake of genetic counselling of relatives between the control (i.e., usual care, 50%) and the intervention group (i.e., tailored approach, 65%). Assuming a two-sided 5% significance level and a power of 80%, a number of 340

For peer review only

For peer review only - http://bmjopen.bmj.com/site/about/guidelines.xhtml

relatives (170 in each group) would be required to participate in this study. On average, a number of six relatives per proband is at 50% risk of inheriting the variant, including children and adults (9). With a conservative estimate of four eligible adult relatives per proband at risk, 85 probands with an ICC and an identified likely pathogenic or pathogenic (i.e., class 4 or 5) variant will be required to include in this study to reach 340 relatives. In on average 20% (lower margin) of all probands with a suspected ICC a likely pathogenic or pathogenic variant is found. With an expected response rate of 70% and a drop-out rate of 20%, approximately 759 probands will be approached to participate in the study.

Data analysis

Sociodemographic, clinical, psychosocial and personality variables will be analysed using descriptive and frequency statistics. An intention-to-treat approach will be used. SPSS version 24.0 will be used to perform statistical analyses (39). An α level of $p < .05$ will be used.

Student t-tests and chi-square tests will be used to assess differences (i) on sociodemographic, clinical, psychosocial and personality characteristics between the intervention- and control group, and (ii) participants and non-participants. Descriptive and frequency statistics will be used to describe the primary outcome: uptake of genetic counselling and of predictive DNA testing. Student t-tests and non-parametric statistics will be used to assess differences between the intervention- and control group on the primary outcome, as appropriate. Appreciation of the used approach will be described by using frequency statistics.

Multilevel analyses will be performed to assess whether the intervention has an impact on family and psychological functioning. The two measurement time-points will be treated as nested within probands. Regression analyses will be conducted as well to assess the influence

For peer review only - http://bmjopen.bmj.com/site/about/guidelines.xhtml

on the primary and secondary outcomes of sociodemographic, clinical, psychological and personality characteristics. Open questions will be analysed using thematic analysis.

Patient and public involvement

Prior to this randomized controlled trial, face-to-face interviews were conducted with probands and counselled relatives (both carriers and non-carriers) to explore their experiences with and preferences regarding informing at-risk relatives (unpublished). In addition, online focus groups with HCPs were conducted. Based on the findings of both these interviews and focus groups, this randomized controlled trial was designed. Since this study is part of the eDETECT research consortium (CVON2015-12), several patient representative groups (i.e., PLN foundation; Harteraad, Heartz) participate in the user committee and scientific meetings and thereby gave input to this research proposal. Patients are not involved in the recruitment to and conduct of the study.

During patient seminars, patients will be updated on the progression and results of this study. In addition, during the eDETECT scientific meetings, all participants of the eDETECT consortium (including representatives of the aforementioned patient organisations) will be informed. After completion of the study, group results will be disseminated by e-mail to study participants who indicated during informed consent to be interested. Furthermore, a summary of the results will be posted on the website on inherited cardiac conditions (www.erfelijkehartziekten.nl).

The burden of the intervention was not assessed because this is an intrinsic part of the outcome measures of this study. The patients themselves were involved in pilot testing of the questionnaires used to assess these outcome measures.

For peer review only - http://bmjopen.bmj.com/site/about/guidelines.xhtml

Ethics and dissemination

Approval of the study design has been obtained by the Medical Ethical Committee of the AMC (MEC 2017-145). Subsequently, additional approval of regional Medical Ethical Committees of the other participating academic centres has been obtained. Informed consent is required from each participant. Participants who provided written informed consent can withdraw from the study at any time, without providing a reason.

After receiving informed consent, a unique research ID will be assigned to the participant. Only this ID will be used to identify research documents. Each research document will be saved on a secured server. The principal investigator, coordinating investigator and executing investigator have access to this secured server. Research documents will be saved for a period of 15 years. This randomized controlled trial is registered at the Netherlands Trial Register NTR6657. Separate manuscripts with findings on respectively the primary and secondary outcomes will be published in peer-reviewed journals.

Trial status

Recruitment of probands during pre-test genetic counselling for this randomized controlled trial started in November 2017. In total, recruitment of probands will last one year. Subsequently, uptake of genetic counselling and predictive DNA testing will be measured until one year after the detection of a pathogenic variant in the proband. Therefore, data collection will continue until January 2020 taking into account a duration of on average three months for the DNA-test result in the proband to be available.

References

For peer review only - http://bmjopen.bmj.com/site/about/guidelines.xhtml

1. Theilade J, Kanters J, Henriksen FL, Gilsa-Hansen M, Svendsen JH, Eschen O, et al. Cascade screening in families with inherited cardiac diseases driven by cardiologists: feasibility and nationwide outcome in long QT syndrome. Cardiology. 2013;126(2):131-7.

2. Watkins H, Ashrafian H, Redwood C. Inherited cardiomyopathies. N Engl J Med. 2011;364(17):1643-56.

3. Behr ER, Dalageorgou C, Christiansen M, Syrris P, Hughes S, Tome Esteban MT, et al. Sudden arrhythmic death syndrome: familial evaluation identifies inheritable heart disease in the majority of families. Eur Heart J. 2008; 29 (13): 1670-80.

4. Van der Werf C, Hofman N, Tan HL, van Dessel PF, Alders M, van der Wal AC, et al. Diagnostic yield in sudden unexplained death and aborted cardiac arrest in the young: the experience of a tertiary referral center in The Netherlands. Heart Rhythm. 2010; 7(10):1383-9.

5. Wilde AA, Behr ER. Genetic testing for inherited cardiac disease. Nat Rev Cardiol. 2013; 10 (10): 571-83.

6. Miller EM, Wang Y, Ware SM. Uptake of cardiac screening and genetic testing among hypertrophic and dilated cardiomyopathy families. J Genet Couns. 2013; 22 (2): 258-67.

7. Leenen CH, Heijer M, van der Meer C, Kuipers EJ, van Leerdam ME, Wagner A. Genetic testing for Lynch syndrome: family communication and motivation. Fam Cancer. 2016; 15 (1): 63-73.

8. Burns C, McGaughran J, Davis A, Semsarian C, Ingles J. Factors influencing uptake of familial long QT syndrome genetic testing. Am J Med Genet A. 2016; 170A(2): 418-25.

9. Christiaans I, Birnie E, Bonsel GJ, Wilde AA, van Langen IM. Uptake of genetic counselling and predictive DNA testing in hypertrophic cardiomyopathy. Eur J Hum Genet. 2008; 16 (10): 1201-7.

For peer review only

10. Van der Roest WP, Pennings JM, Bakker M, van den Berg MP, van Tintelen JP. Family letters are an effective way to inform relatives about inherited cardiac disease. Am J Med Genet A. 2009; 149A(3): 357-63.

11. Brooks L, Lennard F, Shenton A, Lalloo F, Ambus I, Ardern-Jones A, et al. BRCA1/2 predictive testing: a study of uptake in two centres. Eur J Hum Genet. 2004; 12(8): 654-62.

12. Menko FH, Ter Stege JA, van der Kolk LE, Jeanson KN, Schats W, Moha DA, et al. The uptake of presymptomatic genetic testing in hereditary breast-ovarian cancer and Lynch syndrome: a systematic review of the literature and implications for clinical practice. Fam Cancer. 2018. DOI: 10.1007/s10689-018-0089-z.

13. Ramsoekh D, van Leerdam ME, Tops CM, Dooijes D, Steyerberg EW, Kuipers EJ, et al. The use of genetic testing in hereditary colorectal cancer syndromes: genetic testing in HNPCC, (A)FAP and MAP. Clin Genet. 2007; 72 (6): 562-7.

14. Batte B, Sheldon JP, Arscott P, Huismann DJ, Salberg L, Day SM, et al. Family communication in a population at risk for hypertrophic cardiomyopathy. J Genet Couns. 2015; 24 (2): 336-48.

15. Ormondroyd E, Oates S, Parker M, Blair E, Watkins H. Pre-symptomatic genetic testing for inherited cardiac conditions: a qualitative exploration of psychosocial and ethical implications. Eur J Hum Genet. 2014; 22 (1): 88-93.

16. Smart A. Impediments to DNA testing and cascade screening for hypertrophic cardiomyopathy and Long QT syndrome: a qualitative study of patient experiences. J Genet Couns. 2010; 19 (6): 630-9.

17. Vavolizza RD, Kalia I, Erskine Aaron K, Silverstein LB, Barlevy D, Wasserman D, et al. Disclosing Genetic Information to Family Members About Inherited Cardiac Arrhythmias: An Obligation or a Choice? J Genet Couns. 2015; 24 (4): 608-15.

For peer review only - http://bmjopen.bmj.com/site/about/guidelines.xhtml

18. Whyte S, Green A, McAllister M, Shipman H. Family Communication in Inherited Cardiovascular Conditions in Ireland. J Genet Couns. 2016; 25 (6): 1317-26.

19. Aktan-Collan K, Haukkala A, Pylvanainen K, Jarvinen HJ, Aaltonen LA, Peltomaki P, et al. Direct contact in inviting high-risk members of hereditary colon cancer families to genetic counselling and DNA testing. J Med Genet. 2007; 44 (11): 732-8.

20. Sermijn E, Delesie L, Deschepper E, Pauwels I, Bonduelle M, Teugels E, et al. The impact of an interventional counselling procedure in families with a BRCA1/2 gene mutation: efficacy and safety. Fam Cancer. 2016; 15 (2): 155-62.

21. Suthers GK, Armstrong J, McCormack J, Trott D. Letting the family know: balancing ethics and effectiveness when notifying relatives about genetic testing for a familial disorder. J Med Genet. 2006; 43 (8): 665-70.

22. Umans-Eckenhausen MA, Defesche JC, Sijbrands EJ, Scheerder RL, Kastelein JJ. Review of first 5 years of screening for familial hypercholesterolaemia in the Netherlands. Lancet. 2001; 357 (9251): 165-8.

23. Mitchell C, Ploem MC, Hennekam RC, Kaye J. A Duty To Warn Relatives in Clinical Genetics: Arguably 'Fair just and reasonable' in English Law? Tottels J Prof Neglig. 2016; 32 (2): 120-36.

24. Weaver M. The Double Helix: Applying an Ethic of Care to the Duty to Warn Genetic Relatives of Genetic Information. Bioethics. 2016; 30 (3): 181-7.

25. Wouters RH, Bijlsma RM, Ausems MG, van Delden JJ, Voest EE, Bredenoord AL. Am I My Family's Keeper? Disclosure Dilemmas in Next-Generation Sequencing. Hum Mutat. 2016; 37 (12): 1257-62.

26. Leonard SJ, Newson, A. J. Ethical perspectives. In: Gaff CL, Bylund, C.L. Family communication about genetics: Theory and practice. New York: Oxford University Press, inc.; 2010.

For peer review only

For peer review only - http://bmjopen.bmj.com/site/about/guidelines.xhtml

27. Chan AW, Tetzlaff JM, Altman DG, Laupacis A, Gotzsche PC, Krleza-Jeric K, et al. SPIRIT 2013 statement: defining standard protocol items for clinical trials. Ann Intern Med. 2013;158(3):200-7.

28. BV Castor. Castor Electronic Data Capture. Amsterdam; 2016.

29. Mesters I, van den Borne H, McCormick L, Pruyn J, de Boer M, Imbos T. Openness to discuss cancer in the nuclear family: scale, development, and validation. Psychosom Med. 1997; 59 (3): 269-79.

30. Custers JA, van den Berg SW, van Laarhoven HW, Bleiker EM, Gielissen MF, Prins JB. The Cancer Worry Scale: detecting fear of recurrence in breast cancer survivors. Cancer Nurs. 2014; 37 (1): E44-50.

31. Spinhoven P, Ormel J, Sloekers PP, Kempen GI, Speckens AE, Van Hemert AM. A validation study of the Hospital Anxiety and Depression Scale (HADS) in different groups of Dutch subjects. Psychol Med. 1997; 27 (2): 363-70.

32. Ong LM, Visser MR, van Zuuren FJ, Rietbroek RC, Lammes FB, de Haes JC. Cancer patients' coping styles and doctor-patient communication. Psychooncology. 1999; 8 (2):155-66.

33. Van Zuuren FJ, De Groot, K.I., Mulder, N.L., Muris, P. Coping with medical threat: An evaluation of the Threatening Medical Situations Inventory (TMSI). Personality and Individual Differences. 1996; 21 (1): 21-31.

34. Wakefield CE, Homewood J, Mahmut M, Taylor A, Meiser B. Usefulness of the Threatening Medical Situations Inventory in individuals considering genetic testing for cancer risk. Patient Educ Couns. 2007; 69 (1-3): 29-38.

35. Spielberger CD. Assessment of state and trait anxiety: Conceptual and methodological issues. South Psychol. 1985; 2: 6-16.

For peer review only

For peer review only - http://bmjopen.bmj.com/site/about/guidelines.xhtml

36. Ploeg H.M van der DPB, & Spielberger, C.D. Handleiding bij de Zelf-Beoordelings Vragenlijst ZBV. Een Nederlandse bewerking van de Spielberger State-Trait Anxiety Inventory STAI-DY. Lisse: Swets & Zeitlinger; 1981.

37. De Geus E, Aalfs CM, Menko FH, Sijmons RH, Verdam MG, de Haes HC, et al. Development of the Informing Relatives Inventory (IRI): Assessing Index Patients' Knowledge, Motivation and Self-Efficacy Regarding the Disclosure of Hereditary Cancer Risk Information to Relatives. Int J Behav Med. 2015; 22 (4): 551-60.

38. Van der Vaart R, Drossaert CH, Taal E, ten Klooster PM, Hilderink-Koertshuis RT, Klaase JM, et al. Validation of the Dutch functional, communicative and critical health literacy scales. Patient Educ Couns. 2012; 89 (1): 82-8.

39. Inc. S. IBM SPSS Statistics for Windows. Armonk (NY): IBM; 2016.

Authors contributions

LvdH and IC designed the intervention. LvdH, IC, ES and PvT were involved in conception and design of the protocol. LvdH was involved in drafting the manuscript. IC, ES, PvT, YH and AB critically revised the manuscript. All authors were involved in the final approval of the manuscript.

Funding statement

We acknowledge the financial support from the Netherlands Cardiovascular Research Initiative: An initiative with support of the Dutch Heart Foundation, CVON2015-12 eDETECT and CVON2017-10 DOLPHIN-GENESIS.

Competing interests statement

All authors declare they have no competing interests.

Acknowledgements

For peer review only - http://bmjopen.bmj.com/site/about/guidelines.xhtml

For peer review only

Patient advisors are acknowledged for their input regarding the design of this randomized controlled trial.

For peer review only

For peer review only - http://bmjopen.bmj.com/site/about/guidelines.xhtml

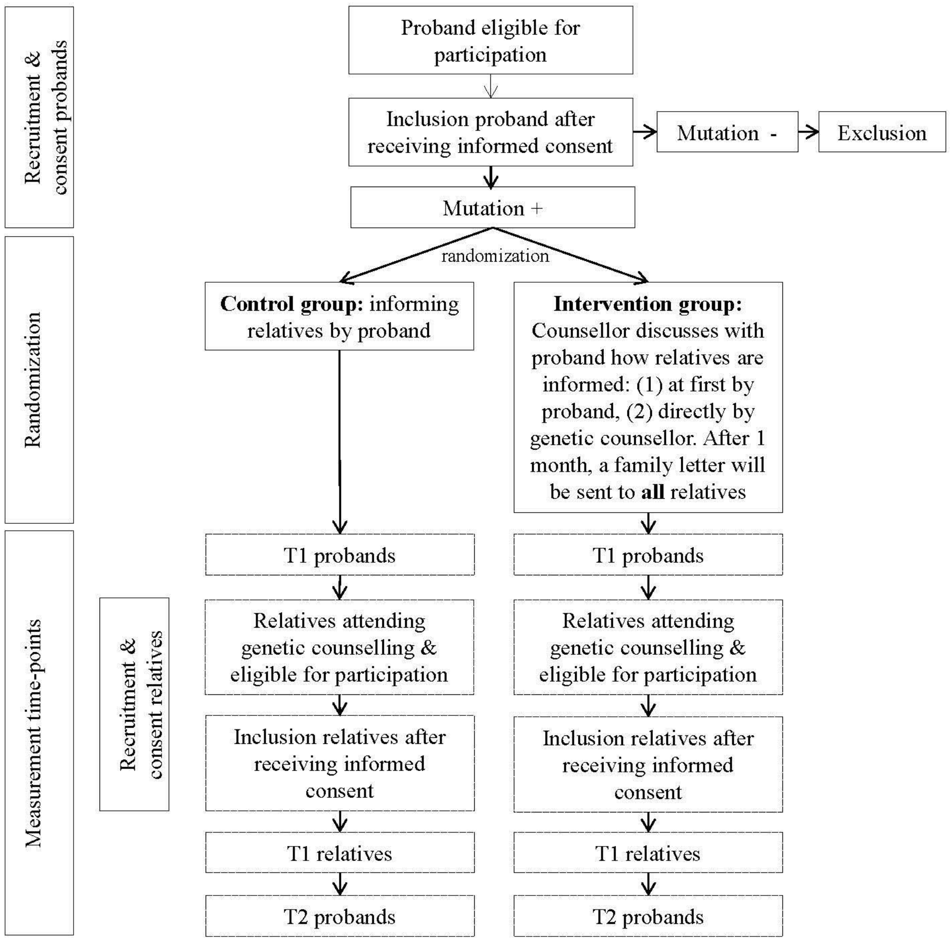

Figure 1 Study procedure

126x130mm (300 x 300 DPI)

Supplementary material

Supplementary material 1: Overview of questionnaire-items to assess secondary outcomes per time-point

Table 1 Questionnaire items probands per time-point

Questionnaire time-point	Items	Questionnaires
T1	Sociodemographic, clinical, family and personality factors	Self-constructed items; trait scale of STAI; shortened version of TMSI
	Advices for relatives at risk, eligible relatives at risk, the number of informed relatives at risk, risk perception and experiences with informing relatives at risk	Eleven self-constructed items
	Evaluation of used approach (incl. website)	Thirteen self-constructed items
	Perceived impact on family communication with relatives at risk	Adapted version of ODCF; one self-constructed item
	Impact on psychological functioning of proband	HADS; adapted version of CWS
T2	Number of informed relatives at risk, risk perception and experiences with informing relatives at risk	Three self-constructed items
	Evaluation of used approach	One self-constructed item
	Perceived impact on family communication with relatives at risk	Adapted version of ODCF; one self-constructed item
	Impact on psychological functioning of proband	HADS; adapted version of CWS

Table 2 Questionnaire items relatives

Questionnaire time-point	Items	Questionnaires
T1	Sociodemographic, family and personality factors	Eight self-constructed items; trait scale of STAI; shortened version of TMSI
	Evaluation of used approach (incl. website), risk perception	Thirteen self-constructed items
	Perceived impact on family communication with index patient	Adapted version of ODCF; one self-constructed item
	Impact on psychological functioning of family member	HADS; adapted version of CWS

Supplementary material 2: Self-constructed items administered in probands and relatives

For peer review only

Table 3 Self-constructed items (telephone interview) - Experiences with informing relatives at risk (probands)

1. Did the genetic counsellor give you an advice for your relatives?<br>2. What was this advice? (open question)<br>3. For which relatives was this advice meant? (open question)<br>4. Have relatives at risk been informed about the advice of the genetic counsellor? If yes, which relatives have been informed?<br>5. Who informed your relatives?	Yes/No<br><br><br>Yes/No

Table 4 Self-constructed items (telephone interview) - Risk perception (probands)

1. How do you consider the risk of your relatives on being a carrier of the familial variant?	0% - 100%<br>1-10
2. How do you estimate the risk of your relatives on developing symptoms of the ICC?	0% - 100%<br>1-10

Table 5 Self-constructed items - Evaluation of the used approach (probands)

T1	*Closed questions*<br>*Below you can see statements regarding your experiences with how your relatives have been informed. Please rate each statement on a scale of 1-5 (1 totally disagree to 5 totally agree) how much each statement applies to you.*	
	1. I feel supported by the genetic counsellor in informing my relatives	1 2 3 4 5
	2. I think the used approach to inform relatives at risk is acceptable	1 2 3 4 5
	3. I felt a little coerced to inform my relatives	1 2 3 4 5
	4. The way my relatives are informed, can be improved	1 2 3 4 5
	5. I am satisfied with the way my relatives are informed	1 2 3 4 5
	Other:________________________________	
-------	---	
T2	1. Did your opinion regarding the used approach change?<br>a. Yes, my opinion regarding the used approach became more positive<br>b. Yes, my opinion regarding the used approach became more negative<br>c. No, my opinion regarding the used approach is still positive<br>d. No, my opinion regarding the used approach is still negative<br>e. No, my opinion regarding the used approach is still neutral	
-------	---	
T1/T2	1. Do you think another approach to inform relatives at risk would have been better?	Yes/No
	2. Are there relatives for which you would have preferred another approach to inform them?	Yes/No
-------	---	
T1/T2	*Open questions*<br>1. What are advantages of the approach used to inform your relatives?	

For peer review only

	2. What are disadvantages of the approach used to inform your relatives?	

Table 6 Self-constructed items - Impact on family relationships (probands)

1. Are there relatives with whom your relationship has changed after they are informed about their risk on the inherited cardiac disease?<br>a. Yes, our relationship improved<br>b. Yes, our relationship worsened<br>c. No, our relationship is still not good/not bad<br>d. No, our relationship is still good<br>e. No, our relationship is still bad

Table 7 Self-constructed items - Evaluation of the used approach (relatives)

Closed questions<br>*Below you can see statements regarding your experiences with how you have been informed about the inherited cardiac disease in your family. Please rate each statement on a scale of 1-5 (1 totally disagree to 5 totally agree) how much each statement applies to you.*	
1. I appreciated to be informed about my risk on the inherited cardiac disease	1 2 3 4 5
2. I am satisfied with the way I have been informed	1 2 3 4 5
3. I preferred to have received more information before I contacted the clinical genetic centre	1 2 3 4 5
4. I understand why I have been informed	1 2 3 4 5
5. The way I have been informed, can be improved	1 2 3 4 5
6. I felt free to decide myself whether I wanted to contact the clinic genetic centre	1 2 3 4 5
7. I would have preferred not to be informed about my risk on the inherited cardiac disease in my family	1 2 3 4 5
8. I would have preferred to not know about the inherited cardiac disease in my family	1 2 3 4 5
Other:________________________________	
--	
1. Do you think another approach to be informed would have been better?	Yes/No
--	
Open questions<br>1. What are advantages of the way you have been informed?<br>2. What are disadvantages of the way you have been informed?	

Table 8 Self-constructed items - Impact on family relationships (relatives)

1. Did your relationship with your relative change after they were informed about their risk on the inherited cardiac disease?<br>a. Yes, our relationship improved<br>b. Yes, our relationship worsened<br>c. No, our relationship is still not good/not bad

For peer review only

d.	No, our relationship is still good
e.	No, our relationship is still bad

For peer review only

SPIRIT
STANDARD PROTOCOL ITEMS: RECOMMENDATIONS FOR INTERVENTIONAL TRIALS

SPIRIT 2013 Checklist: Recommended items to address in a clinical trial protocol and related documents*

Section/item	Item No	Description	Page no
**Administrative information**			
Title	1	Descriptive title identifying the study design, population, interventions, and, if applicable, trial acronym	1
Trial registration	2a	Trial identifier and registry name. If not yet registered, name of intended registry	2
	2b	All items from the World Health Organization Trial Registration Data Set	6-17
Protocol version	3	Date and version identifier	-
Funding	4	Sources and types of financial, material, and other support	18
Roles and responsibilities	5a	Names, affiliations, and roles of protocol contributors	1
	5b	Name and contact information for the trial sponsor	-
	5c	Role of study sponsor and funders, if any, in study design; collection, management, analysis, and interpretation of data; writing of the report; and the decision to submit the report for publication, including whether they will have ultimate authority over any of these activities	-
	5d	Composition, roles, and responsibilities of the coordinating centre, steering committee, endpoint adjudication committee, data management team, and other individuals or groups overseeing the trial, if applicable (see Item 21a for data monitoring committee)	-
**Introduction**			
Background and rationale	6a	Description of research question and justification for undertaking the trial, including summary of relevant studies (published and unpublished) examining benefits and harms for each intervention	4-6
	6b	Explanation for choice of comparators	4-6
Objectives	7	Specific objectives or hypotheses	6

For peer review only

Trial design	8	Description of trial design including type of trial (eg, parallel group, crossover, factorial, single group), allocation ratio, and framework (eg, superiority, equivalence, noninferiority, exploratory)	6

Methods: Participants, interventions, and outcomes

Study setting	9	Description of study settings (eg, community clinic, academic hospital) and list of countries where data will be collected. Reference to where list of study sites can be obtained	6
Eligibility criteria	10	Inclusion and exclusion criteria for participants. If applicable, eligibility criteria for study centres and individuals who will perform the interventions (eg, surgeons, psychotherapists)	7
Interventions	11a	Interventions for each group with sufficient detail to allow replication, including how and when they will be administered	9, 10
	11b	Criteria for discontinuing or modifying allocated interventions for a given trial participant (eg, drug dose change in response to harms, participant request, or improving/worsening disease)	17
	11c	Strategies to improve adherence to intervention protocols, and any procedures for monitoring adherence (eg, drug tablet return, laboratory tests)	-
	11d	Relevant concomitant care and interventions that are permitted or prohibited during the trial	-
Outcomes	12	Primary, secondary, and other outcomes, including the specific measurement variable (eg, systolic blood pressure), analysis metric (eg, change from baseline, final value, time to event), method of aggregation (eg, median, proportion), and time point for each outcome. Explanation of the clinical relevance of chosen efficacy and harm outcomes is strongly recommended	11-14
Participant timeline	13	Time schedule of enrolment, interventions (including any run-ins and washouts), assessments, and visits for participants. A schematic diagram is highly recommended (see Figure)	8, 11, 17
Sample size	14	Estimated number of participants needed to achieve study objectives and how it was determined, including clinical and statistical assumptions supporting any sample size calculations	15, 16
Recruitment	15	Strategies for achieving adequate participant enrolment to reach target sample size	-

Methods: Assignment of interventions (for controlled trials)

Allocation:

Sequence generation	16a	Method of generating the allocation sequence (eg, computer-generated random numbers), and list of any factors for stratification. To reduce predictability of a random sequence, details of any planned restriction (eg, blocking) should be provided in a separate document that is unavailable to those who enrol participants or assign interventions	9
Allocation concealment mechanism	16b	Mechanism of implementing the allocation sequence (eg, central telephone; sequentially numbered, opaque, sealed envelopes), describing any steps to conceal the sequence until interventions are assigned	9
Implementation	16c	Who will generate the allocation sequence, who will enrol participants, and who will assign participants to interventions	9
Blinding (masking)	17a	Who will be blinded after assignment to interventions (eg, trial participants, care providers, outcome assessors, data analysts), and how	9
	17b	If blinded, circumstances under which unblinding is permissible, and procedure for revealing a participant's allocated intervention during the trial	9
**Methods: Data collection, management, and analysis**			
Data collection methods	18a	Plans for assessment and collection of outcome, baseline, and other trial data, including any related processes to promote data quality (eg, duplicate measurements, training of assessors) and a description of study instruments (eg, questionnaires, laboratory tests) along with their reliability and validity, if known. Reference to where data collection forms can be found, if not in the protocol	11-15
	18b	Plans to promote participant retention and complete follow-up, including list of any outcome data to be collected for participants who discontinue or deviate from intervention protocols	-
Data management	19	Plans for data entry, coding, security, and storage, including any related processes to promote data quality (eg, double data entry; range checks for data values). Reference to where details of data management procedures can be found, if not in the protocol	17
Statistical methods	20a	Statistical methods for analysing primary and secondary outcomes. Reference to where other details of the statistical analysis plan can be found, if not in the protocol	16, 17
	20b	Methods for any additional analyses (eg, subgroup and adjusted analyses)	16, 17
	20c	Definition of analysis population relating to protocol non-adherence (eg, as randomised analysis), and any statistical methods to handle missing data (eg, multiple imputation)	16, 17

Methods: Monitoring

Data monitoring	21a	Composition of data monitoring committee (DMC); summary of its role and reporting structure; statement of whether it is independent from the sponsor and competing interests; and reference to where further details about its charter can be found, if not in the protocol. Alternatively, an explanation of why a DMC is not needed	NA
	21b	Description of any interim analyses and stopping guidelines, including who will have access to these interim results and make the final decision to terminate the trial	17
Harms	22	Plans for collecting, assessing, reporting, and managing solicited and spontaneously reported adverse events and other unintended effects of trial interventions or trial conduct	-
Auditing	23	Frequency and procedures for auditing trial conduct, if any, and whether the process will be independent from investigators and the sponsor	NA

Ethics and dissemination

Research ethics approval	24	Plans for seeking research ethics committee/institutional review board (REC/IRB) approval	17
Protocol amendments	25	Plans for communicating important protocol modifications (eg, changes to eligibility criteria, outcomes, analyses) to relevant parties (eg, investigators, REC/IRBs, trial participants, trial registries, journals, regulators)	-
Consent or assent	26a	Who will obtain informed consent or assent from potential trial participants or authorised surrogates, and how (see Item 32)	8, 9
	26b	Additional consent provisions for collection and use of participant data and biological specimens in ancillary studies, if applicable	NA
Confidentiality	27	How personal information about potential and enrolled participants will be collected, shared, and maintained in order to protect confidentiality before, during, and after the trial	17
Declaration of interests	28	Financial and other competing interests for principal investigators for the overall trial and each study site	18
Access to data	29	Statement of who will have access to the final trial dataset, and disclosure of contractual agreements that limit such access for investigators	17
Ancillary and post-trial care	30	Provisions, if any, for ancillary and post-trial care, and for compensation to those who suffer harm from trial participation	NA

For peer review only

Dissemination policy	31a	Plans for investigators and sponsor to communicate trial results to participants, healthcare professionals, the public, and other relevant groups (eg, via publication, reporting in results databases, or other data sharing arrangements), including any publication restrictions	17
	31b	Authorship eligibility guidelines and any intended use of professional writers	18
	31c	Plans, if any, for granting public access to the full protocol, participant-level dataset, and statistical code	-
**Appendices**			
Informed consent materials	32	Model consent form and other related documentation given to participants and authorised surrogates	-
Biological specimens	33	Plans for collection, laboratory evaluation, and storage of biological specimens for genetic or molecular analysis in the current trial and for future use in ancillary studies, if applicable	NA

*It is strongly recommended that this checklist be read in conjunction with the SPIRIT 2013 Explanation & Elaboration for important clarification on the items. Amendments to the protocol should be tracked and dated. The SPIRIT checklist is copyrighted by the SPIRIT Group under the Creative Commons "Attribution-NonCommercial-NoDerivs 3.0 Unported" license.

For peer review only

BMJ Open

A tailored approach towards informing relatives at risk of inherited cardiac conditions: study protocol for a randomised controlled trial

Journal:	*BMJ Open*
Manuscript ID	bmjopen-2018-025660.R1
Article Type:	Protocol
Date Submitted by the Author:	10-Dec-2018
Complete List of Authors:	van den Heuvel, Lieke; Amsterdam University Medical Centres, Department of Clinical Genetics<br>Hoedemaekers, Yvonne; University Medical Centre Groningen, Department of Clinical Genetics<br>Baas, Annette; University Medical Center Utrecht, Department of Genetics<br>van Tintelen, J; Amsterdam University Medical Centres, Department of Clinical Genetics<br>Smets, Ellen; Amsterdam University Medical Centres, Department of Medical Psychology<br>Christiaans, Imke; Amsterdam University Medical Centres, Clinical Genetics
**Primary Subject Heading**:	Genetics and genomics
Secondary Subject Heading:	Communication, Ethics, Genetics and genomics, Cardiovascular medicine, Health policy
Keywords:	Inherited cardiac conditions, Cardiogenetics, Informing relatives at risk, Family-mediated approach, Tailored approach, Randomized controlled trial

SCHOLARONE™
Manuscripts

A tailored approach towards informing relatives at risk of inherited cardiac conditions: study protocol for a randomised controlled trial

L.M. van den Heuvel[1], Y.M. Hoedemaekers[2], A.F. Baas[3], J.P. van Tintelen[1], E.M.A. Smets[4]& I. Christiaans[1*]

[1]Department of Clinical Genetics, Amsterdam University Medical Centres / University of Amsterdam, Amsterdam, the Netherlands

[2]Department of Genetics, University of Groningen, University Medical Centre Groningen, Groningen, the Netherlands

[3]Department of Genetics, University Medical Centre Utrecht / University Utrecht, Utrecht, the Netherlands

[4]Department of Medical Psychology, Amsterdam University Medical Centres / University of Amsterdam, Amsterdam, the Netherlands

* Corresponding author

Department of Clinical Genetics,

Amsterdam University Medical Centre, location AMC

Meibergdreef 9, 1105 AZ, Amsterdam, the Netherlands

Telephone: +31 20-5667217

E-mail: i.christiaans@amc.nl

For peer review only - http://bmjopen.bmj.com/site/about/guidelines.xhtml

Abstract

Introduction In current practice, probands are asked to inform relatives about the possibility of predictive DNA testing when a pathogenic variant causing an inherited cardiac condition (ICC) is identified. Previous research on the uptake of genetic counselling and predictive DNA testing in relatives suggests that not all relatives are sufficiently informed. We developed a randomised controlled trial to evaluate the effectiveness of a tailored approach in which probands decide together with the genetic counsellor which relatives they inform themselves and which relatives they prefer to have informed by the genetic counsellor. Here, we present the study protocol of this randomised controlled trial. **Methods** A multicentre randomised controlled trial with parallel-group design will be conducted in which an intervention group receiving the tailored approach will be compared to a control group receiving usual care. Adult probands diagnosed with an ICC in whom a likely pathogenic or pathogenic variant is identified, will be randomly assigned to the intervention or control group (total sample: n = 85 probands). Primary outcomes are uptake of genetic counselling and predictive DNA testing by relatives (total sample: n = 340 relatives). Secondary outcomes are appreciation of the approach used and impact on familial and psychological functioning, which will be assessed using questionnaires. Relatives who attend genetic counselling will be asked to fill out a questionnaire as well. **Ethics and dissemination** Ethical approval was obtained from the Medical Ethical Committee of the Amsterdam University Medical Centres (MEC 2017-145), the Netherlands. All participants will provide informed consent prior to participation in the study. Results of the study on primary and secondary outcome measures will be published in peer-reviewed journals. **Registration details** This trial is registered at the Netherlands Trial Register NTR6657.

Key words Inherited cardiac conditions, cardiogenetics, informing relatives at risk, family-mediated approach, tailored approach, randomised controlled trial

For peer review only - http://bmjopen.bmj.com/site/about/guidelines.xhtml

Strengths and limitations of this study

- This randomised controlled trial investigates both the uptake of genetic counselling and of predictive DNA testing, as well as the acceptance and impact on psychological and family functioning in the tailored versus the standard approach, in probands and relatives.
- This study will be conducted in three clinical genetics clinics with expertise on cardiogenetics, which will facilitate participant inclusion.
- In this trial, evaluation of the effect on outcome of the different components of the intervention is not possible, due to limited power.
- In this randomised controlled trial it is not possible to blind participants, genetic counsellors or the executing investigator for the chosen intervention.
- Because a baseline measure for the secondary outcomes is not possible, we cannot control for likely confounding factors, such as intention to inform at-risk relatives, and family and psychological functioning at baseline.

For peer review only - http://bmjopen.bmj.com/site/about/guidelines.xhtml

Introduction

Inherited cardiac conditions (ICCs) such as cardiomyopathies and primary arrhythmia syndromes generally demonstrate an autosomal dominant inheritance pattern and a wide variety of symptoms that can manifest at any age [1, 2]. One feared outcome is sudden cardiac death (SCD), which can occur at a young age and be the first symptom of disease [3, 4]. With an incomplete penetrance and high variability in expression even within families, carriers of a familial variant may remain undetected but still be at risk for SCD even though treatment options are available that prevent disease progression or potentially life-threatening arrhythmias [5]. Predictive DNA testing is therefore offered to first-degree relatives of probands (the first person in a family diagnosed with an ICC) in whom a pathogenic variant is identified because these relatives are at 50% risk of also having inheriting the genetic variant [5, 6]. Predictive DNA testing is offered to relatives in a stepwise manner (cascade screening), with the aim of identifying asymptomatic carriers of the familial variant to facilitate timely treatment. Non-carriers of the familial variant generally do not need cardiac monitoring and can be reassured about their own risk and that of their offspring [6].

In current practice in the Netherlands, probands are asked to inform their relatives, supported by a family letter written by the genetic counsellor. This is referred to as the family-mediated approach [7]. Previous research, however, shows that uptake (the number of relatives at risk attending genetic counselling and/or undergoing predictive DNA testing) is relatively low in ICCs, particularly for cardiomyopathies. Reported uptakes are around 50% despite family letters being provided to a majority of relatives by the proband [8-10]. Previous research in other genetic patient populations, such as hereditary types of cancer, shows similar uptake percentages [11-13].

Some relatives who do not attend genetic counselling will have deliberately decided against predictive DNA testing. However, the low uptake percentages also suggest that many

For peer review only - http://bmjopen.bmj.com/site/about/guidelines.xhtml

relatives may be unaware, or insufficiently aware, of the risks involved and/or the possibilities for genetic counselling and subsequent surveillance and treatment. This is supported by research on family communication in ICCs. Patients are not always able to inform or correctly inform their relatives for a number of reasons, including disengagement with relatives, lack of understanding of the importance of the information, preoccupation with their own grief, difficulties in conveying the complex information to relatives, or a wish to prevent burdening relatives by informing them about genetic risks [8, 14-18].

Previous studies assessing interventions to enhance family communication in hereditary diseases showed that some interventions are effective in increasing the uptake of genetic counselling [19-21]. An intervention trial aimed at improving family communication in specifically dilated cardiomyopathy is still ongoing [22]. A few studies have been published on more active approaches to informing relatives at risk in which healthcare professionals (HCPs) contact at-risk relatives directly [23-26]. These studies suggest that a more active approach can almost double the uptake of genetic counselling and predictive DNA testing by relatives (23-26) However, some of these studies were performed in a research setting (e.g. in relatives already registered in research databases for the genetic disease), hampering direct translation of these results to a diagnostic setting. To our knowledge, more active approaches in patients with ICCs have not been studied thus far. However, a study by Ormondroyd et al [14] suggests that relatives eligible for predictive DNA testing for hypertrophic cardiomyopathy and long QT syndrome would support a more active approach to informing relatives at risk.

Although studies on more active approaches did not report any psychological harm in relatives at group level, these approaches could cause more unwarranted worry or pressure on relatives to opt for predictive DNA testing [23-25]. An active approach to informing relatives at risk could also breach the autonomy and confidentiality of probands, and may harm

For peer review only - http://bmjopen.bmj.com/site/about/guidelines.xhtml

relative's right not to know [27-29]. Furthermore, HCPs are often unaware of interpersonal dynamics within families and the personal circumstances of relatives at risk. Active approaches may therefore have a negative impact on family relationships or may cause psychological distress in both probands and relatives [30].

Because of this, a tailored approach in which a proband decides together with the genetic counsellor which at-risk relatives he or she will inform and which relatives he or she prefers to be informed by the genetic counsellor could be optimal. With this approach, the probands expert knowledge of a relative's functioning and of family dynamics could be used appropriately, and the autonomy of the proband preserved. At the same time, more relatives at risk would be sufficiently informed [28, 30]. Furthermore, probands for whom informing relatives is difficult or burdensome might be relieved or supported by this approach [30].

Objectives

The primary aim of this randomised controlled trial is to assess whether uptake of genetic counselling and testing of relatives at risk of an ICC will be increased by using a tailored approach to information provision for relatives, instead of usual care (i.e. the family-mediated approach). Secondary objectives are to evaluate how such a tailored approach is appreciated by both probands and relatives as compared to usual care. In addition, this study aims to assess the perceived impact on family relationships and psychological functioning of both probands and relatives. The protocol presented here has been described based on the SPIRIT statement [31]

Methods

Design

A multicentre randomised controlled trial with a parallel-group design will be conducted in three university hospitals in the Netherlands (the Amsterdam University Medical Centres

For peer review only - http://bmjopen.bmj.com/site/about/guidelines.xhtml

(Amsterdam UMC), the University Medical Centre Utrecht (UMCU) and the University Medical Centre Groningen (UMCG)) to compare the effects of a tailored approach to informing relatives at risk of ICCs to usual care in both probands and relatives.

Participants

All probands aged 18 years or older with an ICC, or suspicion thereof, attending pre-test genetic counselling at the cardiogenetics outpatient clinics during the inclusion period will be asked to participate if they: (1) are the first member of their family to visit the cardiogenetics outpatient clinic for counselling about genetic testing for ICCs; (2) they have at least one living adult relative; and (3) are able to read and write Dutch. Only probands in whom a likely pathogenic or pathogenic variant is detected (class 4 - likely pathogenic or class 5 - pathogenic variant) will be definitively included.

In addition, eligible adult first- (or second-) degree relatives of enrolled probands who make an appointment at the cardiogenetics outpatient clinics will be invited to fill out a questionnaire to measure secondary outcomes. Inclusion criteria are defined as follows: (1) first-degree adult (18 years or older) relatives of probands enrolled in the study or second-degree adult relatives in case of a deceased connecting first-degree relative who was affected or suspected to be affected, and (2) able to read and write Dutch.

Procedure

Figure 1 shows a flowchart of the study procedure.

Recruitment & consent

During pre-test genetic counselling, the genetic counsellor will inform the probands about the study and provide an informational letter (see Supplementary Material S1). In addition, probands will be asked if the executing researcher can contact them to provide further

For peer review only - http://bmjopen.bmj.com/site/about/guidelines.xhtml

information about the study. Subsequently, probands will be contacted by telephone by the executing researcher. If probands are still interested in participation, written informed consent forms will be sent by post, including a return envelope. As described above, only probands in whom a likely pathogenic or pathogenic variant is detected will be definitively included in the study.

Relatives of enrolled probands attending pre-test genetic counselling in one of the participating centres who are also at risk will also be invited to participate in the study. The same recruitment procedure will be used.

Randomisation

Prior to receiving their test result, probands with an ICC in whom a likely pathogenic or pathogenic variant is identified will be randomly assigned to either the intervention or control group. Block randomisation will be used, with variable blocks ranging from size two to six. Randomisation will be stratified for gender, disease type (cardiomyopathies or primary arrhythmia syndromes) and hospital. To ensure allocation concealment, computer software will be used for randomisation, with an allocation rate of 1:1 [32]. Relatives of probands included in the study will be assigned to the group to which the proband was assigned.

Neither participants nor genetic counsellors will or can be blinded for group assignment. The executing researcher also cannot be blinded because of slight differences between the questionnaires administered in the intervention- and control groups. Part of the outcome data will be collected using telephone interviews. To minimize bias, these interviews will be conducted by a research assistant following a structured script.

<u>Intervention group</u>

For peer review only - http://bmjopen.bmj.com/site/about/guidelines.xhtml

In the intervention group, a tailored approach to informing relatives at risk will be provided. In this approach, probands with a likely pathogenic or pathogenic variant will discuss with the genetic counsellor which relatives are at risk of inheriting the familial variant. They will then be asked which of these relatives they prefer to inform themselves at first using a family letter written by the genetic counsellor, and which relatives they prefer to be directly informed by the genetic counsellor with a similar family letter. This will be discussed during routine post-test counselling. In both cases, after one month, the genetic counsellor will send the family letter directly to all relatives at risk for whom the proband has provided consent to contact. The proband will be asked to provide contact details of these relatives.

The family letter is standardised for all three participating centres. For the intervention group, the letter also includes a link to a website specifically designed for this study where relatives can find additional information (www.familieleden.erfelijkehartziekten.nl). The information on this website will be tailored to relative's situations (i.e. specified for disease-type, hospital, parenthood, whether relatives have a desire to have children in the future, and which information relatives prefer to receive) by asking them to fill out a short questionnaire on their first visit to the website.

<u>Control group</u>

In the control group, the standard care approach will be used. If a likely pathogenic or pathogenic variant is identified, probands assigned to the control group will be asked by the genetic counsellor to inform relatives at risk about the genetic test result, the consequences of this result for relatives and the advice regarding predictive DNA testing and/or cardiac monitoring. This will be discussed during routine post-test counselling. Probands will be supported in informing relatives at risk by a family letter written by the genetic counsellor. This family letter is also standardised for all three participating centres. However, this letter

For peer review only - http://bmjopen.bmj.com/site/about/guidelines.xhtml

does not include the link to the website with tailored information described above, but does include a link to a general website on ICCs (www.erfelijkehartziekten.nl).

Measurement time-points

For secondary outcome measures, participating probands will be asked to complete a questionnaire one month after receiving the genetic test result (T1) and to complete a second questionnaire nine months after the test result (T2). Before T1 and T2, a short structured telephone interview will be conducted about participant's knowledge of which relatives are at risk of ICCs and which relatives are informed, because these items are expected to be too complex to answer in a questionnaire [33]. Participating relatives will complete one questionnaire after attending genetic counselling.

Measures

Primary outcome measures

To assess the effect of a tailored approach to informing relatives at risk, the difference between the intervention and control groups in uptake of (1) genetic counselling, and (2) predictive DNA testing of relatives at risk will be measured. To do this, the number of relatives attending genetic counselling and the number of relatives who are genetically tested in the first year after detection of the likely pathogenic or pathogenic variant in the proband will be collected in the laboratories of each participating centre. DNA test results of relatives counselled in non-participating centres will also be taken into account because, in the Netherlands, predictive DNA testing of relatives is always performed in the same laboratory where the proband was tested.

The number of relatives attending genetic counselling and undergoing predictive DNA testing will be compared to the total number of relatives at risk of inheriting the variant who are eligible for genetic counselling and predictive DNA testing based on family pedigrees. For

For peer review only - http://bmjopen.bmj.com/site/about/guidelines.xhtml

relatives who attend genetic counselling but decide against predictive DNA testing, subsequent attendance of cardiac monitoring will be checked.

Relatives at risk who are eligible for genetic counselling and predictive DNA testing are first-degree relatives and second-degree relatives if there is a connecting deceased first-degree relative suspected of having an ICC. Following the Dutch clinical guidelines for cardiomyopathies, relatives at risk are eligible for genetic counselling and predictive DNA testing from the age of 10 years. For primary arrhythmias, depending on the specific arrhythmic disorder, relatives at risk are eligible for predictive DNA testing from birth.

Furthermore, conditional uptake of relatives at risk, defined as the number of relatives who are genetically tested relative to the number who attend genetic counselling, will be calculated. Uptake will be measured at randomisation condition (intervention or control group) and family level.

Secondary outcome measures

Secondary outcome measures will be measured using both validated and self-constructed questionnaire items. An overview of these items is shown in the Supplementary Material S2. Secondary outcome measures include the following:

- Appreciation of the information provision strategy used and preferences regarding the approach used to inform relatives at risk: This will be evaluated in both probands and relatives using self-constructed items on a 5 point Likert scale (1 = 'Totally disagree' to 5 = 'Totally agree') in a questionnaire (probands: 5 items, range 5-25; relatives: 6 items, range 6-30). Probands will be asked to answer an additional self-constructed item during the structured telephone interview about whether they would have preferred to inform their relatives differently. Two additional self-constructed items will be administered in the intervention group to assess decisional conflict in probands, including whether

For peer review only

probands thought it was difficult to choose to inform their relatives themselves or have them informed by the counsellor, and whether they were satisfied by their decision, on a 5 point Likert scale (1 = 'Totally disagree' to 5 = 'Totally agree'; range 2-10). Probands will be asked to fill out these items at T1. At T2, a self-constructed item will be administered to assess whether their opinion regarding the approach used has changed. Finally, probands (at T1 and T2) and relatives will be asked whether they visited the website www.erfelijkehartziekten.nl and, if yes, how they evaluated the website, using four self-constructed items on a 5 point Likert scale (1 = 'Totally disagree' to 5 = 'Totally agree'; range 4-20).

- Impact on family communication: To assess the impact of the tailored approach versus the usual care approach on family functioning, probands (at T1 and T2) and relatives will be asked to fill out an adapted version of the Openness to Discuss Cancer in the Family (ODCF) scale, which assesses communication about genetic risks within families with nine items on a 5 point Likert scale (1 = 'Totally disagree' to 5 = 'Totally agree'; range 9-45) [34]. Psychometric characteristics of the original ODCF scale are satisfactory [34]. In addition, a self-constructed item will be administered asking about the nature of regular communication with relatives and whether probands and relatives experienced changes in their relationships with relatives as a consequence of the information provision process.
- Impact on psychological functioning: To assess the impact on psychological functioning, two validated questionnaires will be administered in probands (at T1 and T2) and relatives. Participants will be asked to fill out an adapted version of the Cancer Worry Scale (CWS) [35]. The CWS was developed and validated in Dutch patients with hereditary types of cancer [35]. Because it was validated in a Dutch patient population and is previously used in a genetic patient population, it was considered the most appropriate scale for this randomised controlled trial. The CWS consists of eight items on a 4 point

For peer review only - http://bmjopen.bmj.com/site/about/guidelines.xhtml

Likert scale (1 = ‘Almost never’ to 4 = ‘Almost always’; range 8-32). Psychometric characteristics of the CWS have been assessed in a sample of breast cancer survivors, and support its reliability and validity [35].

In addition, the Hospital Anxiety and Depression Scale (HADS) will be administered to assess whether participants experience anxious or depressed feelings after being informed about the hereditary disease [36]. The HADS contains two 7-item subscales on a 4 point Likert scale with diverse answer options that assess both anxiety and depression with a score range of 0-21. Psychometric characteristics of the HADS were assessed as good [36].

Participants' characteristics

To assess whether randomisation succeeded and whether characteristics of participating probands and relatives have influenced the primary and secondary outcome measures, sociodemographic and clinical factors will be collected, including gender, education level, ethnicity, living situation and parenthood, family history and the diagnosis of the probands at T1. Relatives will additionally be asked what their degree of kinship is with the proband.

For the same reason, psychosocial and personality factors will be assessed in both probands (at T1) and relatives. Coping style will be assessed by using the shortened version of the Threatening Medical Situations Inventory (TMSI) [37, 38]. The TMSI assesses a “monitoring” versus “blunting” coping style related to a medical threat, and it was previously evaluated in an oncogenetic patient population [37, 39]. The shortened version of the TMSI contains two subscales, both consisting of six items on a 5 point Likert scale (1 = ‘Totally not applicable’ to 5 = ‘Totally applicable’; range 6-30). Reliability and validity are satisfactory [37, 38]. The Trait subscale of the State Trait Anxiety Inventory (STAI) will be administered to assess trait anxiety in both probands and relatives [40]. The STAI is frequently used in

For peer review only - http://bmjopen.bmj.com/site/about/guidelines.xhtml

research settings and consists of 20 items on a 4 point Likert scale (1 = 'Not at all' to 4 = 'Very much so'; range 20-80) [40]. The reliability and validity for the Dutch translation of the STAI are assessed as good [41].

Self-efficacy and perceived motivators and barriers regarding informing relatives at risk will be assessed using an adapted version of the 'motivation' and 'self-efficacy' subscales of the Informing Relatives Inventory (IRI) [42]. The IRI was developed and evaluated in an oncogenetic patient population, and showed satisfactory reliability and validity [42]. The 'motivation' subscale consists of 30 items on a 5 point Likert scale (1 = 'No role' to 5 = 'A large role'; range 30-150); the 'self-efficacy' subscale of 7 items on a 4 point Likert scale (1 = 'Not sure at all' to 4 = 'Very sure'; range 7-21). Probands will also be asked to answer a self-constructed item during the telephone interviews regarding whether relatives were informed and whether probands intended to inform (remaining) at-risk relatives.

Risk perception regarding the risk of relatives carrying the variant and developing the disease will be assessed by using self-constructed items. These items ask participants to rate the perception of the risk of relatives carrying the variant and developing the disease on a scale from 1 (lowest risk) to 10 (highest risk) as well as from 0% (lowest risk) to 100% (highest risk).

Health literacy – defined as the ability to obtain, process and understand basic health information and services – will be assessed in probands and relatives using the items on the 'functional health literacy' and 'communicative health literacy' subscales of the 3HL questionnaire [43]. Both subscales contain five items on a 4 point Likert scale (1 = 'Never' to 4 = 'Often'; range per subscale 5-20). The reliability for both scales was assessed as high and the validity as satisfactory [43].

Sample size calculation

For peer review only - http://bmjopen.bmj.com/site/about/guidelines.xhtml

The study aims to detect a difference of 15% in uptake of genetic counselling by relatives between the control (usual care, 50%) and intervention groups (tailored approach, 65%). Assuming a two-sided 5% significance level and a power of 80%, 340 relatives (170 in each group) would be required to participate in this study. On average, six relatives per proband are at 50% risk of inheriting the variant, including children and adults [9]. With a conservative estimate of four eligible adult relatives per proband at risk, 85 probands with an ICC and an identified likely pathogenic or pathogenic (class 4 or 5) variant will need to be included in this study to reach 340 relatives. A likely pathogenic or pathogenic variant is found in, on average, 20% (lower margin) of all probands with a suspected ICC. With an expected response rate of 70% and a drop-out rate of 20%, approximately 759 probands will be approached to participate in the study.

Data analysis

Statistical analysis

Sociodemographic, clinical, psychosocial and personality variables will be analysed using descriptive and frequency statistics. An intention-to-treat approach will be used. SPSS version 24.0 will be used to perform statistical analyses [44]. An α level of $p < .05$ will be used. Analysis of Variance and chi-square tests will be used to assess differences (i) in sociodemographic, clinical and psychological characteristics between the intervention- and control group and (ii) in participants and non-participants, as appropriate. Descriptive and frequency statistics will be used to describe the primary outcomes: (1) uptake of genetic counselling and (2) uptake of predictive DNA testing. Logistic regression analysis will be conducted to assess differences between the intervention- and control group on the primary outcomes. Multilevel analyses will be performed to assess whether the intervention has an impact on family and psychological functioning. The two measurement time-points will be

For peer review only - http://bmjopen.bmj.com/site/about/guidelines.xhtml

treated as nested within probands. To prevent influence of potential confounding factors, analysis will be adjusted for covariates (i.e., sociodemographic, clinical and psychological variables). Participant appreciation of the approach used will be described using frequency statistics.

Qualitative analysis

Open questions will be analysed using thematic analysis based on the principles of Braun and Clarke [45]. Analysis software for qualitative data, MAXQDA version 12, will be used [46]. Coding analysis will be conducted by two trained coders independently. The codes will be analysed and interpreted to create a structure of themes and subthemes. The qualitative results will be used to supplement the questionnaire data.

Patient and public involvement

Prior to this randomised controlled trial, face-to-face interviews were conducted with probands and counselled relatives (both carriers and non-carriers) to explore their experiences with and preferences regarding informing at-risk relatives (unpublished). In addition, online focus groups were conducted with HCPs. The randomised controlled trial was then designed based on the findings of both these interviews and focus groups. Since this study is part of the eDETECT research consortium (CVON2015-12), several patient representative groups (the PLN foundation; Harteraad, Heartz) participated in the user committee and scientific meetings and thereby gave input to this research proposal. Patients are not involved in the recruitment and conduct of the study.

During patient seminars, patients will be updated on the progress and results of the study. In addition, during the eDETECT scientific meetings, all participants of the eDETECT consortium (including representatives of the aforementioned patient organisations) will be

For peer review only - http://bmjopen.bmj.com/site/about/guidelines.xhtml

informed. After completion of the study, group results will be disseminated by e-mail to study participants who indicated their interest in the outcome during informed consent. A summary of the results will also be posted on the ICCs website (www.erfelijkehartziekten.nl).

The burden of the intervention was not assessed because this is an intrinsic part of the outcome measures of this study. The patients themselves were involved in pilot testing the questionnaires used to assess these outcome measures.

Ethics and dissemination

The Medical Ethical Committee of the Amsterdam UMC has approved the study design (MEC 2017-145). Additional approval of regional Medical Ethical Committees of the other participating academic centres has also been obtained. Informed consent is required from each participant. Participants who provide written informed consent can withdraw from the study at any time, without providing a reason.

After receiving informed consent, a unique research ID will be assigned to the participant. Only this ID will be used to identify research documents. Each research document will be saved on a secured server. The principal investigator, coordinating investigator and executing investigator have access to this secured server. Research documents will be saved for a period of 15 years. This randomised controlled trial is registered at the Netherlands Trial Register NTR6657. Separate manuscripts with findings on, respectively, the primary and secondary outcomes will be published in peer-reviewed journals.

Trial status

Recruitment of probands during pre-test genetic counselling for this randomised controlled trial started in November 2017. In total, recruitment of probands will last one year.

For peer review only - http://bmjopen.bmj.com/site/about/guidelines.xhtml

Subsequent uptake of genetic counselling and predictive DNA testing will be measured until one year after the detection of a pathogenic variant in the proband. Data collection will therefore continue until January 2020, taking into account a duration of, on average, three months for the DNA-test result in the proband to be available. To date, 68 probands have been included and randomised to either the intervention or the control group. In addition, 49 relatives consented to participate.

References

1. Theilade J, Kanters J, Henriksen FL, et al. Cascade screening in families with inherited cardiac diseases driven by cardiologists: feasibility and nationwide outcome in long QT syndrome. Cardiology. 2013; 126 (2): 131-7. DOI: 10.1159/000350825.

2. Watkins H, Ashrafian H, Redwood C. Inherited cardiomyopathies. N Engl J Med. 2011; 364 (17): 1643-56. DOI: 10.1056/NEJMra0902923.

3. Behr ER, Dalageorgou C, Christiansen M, et al. Sudden arrhythmic death syndrome: familial evaluation identifies inheritable heart disease in the majority of families. Eur Heart J. 2008; 29 (13): 1670-80. DOI: 10.1093/eurheartj/ehn219.

4. Van der Werf C, Hofman N, Tan HL, et al. Diagnostic yield in sudden unexplained death and aborted cardiac arrest in the young: the experience of a tertiary referral center in The Netherlands. Heart Rhythm. 2010; 7 (10): 1383-9. DOI: 10.1016/j.hrthm.2010.05.036.

5. Wilde AA, Behr ER. Genetic testing for inherited cardiac disease. Nat Rev Cardiol. 2013; 10 (10): 571-83. DOI: 10.1038/nrcardio.2013.108.

6. Miller EM, Wang Y, Ware SM. Uptake of cardiac screening and genetic testing among hypertrophic and dilated cardiomyopathy families. J Genet Couns. 2013; 22 (2) :258-67. DOI: 10.1007/s10897-012-9544-4.

For peer review only - http://bmjopen.bmj.com/site/about/guidelines.xhtml

7. Leenen CH, Heijer M, van der Meer C, Kuipers EJ, van Leerdam ME, Wagner A. Genetic testing for Lynch syndrome: family communication and motivation. Fam Cancer. 2016; 15 (1): 63-73. DOI: 10.1007/s10689-015-9842-8.

8. Burns C, McGaughran J, Davis A, Semsarian C, Ingles J. Factors influencing uptake of familial long QT syndrome genetic testing. Am J Med Genet A. 2016; 170A (2): DOI: 10.1002/ajmg.a.37455.

9. Christiaans I, Birnie E, Bonsel GJ, Wilde AA, van Langen IM. Uptake of genetic counselling and predictive DNA testing in hypertrophic cardiomyopathy. Eur J Hum Genet. 2008; 16 (10): 1201-7. DOI: 10.1038/ejhg.2008.92.

10. Van der Roest WP, Pennings JM, Bakker M, van den Berg MP, van Tintelen JP. Family letters are an effective way to inform relatives about inherited cardiac disease. Am J Med Genet A. 2009; 149A (3): 357-63. DOI: 10.1002/ajmg.a.32672.

11. Brooks L, Lennard F, Shenton A, et al. BRCA1/2 predictive testing: a study of uptake in two centres. Eur J Hum Genet. 2004; 12 (8): 654-62.

12. Menko FH, Ter Stege JA, van der Kolk LE, et al. The uptake of presymptomatic genetic testing in hereditary breast-ovarian cancer and Lynch syndrome: a systematic review of the literature and implications for clinical practice. Fam Cancer. 2018. DOI: 10.1007/s10689-018-0089-z.

13. Ramsoekh D, van Leerdam ME, Tops CM, et al. The use of genetic testing in hereditary colorectal cancer syndromes: genetic testing in HNPCC, (A)FAP and MAP. Clin Genet. 2007; 72(6): 562-7.

14. Ormondroyd E, Oates S, Parker M, Blair E, Watkins H. Pre-symptomatic genetic testing for inherited cardiac conditions: a qualitative exploration of psychosocial and ethical implications. Eur J Hum Genet. 2014; 22 (1): 88-93. DOI: 10.1038/ejhg.2013.81.

For peer review only - http://bmjopen.bmj.com/site/about/guidelines.xhtml

15. Smart A. Impediments to DNA testing and cascade screening for hypertrophic cardiomyopathy and Long QT syndrome: a qualitative study of patient experiences. J Genet Couns. 2010; 19 (6): 630-9. DOI: 10.1007/s10897-010-9314-0.

16. Vavolizza RD, Kalia I, Erskine Aaron K, et al. Disclosing Genetic Information to Family Members About Inherited Cardiac Arrhythmias: An Obligation or a Choice? J Genet Couns. 2015; 24 (4): 608-15. DOI: 10.1007/s10897-014-9783-7.

17. Whyte S, Green A, McAllister M, Shipman H. Family Communication in Inherited Cardiovascular Conditions in Ireland. J Genet Couns. 2016; 25 (6): 1317-26. DOI: 10.1007/s10897-016-9974-5.

18. Batte B, Sheldon JP, Arscott P, et al. Family communication in a population at risk for hypertrophic cardiomyopathy. J Genet Couns. 2015; 24 (2): 336-48. DOI: 10.1007/s10897-014-9774-8.

19. Forrest LE, Burke J, Bacic S, Amor DJ. Increased genetic counseling support improves communication of genetic information in families. Genet Med. 2008; 10 (3): 167-72. DOI: 10.1097/GIM.0b013e318164540b.

20. Hodgson JM, Metcalfe SA, Aitken M, et al. Improving family communication after a new genetic diagnosis: a randomised controlled trial of a genetic counselling intervention. BMC Med Genet. 2014; 15: 33. DOI: 10.1186/1471-2350-15-33.

21. Hodgson JM, Metcalfe S, Gaff C, et al. Outcomes of a randomised controlled trial of a complex genetic counselling intervention to improve family communication. Eur J Hum Genet. 2016; 24 (3): 356-60. DOI: 10.1038/ejhg.2015.122.

22. Kinnamon DD, Morales A, Bowen DJ, Burke W, Hershberger RE, Consortium* DCM. Toward Genetics-Driven Early Intervention in Dilated Cardiomyopathy: Design and Implementation of the DCM Precision Medicine Study. Circ Cardiovasc Genet. 2017; 10 (6). DOI: 10.1161/CIRCGENETICS.117.001826.

For peer review only - http://bmjopen.bmj.com/site/about/guidelines.xhtml

23. Aktan-Collan K, Haukkala A, Pylvanainen K, Jarvinen HJ, Aaltonen LA, Peltomaki P, et al. Direct contact in inviting high-risk members of hereditary colon cancer families to genetic counselling and DNA testing. J Med Genet. 2007; 44 (11): 732-8.

24. Sermijn E, Delesie L, Deschepper E, et al. The impact of an interventional counselling procedure in families with a BRCA1/2 gene mutation: efficacy and safety. Fam Cancer. 2016; 15 (2): 155-62. DOI: 10.1007/s10689-015-9854-4.

25. Suthers GK, Armstrong J, McCormack J, Trott D. Letting the family know: balancing ethics and effectiveness when notifying relatives about genetic testing for a familial disorder. J Med Genet. 2006; 43 (8): 665-70.

26. Umans-Eckenhausen MA, Defesche JC, Sijbrands EJ, Scheerder RL, Kastelein JJ. Review of first 5 years of screening for familial hypercholesterolaemia in the Netherlands. Lancet. 2001; 357 (9251): 165-8.

27. Mitchell C, Ploem MC, Hennekam RC, Kaye J. A Duty To Warn Relatives in Clinical Genetics: Arguably 'Fair just and reasonable' in English Law? Tottels J Prof Neglig. 2016; 32 (2): 120-36.

28. Weaver M. The Double Helix: Applying an Ethic of Care to the Duty to Warn Genetic Relatives of Genetic Information. Bioethics. 2016; 30 (3): 181-7. DOI: 10.1111/bioe.12176.

29. Wouters RH, Bijlsma RM, Ausems MG, van Delden JJ, Voest EE, Bredenoord AL. Am I My Family's Keeper? Disclosure Dilemmas in Next-Generation Sequencing. Hum Mutat. 2016; 37 (12): 1257-62. DOI: 10.1002/humu.23118.

30. Leonard SJ, Newson, A. J. Ethical perspectives. In: Gaff CL, Bylund, C.L. Family communication about genetics: Theory and practice. New York: Oxford University Press, inc.; 2010.

For peer review only - http://bmjopen.bmj.com/site/about/guidelines.xhtml

31. Chan AW, Tetzlaff JM, Altman DG, et al. SPIRIT 2013 Statement: defining standard protocol items for clinical trials. Rev Panam Salud Publica. 2015; 38(6): 506-14. DOI: 10.7326/0003-4819-158-3-201302050-00583

32. Castor BV. Castor Electronic Data Capture. Amsterdam 2016.

33. Eijzenga W, de Geus E, Aalfs CM, et al. How to support cancer genetics counselees in informing at-risk relatives? Lessons from a randomized controlled trial. Patient Education and Counseling. 2018. 101 (9):1611-1619. DOI: 10.1016/j.pec.2018.05.009

34. Mesters I, van den Borne H, McCormick L, Pruyn J, de Boer M, Imbos T. Openness to discuss cancer in the nuclear family: scale, development, and validation. Psychosom Med. 1997; 59 (3): 269-79.

35. Custers JA, van den Berg SW, van Laarhoven HW, Bleiker EM, Gielissen MF, Prins JB. The Cancer Worry Scale: detecting fear of recurrence in breast cancer survivors. Cancer Nurs. 2014; 37 (1): E44-50. DOI: 10.1097/NCC.0b013e3182813a17.

36. Spinhoven P, Ormel J, Sloekers PP, Kempen GI, Speckens AE, Van Hemert AM. A validation study of the Hospital Anxiety and Depression Scale (HADS) in different groups of Dutch subjects. Psychol Med. 1997; 27 (2): 363-70.

37. Ong LM, Visser MR, van Zuuren FJ, Rietbroek RC, Lammes FB, de Haes JC. Cancer patients' coping styles and doctor-patient communication. Psychooncology. 1999; 8 (2): 155-66.

38. Van Zuuren FJ, De Groot, K.I., Mulder, N.L., Muris, P. Coping with medical threat: An evaluation of the Threatening Medical Situations Inventory (TMSI). Personality and Individual Differences. 1996; 21 (1): 21-31. DOI: 10.1016/0191-8869(96)00029-3.

39. Wakefield CE, Homewood J, Mahmut M, Taylor A, Meiser B. Usefulness of the Threatening Medical Situations Inventory in individuals considering genetic testing for cancer risk. Patient Educ Couns. 2007; 69 (1-3): 29-38.

For peer review only - http://bmjopen.bmj.com/site/about/guidelines.xhtml

40. Spielberger CD. Assessment of state and trait anxiety: Conceptual and methodological issues. South Psychol. 1985 (2): 6-16.

41. Van der Ploeg HM & Spielberger CD. Handleiding bij de Zelf-Beoordelings Vragenlijst ZBV. Een Nederlandse bewerking van de Spielberger State-Trait Anxiety Inventory STAI-DY. Lisse: Swets & Zeitlinger; 1981.

42. De Geus E, Aalfs CM, Menko FH, et al. Development of the Informing Relatives Inventory (IRI): Assessing Index Patients' Knowledge, Motivation and Self-Efficacy Regarding the Disclosure of Hereditary Cancer Risk Information to Relatives. Int J Behav Med. 2015; 22 (4): 551-60. DOI: 10.1007/s12529-014-9455-x.

43. Van der Vaart R, Drossaert CH, Taal E, et al. Validation of the Dutch functional, communicative and critical health literacy scales. Patient Educ Couns. 2012; 89 (1): 82-8. DOI: 10.1016/j.pec.2012.07.014.

44. SPSS Inc. IBM SPSS Statistics for Windows. Armonk (NY): IBM; 2016.

45. Braun V, Clarke, V. Using thematic analysis in psychology. Qual Res Psychol. 2006; 3 (2): 77-101. DOI: 10.1191/1478088706qp063oa.

46. VERBI Software. MAXQDA Analytics Pro 12 ed. Berlin, Germany: VERBI; 2016.

Authors contributions

LvdH and IC designed the intervention. LvdH, IC, ES and PvT were involved in conception and design of the protocol. LvdH was involved in drafting the manuscript. IC, ES, PvT, YH and AB critically revised the manuscript. All authors were involved in the final approval of the manuscript.

Funding statement

For peer review only - http://bmjopen.bmj.com/site/about/guidelines.xhtml

We acknowledge the financial support from the Netherlands Cardiovascular Research Initiative: An initiative with support of the Dutch Heart Foundation, CVON2015-12 eDETECT and CVON2017-10 DOLPHIN-GENESIS.

Competing interests statement

All authors declare they have no competing interests.

Acknowledgements

Patient advisors are acknowledged for their input regarding the design of this randomised controlled trial.

Figures

Figure 1 Flow-chart of the study procedure

For peer review only

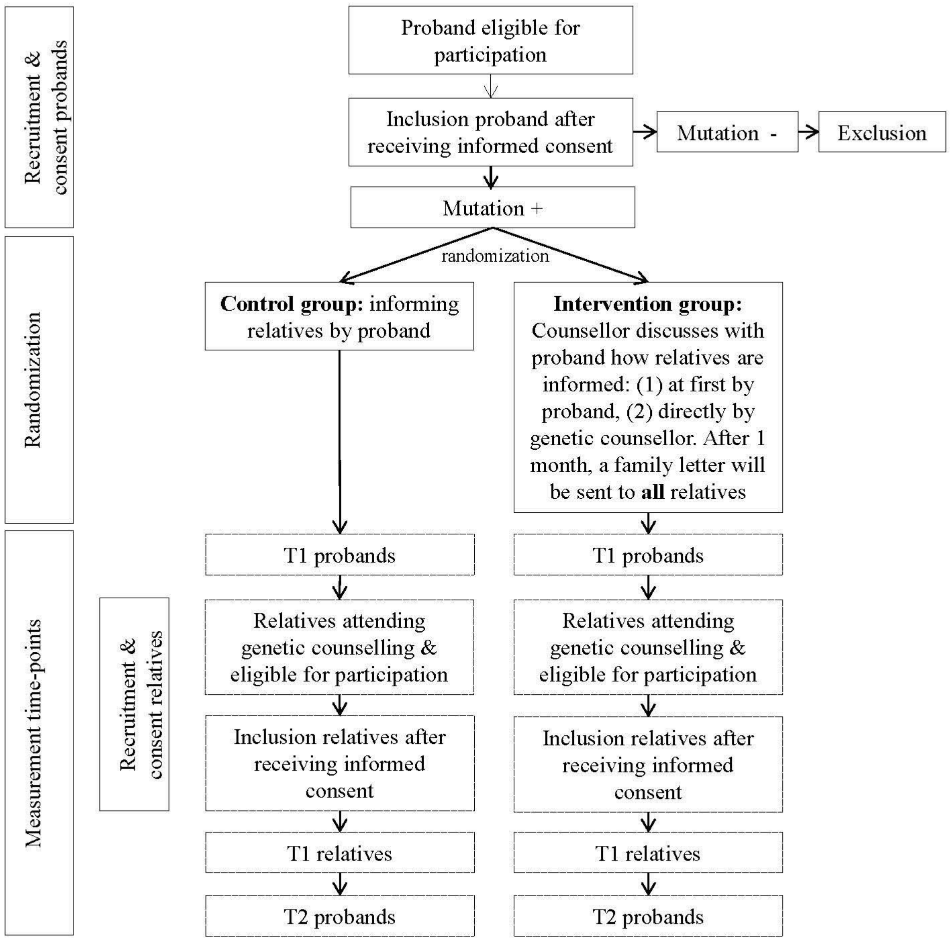

Figure 1 Study procedure

126x130mm (300 x 300 DPI)

Supplementary Material S1

Supplementary material S1: Information letter and informed consent form probands and relatives

Dear Sir or Madam,

Recently you visited the department of Clinical Genetics for genetic counselling concerning a possible inherited cardiac disease in your family. A clinical geneticist or genetic counsellor informed you about participation in a research study examining how to inform relatives at risk of inheriting the genetic predisposition. In this letter, we would like to inform you about this research study.

What is the goal of this research study?
At the current moment, we ask people in whom a genetic cause for a cardiac disease is identified to inform their relatives about the possibility of genetic testing, supported by a letter written by the clinician. This research study aims to investigate whether another approach to informing relatives would be more effective. Using this approach, people who are the first in their family in whom the hereditary predisposition is identified can decide which relatives they prefer to inform themselves and which relatives they prefer to have contacted directly by the clinician. In addition, a website will be used to further inform relatives. This research study will investigate which approach is the most effective.

Who is conducting the study?
This research study will be conducted by the department of Clinical Genetics and the department of Medical Psychology of the Amsterdam UMC, in collaboration with the department of Clinical Genetics of the University Medical Centre Groningen (UMCG) and the University Medical Centre Utrecht (UMCU). The study is funded by the Dutch Heart Foundation.

What does study participation involve?
If the DNA test shows that no genetic variant causing the disease is identified, no further activity is needed for this study. If the DNA test shows that a genetic variant causing the disease is identified, the clinician will inform you about advice for your relatives. To investigate what the most effective approach is, one group of participants will be asked to inform relatives using the approach that is currently used, while the other group of participants will be asked to inform relatives using the other approach. Which group you are part of will be determined randomly.

For peer review only

Supplementary Material S1

In addition, we will ask you to fill out two questionnaires. These questionnaires can be administered online or by mail. If you prefer to receive the questionnaire by mail, the questionnaire can be returned using the provided return envelope. These questionnaires ask you for some general information, such as your age and gender, and about your experiences with informing relatives and your opinion regarding the approach that was used. You will receive the first questionnaire two months after you receive the DNA test result. The second questionnaire will be sent to you after nine months.

What are advantages and disadvantages of study participation?
There will be no direct benefit to you for your participation in this study. We hope that the information obtained from this study may contribute to research on improving approaches to informing relatives at risk of inherited cardiac diseases. Participation in this research study will take time: we will ask you to fill out two questionnaires. We expect that this will cost you 20 to 30 minutes per questionnaire. In addition, we will ask questions regarding personal information, such as the disease in your family and your opinion and feelings concerning informing your relatives about this disease. You will not receive an incentive for study participation.

What if I do not want to participate anymore?
Your participation in this research is entirely voluntary. There are no consequences if you decide not to participate. You may also change your mind later and stop participating during the study even if you earlier agreed to do so. If you decide to stop participation, please let us know. The data collected up to that moment will be used for the research study. The researcher will inform you if there is new information about the study that might be important for you. In that case, you will be asked if you still consent to participate in the study.

How will my personal data be handled?
By participating in this study, you will provide us personal data. This data will be collected for the research study. A research code will be assigned to all research documents. This means that your name and other personal data are not visible on research documents. Only the researchers know which research code belongs to you. Some institutes (the Safety Committee and the Health Care Inspectorate) are allowed to look into your clinical and personal data. These institutes are allowed to do this to control whether the research study is conducted in a proper and reliable manner. They will treat your data confidentially. If you sign the informed consent form, you will provide consent for collection, storage and inspection of your medical and personal data. Your data will be stored for 15 years.

Do you have any further questions regarding this research study?
If you have any questions about this research study or about study participation, please contact the executing researcher Lieke van den Heuvel by telephone (<telephone number>) or e-mail (<e-mail address>). If you prefer to discuss study participation with an independent clinician, or if you would like additional information, advice or support, please contact the independent expert (see attachment).

What if I want to participate?

For peer review only

Supplementary Material S1

The researcher, Lieke van den Heuvel, will contact you by telephone to provide further information about the research study. If you decide to participate in this study after this telephone contact, a consent form will be sent to you by mail. You can return this consent form by using the attached return envelope. A postage stamp is not necessary.

Thank you for your time!

Sincerely,

Lieke van den Heuvel, MSc

Also on behalf of the research team and all the clinical geneticists and genetic counsellors involved from Amsterdam UMC, University Medical Centre Utrecht and University Medical Centre Groningen

Attachment: Information about your hospital 'UMCG/Amsterdam UMC/UMCU'

Questions, suggestions or complaints
If you have any questions, suggestions or a complaint about this research study, please contact Lieke van den Heuvel. She will conduct this research study. She can be contacted by telephone (<telephone number>) or by e-mail (<e-mail address>).

Independent advice or support
If you prefer to discuss study participation with someone who is not involved in this research study, because you need further information or advice, you may contact <independent researcher> (<e-mail address>). He/she is a clinical geneticist at the department of Clinical Genetics at UMCG/Amsterdam UMC/UMCU.

For peer review only

Supplementary Material S1

Certificate of consent
For participation in a research study regarding informing relatives at risk of an inherited cardiac disease

- I am satisfied about the information I received about this research study. I have had sufficient time to consider participation in this study. I have had the opportunity to ask questions about it and any questions that I have asked have been answered to my satisfaction.
- I can stop study participation at any time, without providing any reason and without any consequences.

By signing this form, I provide consent for:

1. Informing my clinician (clinical geneticist/genetic counselor) about my participation in this research study.
2. Collecting of data using questionnaire(s), and inspection of my medical record until 12 months after signing this consent form and the use of these data for the research study described in this letter.
3. Being approached for other research projects in the future.
4. Storing of my research data for 15 years after the research study has been finished
5. Inspection of the research data by the Safety Committee and the Dutch Health Care Inspectorate

I want to participate in this research study

Name of participant: ____

Date: ____/____/___

Signature of participant:____

I would like to be informed about the group results of this research study:

- ☐ Yes
- ☐ No

Statement by the researcher: I have accurately informed this person about the aforementioned research project. The person may stop participating at any time during the study without any consequences. Any questions from the person about study participation were answered sufficiently

Name of researcher:____

Date: ____/____/____

Signature of researcher: ____

For peer review only

Supplementary Material S1

Dear Sir or Madam,

Recently you visited the department of Clinical Genetics for genetic counselling concerning an inherited cardiac disease in your family. A clinical geneticist or genetic counsellor informed you about participation in a research study examining how to inform relatives at risk of inheriting the genetic predisposition. In this letter, we would like to inform you about this research study.

What is the goal of this research study?
At the current moment, we ask people in whom a genetic cause for a cardiac disease is identified to inform their relatives about the possibility of genetic testing, supported by a letter written by the clinician. This research study aims to investigate whether another approach to informing relatives would be more effective. Using this approach, people who are the first in their family in whom the hereditary predisposition is identified can decide which relatives they prefer to inform themselves and which relatives they prefer to have contacted directly by the clinician. In addition, a website will be used to further inform relatives. This research study will investigate which approach is the most effective.

Who is conducting the study?
This research study will be conducted by the Department of Clinical Genetics and the Department of Medical Psychology of the Amsterdam UMC, in collaboration with the Departments of Clinical Genetics of the University Medical Centre Groningen (UMCG) and the University Medical Centre Utrecht (UMCU). The study is funded by the Dutch Heart Foundation.

What does study participation involve?
For this research study, we ask you to fill out a questionnaire. This questionnaire can be administered online or by mail. If you prefer to receive the questionnaire by mail, the questionnaire can be returned by using the provided return envelope. These questionnaires ask you for some general information, such as your age and gender, and about your experiences with being informed about the inherited cardiac disease in your family and your opinion regarding the approach that was used. This questionnaire will also ask you some questions about how you feel, at the current moment and in general, and how you generally cope with complex situations.

In addition, it is important for this research study to receive some information about your medical background. Because of this, we ask you to provide consent to inspect your medical record up to 12 months after completion of this research study. Important information includes, for example, the inherited cardiac disease diagnosed in your family and information

For peer review only

Supplementary Material S1

about your family history regarding cardiac diseases. This information will be handled confidentially.

Your clinician (the clinical geneticist/genetic counsellor) will be informed if you decide to participate in this study. You might be approached for other research studies in the future.

What are advantages and disadvantages of study participation?
There will be no direct benefit to you for your participation in this study. We hope that the information obtained from this study will contribute to research on improving approaches to inform relatives at risk of inherited cardiac diseases. Participation in this research study will take time: we will ask you to fill out two questionnaires. We expect that this will cost you 20 to 30 minutes per questionnaire. In addition, we will ask questions regarding personal information, such as the disease in your family and your opinion and feelings concerning informing your relatives about this disease. You will not receive an incentive for study participation.

What if I do not want to participate anymore?
Your participation in this research is entirely voluntary. There are no consequences if you decide not to participate. You may also change your mind later and stop participating during the study even if you earlier agreed to do so. If you decide to stop participation, please let us know. The data collected up to that moment will be used for the research study. The researcher will inform you if there is new information about the study that might be important for you. In that case, you will be asked if you still consent to participate in the study.

How will my personal data be handled?
By participating in this study, you will provide us personal data. This data will be collected for the research study. A research code will be assigned to all research documents. This means that your name and other personal data are not visible on research documents. Only the researchers know which research code belongs to you. Some institutes (the Safety Committee and the Health Care Inspectorate) are allowed to look into your clinical and personal data. These institutes are allowed to do this to control whether the research study is conducted in a proper and reliable manner. They will treat your data confidentially. If you sign the informed consent form, you will provide consent for collection, storage and inspection of your medical and personal data. Your data will be stored for 15 years.

Do you have any further questions regarding this research study?
If you have any questions about this research study or about study participation, please contact the executing researcher Lieke van den Heuvel by telephone (<telephone number>) or e-mail (<e-mail address>). If you prefer to discuss study participation with an independent clinician, or if you would like additional information, advice or support, please contact the independent expert (see attachment).

What if I want to participate?
The researcher, Lieke van den Heuvel, will contact you by telephone to provide further information about the research study. If you decide to participate in this study after this telephone contact, a consent form will be sent to you by mail. You can return this consent

For peer review only

Supplementary Material S1

form using the attached return envelope. A postage stamp is not necessary. Subsequently, you will receive the questionnaire online or by mail.

Thank you for your time!

Sincerely,

Lieke van den Heuvel, MSc

Also on behalf of the research team and all the clinical geneticists and genetic counsellors involved from Amsterdam UMC, University Medical Centre Utrecht and University Medical Centre Groningen

Attachment: Information about your hospital ‘UMCG/Amsterdam UMC/UMCU’

Questions, suggestions or complaints
If you have any questions, suggestions or a complaint about this research study, please contact Lieke van den Heuvel. She will conduct this research study. She can be contacted by telephone (<telephone number>) or by e-mail (<e-mail address>).

Independent advice or support
If you prefer to discuss study participation with someone who is not involved in this research study, because you need further information or advice, you may contact <independent researcher> (<e-mail address>). He/she is a clinical geneticist at the department of Clinical Genetics at UMCG/Amsterdam UMC/UMCU.

For peer review only

Supplementary Material S1

Certificate of consent
For participation in a research study regarding informing relatives at risk of an inherited cardiac disease

– I am satisfied about the information I received about this research study. I have had sufficient time to consider participation in this study. I have had the opportunity to ask questions about it, and any questions that I have asked have been answered to my satisfaction.
– I can stop study participation at any time, without providing any reason and without any consequences.

By signing this form, I provide consent for:

6. Informing my clinician (clinical geneticist/genetic counselor) about my participation in this research study.
7. Collection of data using questionnaire(s), and inspection of my medical record until 12 months after signing this consent form and the use of these data for the research study described in this letter.
8. Being approached for other research projects in the future.
9. Storage of research data for 15 years after the research study has been finished
10. Inspection of the research data by the Safety Committee and the Dutch Health Care Inspectorate

I want to participate in this research study

Name of participant: ____

Date: ____/____/___

Signature of participant:____

I would like to be informed about the group results of this research study:

- ☐ Yes
- ☐ No

Statement by the researcher: I have accurately informed this person about the aforementioned research project. The person may stop participating at any time during the study without any consequences. Any questions from the person about study participation were answered sufficiently

Name of researcher:____

Date: ____/____/____

Signature of researcher: ____

For peer review only

Supplementary Material S2

Supplementary material S2: Overview of questionnaire items to assess secondary outcomes per time-point

Table 1 Questionnaire items probands per time-point

Questionnaire time-point	Items	Questionnaires
T1	Sociodemographic, clinical, family and personality factors	Self-constructed items<br>Trait scale of STAI<br>Shortened version of TMSI
	Advices for relatives at risk, eligible relatives at risk, the number of informed relatives at risk, risk perception and experiences with informing relatives at risk	Eleven self-constructed items
	Evaluation of used approach (incl. website)	Thirteen self-constructed items
	Perceived impact on family communication with relatives at risk	Adapted version of ODCF<br>One self-constructed item
	Impact on psychological functioning of proband	HADS<br><br>Adapted version of CWS
T2	Number of informed relatives at risk, risk perception and experiences with informing relatives at risk	Three self-constructed items
	Evaluation of approach used	One self-constructed item
	Perceived impact on family communication with relatives at risk	Adapted version of ODCF<br>One self-constructed item
	Impact on psychological functioning of proband	HADS<br>Adapted version of CWS

Table 2 Questionnaire items relatives

Questionnaire time-point	Items	Questionnaires
T1	Sociodemographic, family and personality factors	Eight self-constructed items<br>Trait scale of STAI<br>Shortened version of TMSI
	Evaluation of used approach (incl. website), risk perception	Thirteen self-constructed items
	Perceived impact on family communication with index patient	Adapted version of ODCF<br>One self-constructed item
	Impact on psychological functioning of family member	HADS<br>Adapted version of CWS

Table 3 Self-constructed items (telephone interview) - Experiences with informing relatives at risk (probands)

1. Did the genetic counsellor give you an advice for your relatives?<br>2. What was this advice? (open question)<br>3. For which relatives was this advice meant? (open question)	Yes/No

For peer review only - http://bmjopen.bmj.com/site/about/guidelines.xhtml

Supplementary Material S2

4. Have relatives at risk been informed about the advice of the genetic counsellor? If yes, which relatives have been informed?<br>5. Who informed your relatives?	Yes/No

Table 4 Self-constructed items (telephone interview) - Risk perception (probands)

1. How do you consider the risk of your relatives on being a carrier of the familial variant?<br>2. How do you estimate the risk of your relatives on developing symptoms of the ICC?	0% - 100%<br>1-10<br>0% - 100%<br>1-10

Table 5 Self-constructed items - Evaluation of the used approach (probands)

T1	*Closed questions*<br>*Below you can see statements regarding your experiences with how your relatives have been informed. Please rate each statement on a scale of 1-5 (1 totally disagree to 5 totally agree) how much each statement applies to you.*<br>1. I feel supported by the genetic counsellor in informing my relatives<br>2. I think the used approach to inform relatives at risk is acceptable<br>3. I felt a little coerced to inform my relatives<br>4. The way my relatives are informed, can be improved<br>5. I am satisfied with the way my relatives are informed<br>Other:________________________________	1 2 3 4 5<br>1 2 3 4 5<br>1 2 3 4 5<br>1 2 3 4 5<br>1 2 3 4 5
T2	1. Did your opinion regarding the used approach change?<br>a. Yes, my opinion regarding the used approach became more positive<br>b. Yes, my opinion regarding the used approach became more negative<br>c. No, my opinion regarding the used approach is still positive<br>d. No, my opinion regarding the used approach is still negative<br>e. No, my opinion regarding the used approach is still neutral	
T1/T2	1. Do you think another approach to inform relatives at risk would have been better?<br>2. Are there relatives for which you would have preferred another approach to inform them?	Yes/No<br>Yes/No
T1/T2	*Open questions*<br>1. What are advantages of the approach used to inform your relatives?<br>2. What are disadvantages of the approach used to inform your relatives?	

Table 6 Self-constructed items - Impact on family relationships (probands)

1. Are there relatives with whom your relationship has changed after they are informed about their risk on the inherited cardiac disease?

For peer review only

Supplementary Material S2

a. Yes, our relationship improved<br>b. Yes, our relationship worsened<br>c. No, our relationship is still not good/not bad<br>d. No, our relationship is still good<br>e. No, our relationship is still bad

Table 7 Self-constructed items - Evaluation of the used approach (relatives)

Closed questions<br>*Below you can see statements regarding your experiences with how you have been informed about the inherited cardiac disease in your family. Please rate each statement on a scale of 1-5 (1 totally disagree to 5 totally agree) how much each statement applies to you.*	
1. I appreciated to be informed about my risk on the inherited cardiac disease	1 2 3 4 5
2. I am satisfied with the way I have been informed	1 2 3 4 5
3. I preferred to have received more information before I contacted the clinical genetic centre	1 2 3 4 5
4. I understand why I have been informed	1 2 3 4 5
5. The way I have been informed, can be improved	1 2 3 4 5
6. I felt free to decide myself whether I wanted to contact the clinic genetic centre	1 2 3 4 5
7. I would have preferred not to be informed about my risk on the inherited cardiac disease in my family	1 2 3 4 5
8. I would have preferred to not know about the inherited cardiac disease in my family	1 2 3 4 5
Other:________________________________	
--	
1. Do you think another approach to be informed would have been better?	Yes/No
--	
Open questions<br>1. What are advantages of the way you have been informed?<br>2. What are disadvantages of the way you have been informed?	

Table 8 Self-constructed items - Impact on family relationships (relatives)

1. Did your relationship with your relative change after they were informed about their risk on the inherited cardiac disease?<br>a. Yes, our relationship improved<br>b. Yes, our relationship worsened<br>c. No, our relationship is still not good/not bad<br>d. No, our relationship is still good<br>e. No, our relationship is still bad

For peer review only

SPIRIT

STANDARD PROTOCOL ITEMS: RECOMMENDATIONS FOR INTERVENTIONAL TRIALS

SPIRIT 2013 Checklist: Recommended items to address in a clinical trial protocol and related documents*

Section/item	Item No	Description	Page no
**Administrative information**			
Title	1	Descriptive title identifying the study design, population, interventions, and, if applicable, trial acronym	1
Trial registration	2a	Trial identifier and registry name. If not yet registered, name of intended registry	2
	2b	All items from the World Health Organization Trial Registration Data Set	6-17
Protocol version	3	Date and version identifier	-
Funding	4	Sources and types of financial, material, and other support	18
Roles and responsibilities	5a	Names, affiliations, and roles of protocol contributors	1
	5b	Name and contact information for the trial sponsor	-
	5c	Role of study sponsor and funders, if any, in study design; collection, management, analysis, and interpretation of data; writing of the report; and the decision to submit the report for publication, including whether they will have ultimate authority over any of these activities	-
	5d	Composition, roles, and responsibilities of the coordinating centre, steering committee, endpoint adjudication committee, data management team, and other individuals or groups overseeing the trial, if applicable (see Item 21a for data monitoring committee)	-
**Introduction**			
Background and rationale	6a	Description of research question and justification for undertaking the trial, including summary of relevant studies (published and unpublished) examining benefits and harms for each intervention	4-6
	6b	Explanation for choice of comparators	4-6
Objectives	7	Specific objectives or hypotheses	6

For peer review only

Trial design	8	Description of trial design including type of trial (eg, parallel group, crossover, factorial, single group), allocation ratio, and framework (eg, superiority, equivalence, noninferiority, exploratory)	6
**Methods: Participants, interventions, and outcomes**			
Study setting	9	Description of study settings (eg, community clinic, academic hospital) and list of countries where data will be collected. Reference to where list of study sites can be obtained	6
Eligibility criteria	10	Inclusion and exclusion criteria for participants. If applicable, eligibility criteria for study centres and individuals who will perform the interventions (eg, surgeons, psychotherapists)	7
Interventions	11a	Interventions for each group with sufficient detail to allow replication, including how and when they will be administered	9, 10
	11b	Criteria for discontinuing or modifying allocated interventions for a given trial participant (eg, drug dose change in response to harms, participant request, or improving/worsening disease)	17
	11c	Strategies to improve adherence to intervention protocols, and any procedures for monitoring adherence (eg, drug tablet return, laboratory tests)	-
	11d	Relevant concomitant care and interventions that are permitted or prohibited during the trial	-
Outcomes	12	Primary, secondary, and other outcomes, including the specific measurement variable (eg, systolic blood pressure), analysis metric (eg, change from baseline, final value, time to event), method of aggregation (eg, median, proportion), and time point for each outcome. Explanation of the clinical relevance of chosen efficacy and harm outcomes is strongly recommended	11-14
Participant timeline	13	Time schedule of enrolment, interventions (including any run-ins and washouts), assessments, and visits for participants. A schematic diagram is highly recommended (see Figure)	8, 11, 17
Sample size	14	Estimated number of participants needed to achieve study objectives and how it was determined, including clinical and statistical assumptions supporting any sample size calculations	15, 16
Recruitment	15	Strategies for achieving adequate participant enrolment to reach target sample size	-
**Methods: Assignment of interventions (for controlled trials)**			
Allocation:			

Sequence generation	16a	Method of generating the allocation sequence (eg, computer-generated random numbers), and list of any factors for stratification. To reduce predictability of a random sequence, details of any planned restriction (eg, blocking) should be provided in a separate document that is unavailable to those who enrol participants or assign interventions	9
Allocation concealment mechanism	16b	Mechanism of implementing the allocation sequence (eg, central telephone; sequentially numbered, opaque, sealed envelopes), describing any steps to conceal the sequence until interventions are assigned	9
Implementation	16c	Who will generate the allocation sequence, who will enrol participants, and who will assign participants to interventions	9
Blinding (masking)	17a	Who will be blinded after assignment to interventions (eg, trial participants, care providers, outcome assessors, data analysts), and how	9
	17b	If blinded, circumstances under which unblinding is permissible, and procedure for revealing a participant's allocated intervention during the trial	9
**Methods: Data collection, management, and analysis**			
Data collection methods	18a	Plans for assessment and collection of outcome, baseline, and other trial data, including any related processes to promote data quality (eg, duplicate measurements, training of assessors) and a description of study instruments (eg, questionnaires, laboratory tests) along with their reliability and validity, if known. Reference to where data collection forms can be found, if not in the protocol	11-15
	18b	Plans to promote participant retention and complete follow-up, including list of any outcome data to be collected for participants who discontinue or deviate from intervention protocols	-
Data management	19	Plans for data entry, coding, security, and storage, including any related processes to promote data quality (eg, double data entry; range checks for data values). Reference to where details of data management procedures can be found, if not in the protocol	17
Statistical methods	20a	Statistical methods for analysing primary and secondary outcomes. Reference to where other details of the statistical analysis plan can be found, if not in the protocol	16, 17
	20b	Methods for any additional analyses (eg, subgroup and adjusted analyses)	16, 17
	20c	Definition of analysis population relating to protocol non-adherence (eg, as randomised analysis), and any statistical methods to handle missing data (eg, multiple imputation)	16, 17

**Methods: Monitoring**			
Data monitoring	21a	Composition of data monitoring committee (DMC); summary of its role and reporting structure; statement of whether it is independent from the sponsor and competing interests; and reference to where further details about its charter can be found, if not in the protocol. Alternatively, an explanation of why a DMC is not needed	NA
	21b	Description of any interim analyses and stopping guidelines, including who will have access to these interim results and make the final decision to terminate the trial	17
Harms	22	Plans for collecting, assessing, reporting, and managing solicited and spontaneously reported adverse events and other unintended effects of trial interventions or trial conduct	-
Auditing	23	Frequency and procedures for auditing trial conduct, if any, and whether the process will be independent from investigators and the sponsor	NA
**Ethics and dissemination**			
Research ethics approval	24	Plans for seeking research ethics committee/institutional review board (REC/IRB) approval	17
Protocol amendments	25	Plans for communicating important protocol modifications (eg, changes to eligibility criteria, outcomes, analyses) to relevant parties (eg, investigators, REC/IRBs, trial participants, trial registries, journals, regulators)	-
Consent or assent	26a	Who will obtain informed consent or assent from potential trial participants or authorised surrogates, and how (see Item 32)	8, 9
	26b	Additional consent provisions for collection and use of participant data and biological specimens in ancillary studies, if applicable	NA
Confidentiality	27	How personal information about potential and enrolled participants will be collected, shared, and maintained in order to protect confidentiality before, during, and after the trial	17
Declaration of interests	28	Financial and other competing interests for principal investigators for the overall trial and each study site	18
Access to data	29	Statement of who will have access to the final trial dataset, and disclosure of contractual agreements that limit such access for investigators	17
Ancillary and post-trial care	30	Provisions, if any, for ancillary and post-trial care, and for compensation to those who suffer harm from trial participation	NA

For peer review only

Dissemination policy	31a	Plans for investigators and sponsor to communicate trial results to participants, healthcare professionals, the public, and other relevant groups (eg, via publication, reporting in results databases, or other data sharing arrangements), including any publication restrictions	17
	31b	Authorship eligibility guidelines and any intended use of professional writers	18
	31c	Plans, if any, for granting public access to the full protocol, participant-level dataset, and statistical code	-
**Appendices**			
Informed consent materials	32	Model consent form and other related documentation given to participants and authorised surrogates	-
Biological specimens	33	Plans for collection, laboratory evaluation, and storage of biological specimens for genetic or molecular analysis in the current trial and for future use in ancillary studies, if applicable	NA

*It is strongly recommended that this checklist be read in conjunction with the SPIRIT 2013 Explanation & Elaboration for important clarification on the items. Amendments to the protocol should be tracked and dated. The SPIRIT checklist is copyrighted by the SPIRIT Group under the Creative Commons "Attribution-NonCommercial-NoDerivs 3.0 Unported" license.

For peer review only

BMJ Open

A tailored approach towards informing relatives at risk of inherited cardiac conditions: study protocol for a randomised controlled trial

Journal:	*BMJ Open*
Manuscript ID	bmjopen-2018-025660.R2
Article Type:	Protocol
Date Submitted by the Author:	14-Feb-2019
Complete List of Authors:	van den Heuvel, Lieke; Amsterdam University Medical Centres, Department of Clinical Genetics<br>Hoedemaekers, Yvonne; University Medical Centre Groningen, Department of Clinical Genetics<br>Baas, Annette; University Medical Center Utrecht, Department of Genetics<br>van Tintelen, J; Amsterdam University Medical Centres, Department of Clinical Genetics<br>Smets, Ellen; Amsterdam University Medical Centres, Department of Medical Psychology<br>Christiaans, Imke; Amsterdam University Medical Centres, Clinical Genetics
**Primary Subject Heading**:	Genetics and genomics
Secondary Subject Heading:	Communication, Ethics, Genetics and genomics, Cardiovascular medicine, Health policy
Keywords:	Inherited cardiac conditions, Cardiogenetics, Informing relatives at risk, Family-mediated approach, Tailored approach, Randomized controlled trial

SCHOLARONE™
Manuscripts

A tailored approach towards informing relatives at risk of inherited cardiac conditions: study protocol for a randomised controlled trial

L.M. van den Heuvel[1], Y.M. Hoedemaekers[2], A.F. Baas[3], J.P. van Tintelen[1], E.M.A. Smets[4]& I. Christiaans[1*]

[1]Department of Clinical Genetics, Amsterdam University Medical Centres / University of Amsterdam, Amsterdam, the Netherlands

[2]Department of Genetics, University of Groningen, University Medical Centre Groningen, Groningen, the Netherlands

[3]Department of Genetics, University Medical Centre Utrecht / University Utrecht, Utrecht, the Netherlands

[4]Department of Medical Psychology, Amsterdam University Medical Centres / University of Amsterdam, Amsterdam, the Netherlands

* Corresponding author

Department of Clinical Genetics,

Amsterdam University Medical Centre, location AMC

Meibergdreef 9, 1105 AZ, Amsterdam, the Netherlands

Telephone: +31 20-5667217

E-mail: i.christiaans@amc.nl

For peer review only

For peer review only - http://bmjopen.bmj.com/site/about/guidelines.xhtml

Abstract

Introduction In current practice, probands are asked to inform relatives about the possibility of predictive DNA testing when a pathogenic variant causing an inherited cardiac condition (ICC) is identified. Previous research on the uptake of genetic counselling and predictive DNA testing in relatives suggests that not all relatives are sufficiently informed. We developed a randomised controlled trial to evaluate the effectiveness of a tailored approach in which probands decide together with the genetic counsellor which relatives they inform themselves and which relatives they prefer to have informed by the genetic counsellor. Here, we present the study protocol of this randomised controlled trial. **Methods** A multicentre randomised controlled trial with parallel-group design will be conducted in which an intervention group receiving the tailored approach will be compared to a control group receiving usual care. Adult probands diagnosed with an ICC in whom a likely pathogenic or pathogenic variant is identified, will be randomly assigned to the intervention or control group (total sample: n = 85 probands). Primary outcomes are uptake of genetic counselling and predictive DNA testing by relatives (total sample: n = 340 relatives). Secondary outcomes are appreciation of the approach used and impact on familial and psychological functioning, which will be assessed using questionnaires. Relatives who attend genetic counselling will be asked to fill out a questionnaire as well. **Ethics and dissemination** Ethical approval was obtained from the Medical Ethical Committee of the Amsterdam University Medical Centres (MEC 2017-145), the Netherlands. All participants will provide informed consent prior to participation in the study. Results of the study on primary and secondary outcome measures will be published in peer-reviewed journals. **Registration details** This trial is registered at the Netherlands Trial Register NTR6657.

Key words Inherited cardiac conditions, cardiogenetics, informing relatives at risk, family-mediated approach, tailored approach, randomised controlled trial

For peer review only - http://bmjopen.bmj.com/site/about/guidelines.xhtml

Strengths and limitations of this study

- This randomised controlled trial investigates both the uptake of genetic counselling and of predictive DNA testing, as well as the acceptance and impact on psychological and family functioning in the tailored versus the standard approach, in probands and relatives.
- This study will be conducted in three clinical genetics clinics with expertise on cardiogenetics, which will facilitate participant inclusion.
- In this trial, evaluation of the effect on outcome of the different components of the intervention is not possible, due to limited power.
- In this randomised controlled trial it is not possible to blind participants, genetic counsellors or the executing investigator for the chosen intervention.
- Because a baseline measure for the secondary outcomes is not possible, we cannot control for likely confounding factors, such as intention to inform at-risk relatives, and family and psychological functioning at baseline.

For peer review only - http://bmjopen.bmj.com/site/about/guidelines.xhtml

Introduction

Inherited cardiac conditions (ICCs) such as cardiomyopathies and primary arrhythmia syndromes generally demonstrate an autosomal dominant inheritance pattern and a wide variety of symptoms that can manifest at any age [1, 2]. One feared outcome is sudden cardiac death (SCD), which can occur at a young age and be the first symptom of disease [3, 4]. With an incomplete penetrance and high variability in expression even within families, carriers of a familial variant may remain undetected but still be at risk for SCD even though treatment options are available that prevent disease progression or potentially life-threatening arrhythmias [5]. Predictive DNA testing is therefore offered to first-degree relatives of probands (the first person in a family diagnosed with an ICC) in whom a pathogenic variant is identified because these relatives are at 50% risk of also having inheriting the genetic variant [5, 6]. Predictive DNA testing is offered to relatives in a stepwise manner (cascade screening), with the aim of identifying asymptomatic carriers of the familial variant to facilitate timely treatment. Non-carriers of the familial variant generally do not need cardiac monitoring and can be reassured about their own risk and that of their offspring [6].

In current practice in the Netherlands, probands are asked to inform their relatives, supported by a family letter written by the genetic counsellor. This is referred to as the family-mediated approach [7]. Previous research, however, shows that uptake (the number of relatives at risk attending genetic counselling and/or undergoing predictive DNA testing) is relatively low in ICCs, particularly for cardiomyopathies. Reported uptakes are around 50% despite family letters being provided to a majority of relatives by the proband [8-10]. Previous research in other genetic patient populations, such as hereditary types of cancer, shows similar uptake percentages [11-13].

Some relatives who do not attend genetic counselling will have deliberately decided against predictive DNA testing. However, the low uptake percentages also suggest that many

For peer review only - http://bmjopen.bmj.com/site/about/guidelines.xhtml

relatives may be unaware, or insufficiently aware, of the risks involved and/or the possibilities for genetic counselling and subsequent surveillance and treatment. This is supported by research on family communication in ICCs. Patients are not always able to inform or correctly inform their relatives for a number of reasons, including disengagement with relatives, lack of understanding of the importance of the information, preoccupation with their own grief, difficulties in conveying the complex information to relatives, or a wish to prevent burdening relatives by informing them about genetic risks [8, 14-18].

Previous studies assessing interventions to enhance family communication in hereditary diseases showed that some interventions are effective in increasing the uptake of genetic counselling [19-21]. An intervention trial aimed at improving family communication in specifically dilated cardiomyopathy is still ongoing [22]. A few studies have been published on more active approaches to informing relatives at risk in which healthcare professionals (HCPs) contact at-risk relatives directly [23-26]. These studies suggest that a more active approach can almost double the uptake of genetic counselling and predictive DNA testing by relatives (23-26) However, some of these studies were performed in a research setting (e.g. in relatives already registered in research databases for the genetic disease), hampering direct translation of these results to a diagnostic setting. To our knowledge, more active approaches in patients with ICCs have not been studied thus far. However, a study by Ormondroyd et al [14] suggests that relatives eligible for predictive DNA testing for hypertrophic cardiomyopathy and long QT syndrome would support a more active approach to informing relatives at risk.

Although studies on more active approaches did not report any psychological harm in relatives at group level, these approaches could cause more unwarranted worry or pressure on relatives to opt for predictive DNA testing [23-25]. An active approach to informing relatives at risk could also breach the autonomy and confidentiality of probands, and may harm

For peer review only - http://bmjopen.bmj.com/site/about/guidelines.xhtml

relative's right not to know [27-29]. Furthermore, HCPs are often unaware of interpersonal dynamics within families and the personal circumstances of relatives at risk. Active approaches may therefore have a negative impact on family relationships or may cause psychological distress in both probands and relatives [30].

Because of this, a tailored approach in which a proband decides together with the genetic counsellor which at-risk relatives he or she will inform and which relatives he or she prefers to be informed by the genetic counsellor could be optimal. With this approach, the probands expert knowledge of a relative's functioning and of family dynamics could be used appropriately, and the autonomy of the proband preserved. At the same time, more relatives at risk would be sufficiently informed [28, 30]. Furthermore, probands for whom informing relatives is difficult or burdensome might be relieved or supported by this approach [30].

Objectives

The primary aim of this randomised controlled trial is to assess whether uptake of genetic counselling and testing of relatives at risk of an ICC will be increased by using a tailored approach to information provision for relatives, instead of usual care (i.e. the family-mediated approach). Secondary objectives are to evaluate how such a tailored approach is appreciated by both probands and relatives as compared to usual care. In addition, this study aims to assess the perceived impact on family relationships and psychological functioning of both probands and relatives. The protocol presented here has been described based on the SPIRIT statement [31]

Methods

Design

A multicentre randomised controlled trial with a parallel-group design will be conducted in three university hospitals in the Netherlands (the Amsterdam University Medical Centres

For peer review only - http://bmjopen.bmj.com/site/about/guidelines.xhtml

(Amsterdam UMC), the University Medical Centre Utrecht (UMCU) and the University Medical Centre Groningen (UMCG)) to compare the effects of a tailored approach to informing relatives at risk of ICCs to usual care in both probands and relatives.

Participants

All probands aged 18 years or older with an ICC, or suspicion thereof, attending pre-test genetic counselling at the cardiogenetics outpatient clinics during the inclusion period will be asked to participate if they: (1) are the first member of their family to visit the cardiogenetics outpatient clinic for counselling about genetic testing for ICCs; (2) they have at least one living adult relative; and (3) are able to read and write Dutch. Only probands in whom a likely pathogenic or pathogenic variant is detected (class 4 - likely pathogenic or class 5 - pathogenic variant) will be definitively included.

In addition, eligible adult first- (or second-) degree relatives of enrolled probands who make an appointment at the cardiogenetics outpatient clinics will be invited to fill out a questionnaire to measure secondary outcomes. Inclusion criteria are defined as follows: (1) first-degree adult (18 years or older) relatives of probands enrolled in the study or second-degree adult relatives in case of a deceased connecting first-degree relative who was affected or suspected to be affected, and (2) able to read and write Dutch.

Procedure

Figure 1 shows a flowchart of the study procedure.

Recruitment & consent

During pre-test genetic counselling, the genetic counsellor will inform the probands about the study and provide an informational letter (see Supplementary Material S1). In addition, probands will be asked if the executing researcher can contact them to provide further

For peer review only

For peer review only - http://bmjopen.bmj.com/site/about/guidelines.xhtml

information about the study. Subsequently, probands will be contacted by telephone by the executing researcher. If probands are still interested in participation, written informed consent forms will be sent by post, including a return envelope. As described above, only probands in whom a likely pathogenic or pathogenic variant is detected will be definitively included in the study.

Relatives of enrolled probands attending pre-test genetic counselling in one of the participating centres who are also at risk will also be invited to participate in the study. The same recruitment procedure will be used.

Randomisation

Prior to receiving their test result, probands with an ICC in whom a likely pathogenic or pathogenic variant is identified will be randomly assigned to either the intervention or control group. Block randomisation will be used, with variable blocks ranging from size two to six. Randomisation will be stratified for gender, disease type (cardiomyopathies or primary arrhythmia syndromes) and hospital. To ensure allocation concealment, computer software will be used for randomisation, with an allocation rate of 1:1 [32]. Relatives of probands included in the study will be assigned to the group to which the proband was assigned.

Neither participants nor genetic counsellors will or can be blinded for group assignment. The executing researcher also cannot be blinded because of slight differences between the questionnaires administered in the intervention- and control groups. Part of the outcome data will be collected using telephone interviews. To minimize bias, these interviews will be conducted by a research assistant following a structured script.

Intervention group

For peer review only - http://bmjopen.bmj.com/site/about/guidelines.xhtml

In the intervention group, a tailored approach to informing relatives at risk will be provided. In this approach, probands with a likely pathogenic or pathogenic variant will discuss with the genetic counsellor which relatives are at risk of inheriting the familial variant. They will then be asked which of these relatives they prefer to inform themselves at first using a family letter written by the genetic counsellor, and which relatives they prefer to be directly informed by the genetic counsellor with a similar family letter. This will be discussed during routine post-test counselling. In both cases, after one month, the genetic counsellor will send the family letter directly to all relatives at risk for whom the proband has provided consent to contact. The proband will be asked to provide contact details of these relatives.

The family letter is standardised for all three participating centres. For the intervention group, the letter also includes a link to a website specifically designed for this study where relatives can find additional information (www.familieleden.erfelijkehartziekten.nl). The information on this website will be tailored to relative's situations (i.e. specified for disease-type, hospital, parenthood, whether relatives have a desire to have children in the future, and which information relatives prefer to receive) by asking them to fill out a short questionnaire on their first visit to the website.

<u>Control group</u>

In the control group, the standard care approach will be used. If a likely pathogenic or pathogenic variant is identified, probands assigned to the control group will be asked by the genetic counsellor to inform relatives at risk about the genetic test result, the consequences of this result for relatives and the advice regarding predictive DNA testing and/or cardiac monitoring. This will be discussed during routine post-test counselling. Probands will be supported in informing relatives at risk by a family letter written by the genetic counsellor. This family letter is also standardised for all three participating centres. However, this letter

For peer review only - http://bmjopen.bmj.com/site/about/guidelines.xhtml

does not include the link to the website with tailored information described above, but does include a link to a general website on ICCs (www.erfelijkehartziekten.nl).

Measurement time-points

For secondary outcome measures, participating probands will be asked to complete a questionnaire one month after receiving the genetic test result (T1) and to complete a second questionnaire nine months after the test result (T2). Before T1 and T2, a short structured telephone interview will be conducted about participant's knowledge of which relatives are at risk of ICCs and which relatives are informed, because these items are expected to be too complex to answer in a questionnaire [33]. Participating relatives will complete one questionnaire after attending genetic counselling.

Measures

Primary outcome measures

To assess the effect of a tailored approach to informing relatives at risk, the difference between the intervention and control groups in uptake of (1) genetic counselling, and (2) predictive DNA testing of relatives at risk will be measured. To do this, the number of relatives attending genetic counselling and the number of relatives who are genetically tested in the first year after detection of the likely pathogenic or pathogenic variant in the proband will be collected in the laboratories of each participating centre. DNA test results of relatives counselled in non-participating centres will also be taken into account because, in the Netherlands, predictive DNA testing of relatives is always performed in the same laboratory where the proband was tested.

The number of relatives attending genetic counselling and undergoing predictive DNA testing will be compared to the total number of relatives at risk of inheriting the variant who

For peer review only - http://bmjopen.bmj.com/site/about/guidelines.xhtml

are eligible for genetic counselling and predictive DNA testing based on family pedigrees. For relatives who attend genetic counselling but decide against predictive DNA testing, subsequent attendance of cardiac monitoring will be checked.

Relatives at risk who are eligible for genetic counselling and predictive DNA testing are first-degree relatives and second-degree relatives if there is a connecting deceased first-degree relative suspected of having an ICC. Following the Dutch clinical guidelines for cardiomyopathies, relatives at risk are eligible for genetic counselling and predictive DNA testing from the age of 10 years. For primary arrhythmias, depending on the specific arrhythmic disorder, relatives at risk are eligible for predictive DNA testing from birth.

Furthermore, conditional uptake of relatives at risk, defined as the number of relatives who are genetically tested relative to the number who attend genetic counselling, will be calculated. Uptake will be measured at randomisation condition (intervention or control group) and family level.

Secondary outcome measures

Secondary outcome measures will be measured using both validated and self-constructed questionnaire items. An overview of these items is shown in the Supplementary Material S2. Secondary outcome measures include the following:

- Appreciation of the information provision strategy used and preferences regarding the approach used to inform relatives at risk: This will be evaluated in both probands and relatives using self-constructed items on a 5 point Likert scale (1 = ‘Totally disagree’ to 5 = ‘Totally agree’) in a questionnaire (probands: 5 items, range 5-25; relatives: 6 items, range 6-30). Probands will be asked to answer an additional self-constructed item during the structured telephone interview about whether they would have preferred to inform their relatives differently. Two additional self-constructed items will be administered in

For peer review only - http://bmjopen.bmj.com/site/about/guidelines.xhtml

the intervention group to assess decisional conflict in probands, including whether probands thought it was difficult to choose to inform their relatives themselves or have them informed by the counsellor, and whether they were satisfied by their decision, on a 5 point Likert scale (1 = 'Totally disagree' to 5 = 'Totally agree'; range 2-10). Probands will be asked to fill out these items at T1. At T2, a self-constructed item will be administered to assess whether their opinion regarding the approach used has changed. The questionnaire for relatives also includes a self-constructed item on how they were informed (i.e., by whom they were informed and what information was provided). Finally, probands (at T1 and T2) and relatives will be asked whether they visited the website www.erfelijkehartziekten.nl and, if yes, how they evaluated the website, using four self-constructed items on a 5 point Likert scale (1 = 'Totally disagree' to 5 = 'Totally agree'; range 4-20).

- Impact on family communication: To assess the impact of the tailored approach versus the usual care approach on family functioning, probands (at T1 and T2) and relatives will be asked to fill out an adapted version of the Openness to Discuss Cancer in the Family (ODCF) scale, which assesses communication about genetic risks within families with nine items on a 5 point Likert scale (1 = 'Totally disagree' to 5 = 'Totally agree'; range 9-45) [34]. Psychometric characteristics of the original ODCF scale are satisfactory [34]. In addition, a self-constructed item will be administered asking about the nature of regular communication with relatives and whether probands and relatives experienced changes in their relationships with relatives as a consequence of the information provision process.
- Impact on psychological functioning: To assess the impact on psychological functioning, two validated questionnaires will be administered in probands (at T1 and T2) and relatives. Participants will be asked to fill out an adapted version of the Cancer Worry Scale (CWS) [35]. The CWS was developed and validated in Dutch patients with

For peer review only - http://bmjopen.bmj.com/site/about/guidelines.xhtml

hereditary types of cancer [35]. Because it was validated in a Dutch patient population and is previously used in a genetic patient population, it was considered the most appropriate scale for this randomised controlled trial. The CWS consists of eight items on a 4 point Likert scale (1 = 'Almost never' to 4 = 'Almost always'; range 8-32). Psychometric characteristics of the CWS have been assessed in a sample of breast cancer survivors, and support its reliability and validity [35].

In addition, the Hospital Anxiety and Depression Scale (HADS) will be administered to assess whether participants experience anxious or depressed feelings after being informed about the hereditary disease [36]. The HADS contains two 7-item subscales on a 4 point Likert scale with diverse answer options that assess both anxiety and depression with a score range of 0-21. Psychometric characteristics of the HADS were assessed as good [36].

Participants' characteristics

To assess whether randomisation succeeded and whether characteristics of participating probands and relatives have influenced the primary and secondary outcome measures, sociodemographic and clinical factors will be collected, including gender, education level, ethnicity, living situation and parenthood, family history and the diagnosis of the probands at T1. Relatives will additionally be asked what their degree of kinship is with the proband.

For the same reason, psychosocial and personality factors will be assessed in both probands (at T1) and relatives. Coping style will be assessed by using the shortened version of the Threatening Medical Situations Inventory (TMSI) [37, 38]. The TMSI assesses a "monitoring" versus "blunting" coping style related to a medical threat, and it was previously evaluated in an oncogenetic patient population [37, 39]. The shortened version of the TMSI contains two subscales, both consisting of six items on a 5 point Likert scale (1 = 'Totally not

For peer review only - http://bmjopen.bmj.com/site/about/guidelines.xhtml

applicable' to 5 = 'Totally applicable'; range 6-30). Reliability and validity are satisfactory [37, 38]. The Trait subscale of the State Trait Anxiety Inventory (STAI) will be administered to assess trait anxiety in both probands and relatives [40]. The STAI is frequently used in research settings and consists of 20 items on a 4 point Likert scale (1 = 'Not at all' to 4 = 'Very much so'; range 20-80) [40]. The reliability and validity for the Dutch translation of the STAI are assessed as good [41].

Self-efficacy and perceived motivators and barriers regarding informing relatives at risk will be assessed using an adapted version of the 'motivation' and 'self-efficacy' subscales of the Informing Relatives Inventory (IRI) [42]. The IRI was developed and evaluated in an oncogenetic patient population, and showed satisfactory reliability and validity [42]. The 'motivation' subscale consists of 30 items on a 5 point Likert scale (1 = 'No role' to 5 = 'A large role'; range 30-150); the 'self-efficacy' subscale of 7 items on a 4 point Likert scale (1 = 'Not sure at all' to 4 = 'Very sure'; range 7-21). Probands will also be asked to answer a self-constructed item during the telephone interviews regarding whether relatives were informed and whether probands intended to inform (remaining) at-risk relatives.

Risk perception regarding the risk of relatives carrying the variant and developing the disease will be assessed by using self-constructed items. These items ask participants to rate the perception of the risk of relatives carrying the variant and developing the disease on a scale from 1 (lowest risk) to 10 (highest risk) as well as from 0% (lowest risk) to 100% (highest risk).

Health literacy – defined as the ability to obtain, process and understand basic health information and services – will be assessed in probands and relatives using the items on the 'functional health literacy' and 'communicative health literacy' subscales of the 3HL questionnaire [43]. Both subscales contain five items on a 4 point Likert scale (1 = 'Never' to

For peer review only - http://bmjopen.bmj.com/site/about/guidelines.xhtml

4 = ‘Often’; range per subscale 5-20). The reliability for both scales was assessed as high and the validity as satisfactory [43].

Sample size calculation

The study aims to detect a difference of 15% in uptake of genetic counselling by relatives between the control (usual care, 50%) and intervention groups (tailored approach, 65%). Assuming a two-sided 5% significance level and a power of 80%, 340 relatives (170 in each group) would be required to participate in this study. On average, six relatives per proband are at 50% risk of inheriting the variant, including children and adults [9]. With a conservative estimate of four eligible adult relatives per proband at risk, 85 probands with an ICC and an identified likely pathogenic or pathogenic (class 4 or 5) variant will need to be included in this study to reach 340 relatives. A likely pathogenic or pathogenic variant is found in, on average, 20% (lower margin) of all probands with a suspected ICC. With an expected response rate of 70% and a drop-out rate of 20%, approximately 759 probands will be approached to participate in the study.

Data analysis

Statistical analysis

Sociodemographic, clinical, psychosocial and personality variables will be analysed using descriptive and frequency statistics. An intention-to-treat approach will be used. SPSS version 24.0 will be used to perform statistical analyses [44]. An α level of $p < .05$ will be used. Analysis of Variance and chi-square tests will be used to assess differences (i) in sociodemographic, clinical and psychological characteristics between the intervention- and control group and (ii) in participants and non-participants, as appropriate. Descriptive and frequency statistics will be used to describe the primary outcomes: (1) uptake of genetic

For peer review only - http://bmjopen.bmj.com/site/about/guidelines.xhtml

counselling and (2) uptake of predictive DNA testing. Logistic regression analysis will be conducted to assess differences between the intervention- and control group on the primary outcomes, with the randomisation group as the main exploratory variable. Two logistic regression models will be used, with the first model including only the randomisation group and the second model also including the potential covariates (i.e., sociodemographic, clinical and psychological variables). Multilevel analyses will be performed to assess whether the randomisation group, i.e., the independent variable, has an impact on family and psychological functioning, i.e., the secondary outcome variables. The two measurement time-points in probands will be treated as nested within probands. To prevent influence of potential confounding factors, multilevel analysis will be adjusted for covariates as well. Participant appreciation of the approach used will be described using frequency statistics.

Qualitative analysis

Open questions will be analysed using thematic analysis based on the principles of Braun and Clarke [45]. Analysis software for qualitative data, MAXQDA version 12, will be used [46]. Two trained coders will conduct the coding analysis of open answer options independently. Codes will be discussed and modified by the two coders until agreement is met. Subsequently, the coders will analyse and interpret the codes to create a structure of main themes and subthemes. The qualitative results will be used to supplement the questionnaire data.

Patient and public involvement

Prior to this randomised controlled trial, face-to-face interviews were conducted with probands and counselled relatives (both carriers and non-carriers) to explore their experiences with and preferences regarding informing at-risk relatives (unpublished). In addition, online focus groups were conducted with HCPs. The randomised controlled trial was then designed

For peer review only - http://bmjopen.bmj.com/site/about/guidelines.xhtml

based on the findings of both these interviews and focus groups. Since this study is part of the eDETECT research consortium (CVON2015-12), several patient representative groups (the PLN foundation; Harteraad, Heartz) participated in the user committee and scientific meetings and thereby gave input to this research proposal. Patients are not involved in the recruitment and conduct of the study.

During patient seminars, patients will be updated on the progress and results of the study. In addition, during the eDETECT scientific meetings, all participants of the eDETECT consortium (including representatives of the aforementioned patient organisations) will be informed. After completion of the study, group results will be disseminated by e-mail to study participants who indicated their interest in the outcome during informed consent. A summary of the results will also be posted on the ICCs website (www.erfelijkehartziekten.nl).

The burden of the intervention was not assessed because this is an intrinsic part of the outcome measures of this study. The patients themselves were involved in pilot testing the questionnaires used to assess these outcome measures.

Ethics and dissemination

The Medical Ethical Committee of the Amsterdam UMC has approved the study design (MEC 2017-145). Additional approval of regional Medical Ethical Committees of the other participating academic centres has also been obtained. Informed consent is required from each participant. Participants who provide written informed consent can withdraw from the study at any time, without providing a reason.

After receiving informed consent, a unique research ID will be assigned to the participant. Only this ID will be used to identify research documents. Each research document will be saved on a secured server. The principal investigator, coordinating investigator and

For peer review only - http://bmjopen.bmj.com/site/about/guidelines.xhtml

executing investigator have access to this secured server. Research documents will be saved for a period of 15 years. This randomised controlled trial is registered at the Netherlands Trial Register NTR6657. Separate manuscripts with findings on, respectively, the primary and secondary outcomes will be published in peer-reviewed journals.

Trial status

Recruitment of probands during pre-test genetic counselling for this randomised controlled trial started in November 2017. In total, recruitment of probands will last one year. Subsequent uptake of genetic counselling and predictive DNA testing will be measured until one year after the detection of a pathogenic variant in the proband. Data collection will therefore continue until January 2020, taking into account a duration of, on average, three months for the DNA-test result in the proband to be available. To date, 68 probands have been included and randomised to either the intervention or the control group. In addition, 49 relatives consented to participate.

References

1. Theilade J, Kanters J, Henriksen FL, et al. Cascade screening in families with inherited cardiac diseases driven by cardiologists: feasibility and nationwide outcome in long QT syndrome. Cardiology. 2013; 126 (2): 131-7. DOI: 10.1159/000350825.

2. Watkins H, Ashrafian H, Redwood C. Inherited cardiomyopathies. N Engl J Med. 2011; 364 (17): 1643-56. DOI: 10.1056/NEJMra0902923.

3. Behr ER, Dalageorgou C, Christiansen M, et al. Sudden arrhythmic death syndrome: familial evaluation identifies inheritable heart disease in the majority of families. Eur Heart J. 2008; 29 (13): 1670-80. DOI: 10.1093/eurheartj/ehn219.

For peer review only - http://bmjopen.bmj.com/site/about/guidelines.xhtml

4. Van der Werf C, Hofman N, Tan HL, et al. Diagnostic yield in sudden unexplained death and aborted cardiac arrest in the young: the experience of a tertiary referral center in The Netherlands. Heart Rhythm. 2010; 7 (10): 1383-9. DOI: 10.1016/j.hrthm.2010.05.036.

5. Wilde AA, Behr ER. Genetic testing for inherited cardiac disease. Nat Rev Cardiol. 2013; 10 (10): 571-83. DOI: 10.1038/nrcardio.2013.108.

6. Miller EM, Wang Y, Ware SM. Uptake of cardiac screening and genetic testing among hypertrophic and dilated cardiomyopathy families. J Genet Couns. 2013; 22 (2) :258-67. DOI: 10.1007/s10897-012-9544-4.

7. Leenen CH, Heijer M, van der Meer C, Kuipers EJ, van Leerdam ME, Wagner A. Genetic testing for Lynch syndrome: family communication and motivation. Fam Cancer. 2016; 15 (1): 63-73. DOI: 10.1007/s10689-015-9842-8.

8. Burns C, McGaughran J, Davis A, Semsarian C, Ingles J. Factors influencing uptake of familial long QT syndrome genetic testing. Am J Med Genet A. 2016; 170A (2): DOI: 10.1002/ajmg.a.37455.

9. Christiaans I, Birnie E, Bonsel GJ, Wilde AA, van Langen IM. Uptake of genetic counselling and predictive DNA testing in hypertrophic cardiomyopathy. Eur J Hum Genet. 2008; 16 (10): 1201-7. DOI: 10.1038/ejhg.2008.92.

10. Van der Roest WP, Pennings JM, Bakker M, van den Berg MP, van Tintelen JP. Family letters are an effective way to inform relatives about inherited cardiac disease. Am J Med Genet A. 2009; 149A (3): 357-63. DOI: 10.1002/ajmg.a.32672.

11. Brooks L, Lennard F, Shenton A, et al. BRCA1/2 predictive testing: a study of uptake in two centres. Eur J Hum Genet. 2004; 12 (8): 654-62.

12. Menko FH, Ter Stege JA, van der Kolk LE, et al. The uptake of presymptomatic genetic testing in hereditary breast-ovarian cancer and Lynch syndrome: a systematic review of the

For peer review only - http://bmjopen.bmj.com/site/about/guidelines.xhtml

literature and implications for clinical practice. Fam Cancer. 2018. DOI: 10.1007/s10689-018-0089-z.

13. Ramsoekh D, van Leerdam ME, Tops CM, et al. The use of genetic testing in hereditary colorectal cancer syndromes: genetic testing in HNPCC, (A)FAP and MAP. Clin Genet. 2007; 72(6): 562-7.

14. Ormondroyd E, Oates S, Parker M, Blair E, Watkins H. Pre-symptomatic genetic testing for inherited cardiac conditions: a qualitative exploration of psychosocial and ethical implications. Eur J Hum Genet. 2014; 22 (1): 88-93. DOI: 10.1038/ejhg.2013.81.

15. Smart A. Impediments to DNA testing and cascade screening for hypertrophic cardiomyopathy and Long QT syndrome: a qualitative study of patient experiences. J Genet Couns. 2010; 19 (6): 630-9. DOI: 10.1007/s10897-010-9314-0.

16. Vavolizza RD, Kalia I, Erskine Aaron K, et al. Disclosing Genetic Information to Family Members About Inherited Cardiac Arrhythmias: An Obligation or a Choice? J Genet Couns. 2015; 24 (4): 608-15. DOI: 10.1007/s10897-014-9783-7.

17. Whyte S, Green A, McAllister M, Shipman H. Family Communication in Inherited Cardiovascular Conditions in Ireland. J Genet Couns. 2016; 25 (6): 1317-26. DOI: 10.1007/s10897-016-9974-5.

18. Batte B, Sheldon JP, Arscott P, et al. Family communication in a population at risk for hypertrophic cardiomyopathy. J Genet Couns. 2015; 24 (2): 336-48. DOI: 10.1007/s10897-014-9774-8.

19. Forrest LE, Burke J, Bacic S, Amor DJ. Increased genetic counseling support improves communication of genetic information in families. Genet Med. 2008; 10 (3): 167-72. DOI: 10.1097/GIM.0b013e318164540b.

For peer review only - http://bmjopen.bmj.com/site/about/guidelines.xhtml

20. Hodgson JM, Metcalfe SA, Aitken M, et al. Improving family communication after a new genetic diagnosis: a randomised controlled trial of a genetic counselling intervention. BMC Med Genet. 2014; 15: 33. DOI: 10.1186/1471-2350-15-33.

21. Hodgson JM, Metcalfe S, Gaff C, et al. Outcomes of a randomised controlled trial of a complex genetic counselling intervention to improve family communication. Eur J Hum Genet. 2016; 24 (3): 356-60. DOI: 10.1038/ejhg.2015.122.

22. Kinnamon DD, Morales A, Bowen DJ, Burke W, Hershberger RE, Consortium* DCM. Toward Genetics-Driven Early Intervention in Dilated Cardiomyopathy: Design and Implementation of the DCM Precision Medicine Study. Circ Cardiovasc Genet. 2017; 10 (6). DOI: 10.1161/CIRCGENETICS.117.001826.

23. Aktan-Collan K, Haukkala A, Pylvanainen K, Jarvinen HJ, Aaltonen LA, Peltomaki P, et al. Direct contact in inviting high-risk members of hereditary colon cancer families to genetic counselling and DNA testing. J Med Genet. 2007; 44 (11): 732-8.

24. Sermijn E, Delesie L, Deschepper E, et al. The impact of an interventional counselling procedure in families with a BRCA1/2 gene mutation: efficacy and safety. Fam Cancer. 2016; 15 (2): 155-62. DOI: 10.1007/s10689-015-9854-4.

25. Suthers GK, Armstrong J, McCormack J, Trott D. Letting the family know: balancing ethics and effectiveness when notifying relatives about genetic testing for a familial disorder. J Med Genet. 2006; 43 (8): 665-70.

26. Umans-Eckenhausen MA, Defesche JC, Sijbrands EJ, Scheerder RL, Kastelein JJ. Review of first 5 years of screening for familial hypercholesterolaemia in the Netherlands. Lancet. 2001; 357 (9251): 165-8.

27. Mitchell C, Ploem MC, Hennekam RC, Kaye J. A Duty To Warn Relatives in Clinical Genetics: Arguably 'Fair just and reasonable' in English Law? Tottels J Prof Neglig. 2016; 32 (2): 120-36.

For peer review only - http://bmjopen.bmj.com/site/about/guidelines.xhtml

28. Weaver M. The Double Helix: Applying an Ethic of Care to the Duty to Warn Genetic Relatives of Genetic Information. Bioethics. 2016; 30 (3): 181-7. DOI: 10.1111/bioe.12176.

29. Wouters RH, Bijlsma RM, Ausems MG, van Delden JJ, Voest EE, Bredenoord AL. Am I My Family's Keeper? Disclosure Dilemmas in Next-Generation Sequencing. Hum Mutat. 2016; 37 (12): 1257-62. DOI: 10.1002/humu.23118.

30. Leonard SJ, Newson, A. J. Ethical perspectives. In: Gaff CL, Bylund, C.L. Family communication about genetics: Theory and practice. New York: Oxford University Press, inc.; 2010.

31. Chan AW, Tetzlaff JM, Altman DG, et al. SPIRIT 2013 Statement: defining standard protocol items for clinical trials. Rev Panam Salud Publica. 2015; 38(6): 506-14. DOI: 10.7326/0003-4819-158-3-201302050-00583

32. Castor BV. Castor Electronic Data Capture. Amsterdam 2016.

33. Eijzenga W, de Geus E, Aalfs CM, et al. How to support cancer genetics counselees in informing at-risk relatives? Lessons from a randomized controlled trial. Patient Education and Counseling. 2018. 101 (9):1611-1619. DOI: 10.1016/j.pec.2018.05.009

34. Mesters I, van den Borne H, McCormick L, Pruyn J, de Boer M, Imbos T. Openness to discuss cancer in the nuclear family: scale, development, and validation. Psychosom Med. 1997; 59 (3): 269-79.

35. Custers JA, van den Berg SW, van Laarhoven HW, Bleiker EM, Gielissen MF, Prins JB. The Cancer Worry Scale: detecting fear of recurrence in breast cancer survivors. Cancer Nurs. 2014; 37 (1): E44-50. DOI: 10.1097/NCC.0b013e3182813a17.

36. Spinhoven P, Ormel J, Sloekers PP, Kempen GI, Speckens AE, Van Hemert AM. A validation study of the Hospital Anxiety and Depression Scale (HADS) in different groups of Dutch subjects. Psychol Med. 1997; 27 (2): 363-70.

For peer review only - http://bmjopen.bmj.com/site/about/guidelines.xhtml

37. Ong LM, Visser MR, van Zuuren FJ, Rietbroek RC, Lammes FB, de Haes JC. Cancer patients' coping styles and doctor-patient communication. Psychooncology. 1999; 8 (2): 155-66.

38. Van Zuuren FJ, De Groot, K.I., Mulder, N.L., Muris, P. Coping with medical threat: An evaluation of the Threatening Medical Situations Inventory (TMSI). Personality and Individual Differences. 1996; 21 (1): 21-31. DOI: 10.1016/0191-8869(96)00029-3.

39. Wakefield CE, Homewood J, Mahmut M, Taylor A, Meiser B. Usefulness of the Threatening Medical Situations Inventory in individuals considering genetic testing for cancer risk. Patient Educ Couns. 2007; 69 (1-3): 29-38.

40. Spielberger CD. Assessment of state and trait anxiety: Conceptual and methodological issues. South Psychol. 1985 (2): 6-16.

41. Van der Ploeg HM & Spielberger CD. Handleiding bij de Zelf-Beoordelings Vragenlijst ZBV. Een Nederlandse bewerking van de Spielberger State-Trait Anxiety Inventory STAI-DY. Lisse: Swets & Zeitlinger; 1981.

42. De Geus E, Aalfs CM, Menko FH, et al. Development of the Informing Relatives Inventory (IRI): Assessing Index Patients' Knowledge, Motivation and Self-Efficacy Regarding the Disclosure of Hereditary Cancer Risk Information to Relatives. Int J Behav Med. 2015; 22 (4): 551-60. DOI: 10.1007/s12529-014-9455-x.

43. Van der Vaart R, Drossaert CH, Taal E, et al. Validation of the Dutch functional, communicative and critical health literacy scales. Patient Educ Couns. 2012; 89 (1): 82-8. DOI: 10.1016/j.pec.2012.07.014.

44. SPSS Inc. IBM SPSS Statistics for Windows. Armonk (NY): IBM; 2016.

45. Braun V, Clarke, V. Using thematic analysis in psychology. Qual Res Psychol. 2006; 3 (2): 77-101. DOI: 10.1191/1478088706qp063oa.

46. VERBI Software. MAXQDA Analytics Pro 12 ed. Berlin, Germany: VERBI; 2016.

For peer review only - http://bmjopen.bmj.com/site/about/guidelines.xhtml

Authors contributions

LvdH and IC designed the intervention. LvdH, IC, ES and PvT were involved in conception and design of the protocol. LvdH was involved in drafting the manuscript. IC, ES, PvT, YH and AB critically revised the manuscript. All authors were involved in the final approval of the manuscript.

Funding statement

We acknowledge the financial support from the Netherlands Cardiovascular Research Initiative: An initiative with support of the Dutch Heart Foundation, CVON2015-12 eDETECT and CVON2017-10 DOLPHIN-GENESIS.

Competing interests statement

All authors declare they have no competing interests.

Acknowledgements

Patient advisors are acknowledged for their input regarding the design of this randomised controlled trial.

Figures

Figure 1 Flow-chart of the study procedure

For peer review only - http://bmjopen.bmj.com/site/about/guidelines.xhtml

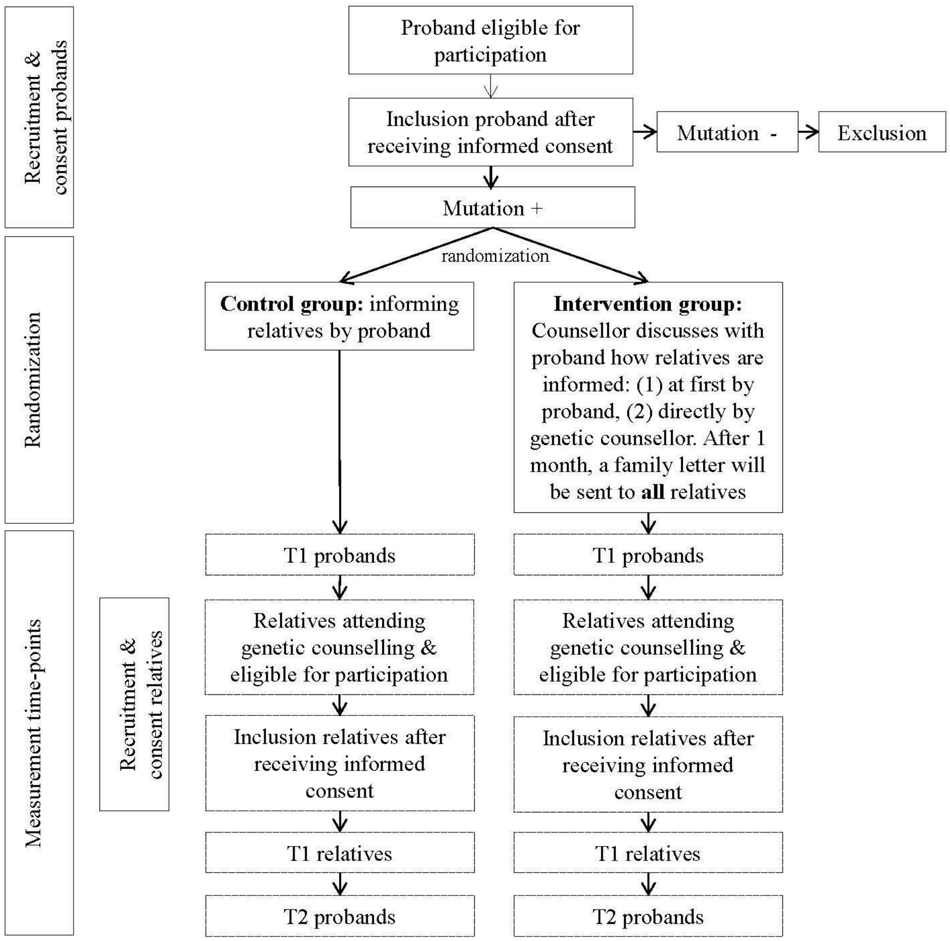

Figure 1 Study procedure

126x130mm (300 x 300 DPI)

Supplementary Material S1

Supplementary material S1: Information letter and informed consent form probands and relatives

Dear Sir or Madam,

Recently you visited the department of Clinical Genetics for genetic counselling concerning a possible inherited cardiac disease in your family. A clinical geneticist or genetic counsellor informed you about participation in a research study examining how to inform relatives at risk of inheriting the genetic predisposition. In this letter, we would like to inform you about this research study.

What is the goal of this research study?
At the current moment, we ask people in whom a genetic cause for a cardiac disease is identified to inform their relatives about the possibility of genetic testing, supported by a letter written by the clinician. This research study aims to investigate whether another approach to informing relatives would be more effective. Using this approach, people who are the first in their family in whom the hereditary predisposition is identified can decide which relatives they prefer to inform themselves and which relatives they prefer to have contacted directly by the clinician. In addition, a website will be used to further inform relatives. This research study will investigate which approach is the most effective.

Who is conducting the study?
This research study will be conducted by the department of Clinical Genetics and the department of Medical Psychology of the Amsterdam UMC, in collaboration with the department of Clinical Genetics of the University Medical Centre Groningen (UMCG) and the University Medical Centre Utrecht (UMCU). The study is funded by the Dutch Heart Foundation.

What does study participation involve?
If the DNA test shows that no genetic variant causing the disease is identified, no further activity is needed for this study. If the DNA test shows that a genetic variant causing the disease is identified, the clinician will inform you about advice for your relatives. To investigate what the most effective approach is, one group of participants will be asked to inform relatives using the approach that is currently used, while the other group of participants will be asked to inform relatives using the other approach. Which group you are part of will be determined randomly.

For peer review only

Supplementary Material S1

In addition, we will ask you to fill out two questionnaires. These questionnaires can be administered online or by mail. If you prefer to receive the questionnaire by mail, the questionnaire can be returned using the provided return envelope. These questionnaires ask you for some general information, such as your age and gender, and about your experiences with informing relatives and your opinion regarding the approach that was used. You will receive the first questionnaire two months after you receive the DNA test result. The second questionnaire will be sent to you after nine months.

What are advantages and disadvantages of study participation?
There will be no direct benefit to you for your participation in this study. We hope that the information obtained from this study may contribute to research on improving approaches to informing relatives at risk of inherited cardiac diseases. Participation in this research study will take time: we will ask you to fill out two questionnaires. We expect that this will cost you 20 to 30 minutes per questionnaire. In addition, we will ask questions regarding personal information, such as the disease in your family and your opinion and feelings concerning informing your relatives about this disease. You will not receive an incentive for study participation.

What if I do not want to participate anymore?
Your participation in this research is entirely voluntary. There are no consequences if you decide not to participate. You may also change your mind later and stop participating during the study even if you earlier agreed to do so. If you decide to stop participation, please let us know. The data collected up to that moment will be used for the research study. The researcher will inform you if there is new information about the study that might be important for you. In that case, you will be asked if you still consent to participate in the study.

How will my personal data be handled?
By participating in this study, you will provide us personal data. This data will be collected for the research study. A research code will be assigned to all research documents. This means that your name and other personal data are not visible on research documents. Only the researchers know which research code belongs to you. Some institutes (the Safety Committee and the Health Care Inspectorate) are allowed to look into your clinical and personal data. These institutes are allowed to do this to control whether the research study is conducted in a proper and reliable manner. They will treat your data confidentially. If you sign the informed consent form, you will provide consent for collection, storage and inspection of your medical and personal data. Your data will be stored for 15 years.

Do you have any further questions regarding this research study?
If you have any questions about this research study or about study participation, please contact the executing researcher Lieke van den Heuvel by telephone (<telephone number>) or e-mail (<e-mail address>). If you prefer to discuss study participation with an independent clinician, or if you would like additional information, advice or support, please contact the independent expert (see attachment).

What if I want to participate?

For peer review only

Supplementary Material S1

The researcher, Lieke van den Heuvel, will contact you by telephone to provide further information about the research study. If you decide to participate in this study after this telephone contact, a consent form will be sent to you by mail. You can return this consent form by using the attached return envelope. A postage stamp is not necessary.

Thank you for your time!

Sincerely,

Lieke van den Heuvel, MSc

Also on behalf of the research team and all the clinical geneticists and genetic counsellors involved from Amsterdam UMC, University Medical Centre Utrecht and University Medical Centre Groningen

Attachment: Information about your hospital 'UMCG/Amsterdam UMC/UMCU'

Questions, suggestions or complaints
If you have any questions, suggestions or a complaint about this research study, please contact Lieke van den Heuvel. She will conduct this research study. She can be contacted by telephone (<telephone number>) or by e-mail (<e-mail address>).

Independent advice or support
If you prefer to discuss study participation with someone who is not involved in this research study, because you need further information or advice, you may contact <independent researcher> (<e-mail address>). He/she is a clinical geneticist at the department of Clinical Genetics at UMCG/Amsterdam UMC/UMCU.

For peer review only

Supplementary Material S1

Certificate of consent
For participation in a research study regarding informing relatives at risk of an inherited cardiac disease

– I am satisfied about the information I received about this research study. I have had sufficient time to consider participation in this study. I have had the opportunity to ask questions about it and any questions that I have asked have been answered to my satisfaction.
– I can stop study participation at any time, without providing any reason and without any consequences.

By signing this form, I provide consent for:

1. Informing my clinician (clinical geneticist/genetic counselor) about my participation in this research study.
2. Collecting of data using questionnaire(s), and inspection of my medical record until 12 months after signing this consent form and the use of these data for the research study described in this letter.
3. Being approached for other research projects in the future.
4. Storing of my research data for 15 years after the research study has been finished
5. Inspection of the research data by the Safety Committee and the Dutch Health Care Inspectorate

I want to participate in this research study

Name of participant: ____

Date: ____/____/___

Signature of participant:____

I would like to be informed about the group results of this research study:

☐ Yes
☐ No

Statement by the researcher: I have accurately informed this person about the aforementioned research project. The person may stop participating at any time during the study without any consequences. Any questions from the person about study participation were answered sufficiently

Name of researcher:____

Date: ____/____/____

Signature of researcher: ____

For peer review only

Supplementary Material S1

Dear Sir or Madam,

Recently you visited the department of Clinical Genetics for genetic counselling concerning an inherited cardiac disease in your family. A clinical geneticist or genetic counsellor informed you about participation in a research study examining how to inform relatives at risk of inheriting the genetic predisposition. In this letter, we would like to inform you about this research study.

What is the goal of this research study?
At the current moment, we ask people in whom a genetic cause for a cardiac disease is identified to inform their relatives about the possibility of genetic testing, supported by a letter written by the clinician. This research study aims to investigate whether another approach to informing relatives would be more effective. Using this approach, people who are the first in their family in whom the hereditary predisposition is identified can decide which relatives they prefer to inform themselves and which relatives they prefer to have contacted directly by the clinician. In addition, a website will be used to further inform relatives. This research study will investigate which approach is the most effective.

Who is conducting the study?
This research study will be conducted by the Department of Clinical Genetics and the Department of Medical Psychology of the Amsterdam UMC, in collaboration with the Departments of Clinical Genetics of the University Medical Centre Groningen (UMCG) and the University Medical Centre Utrecht (UMCU). The study is funded by the Dutch Heart Foundation.

What does study participation involve?
For this research study, we ask you to fill out a questionnaire. This questionnaire can be administered online or by mail. If you prefer to receive the questionnaire by mail, the questionnaire can be returned by using the provided return envelope. These questionnaires ask you for some general information, such as your age and gender, and about your experiences with being informed about the inherited cardiac disease in your family and your opinion regarding the approach that was used. This questionnaire will also ask you some questions about how you feel, at the current moment and in general, and how you generally cope with complex situations.

In addition, it is important for this research study to receive some information about your medical background. Because of this, we ask you to provide consent to inspect your medical record up to 12 months after completion of this research study. Important information includes, for example, the inherited cardiac disease diagnosed in your family and information

For peer review only

Supplementary Material S1

about your family history regarding cardiac diseases. This information will be handled confidentially.

Your clinician (the clinical geneticist/genetic counsellor) will be informed if you decide to participate in this study. You might be approached for other research studies in the future.

What are advantages and disadvantages of study participation?
There will be no direct benefit to you for your participation in this study. We hope that the information obtained from this study will contribute to research on improving approaches to inform relatives at risk of inherited cardiac diseases. Participation in this research study will take time: we will ask you to fill out two questionnaires. We expect that this will cost you 20 to 30 minutes per questionnaire. In addition, we will ask questions regarding personal information, such as the disease in your family and your opinion and feelings concerning informing your relatives about this disease. You will not receive an incentive for study participation.

What if I do not want to participate anymore?
Your participation in this research is entirely voluntary. There are no consequences if you decide not to participate. You may also change your mind later and stop participating during the study even if you earlier agreed to do so. If you decide to stop participation, please let us know. The data collected up to that moment will be used for the research study. The researcher will inform you if there is new information about the study that might be important for you. In that case, you will be asked if you still consent to participate in the study.

How will my personal data be handled?
By participating in this study, you will provide us personal data. This data will be collected for the research study. A research code will be assigned to all research documents. This means that your name and other personal data are not visible on research documents. Only the researchers know which research code belongs to you. Some institutes (the Safety Committee and the Health Care Inspectorate) are allowed to look into your clinical and personal data. These institutes are allowed to do this to control whether the research study is conducted in a proper and reliable manner. They will treat your data confidentially. If you sign the informed consent form, you will provide consent for collection, storage and inspection of your medical and personal data. Your data will be stored for 15 years.

Do you have any further questions regarding this research study?
If you have any questions about this research study or about study participation, please contact the executing researcher Lieke van den Heuvel by telephone (<telephone number>) or e-mail (<e-mail address>). If you prefer to discuss study participation with an independent clinician, or if you would like additional information, advice or support, please contact the independent expert (see attachment).

What if I want to participate?
The researcher, Lieke van den Heuvel, will contact you by telephone to provide further information about the research study. If you decide to participate in this study after this telephone contact, a consent form will be sent to you by mail. You can return this consent

For peer review only

Supplementary Material S1

form using the attached return envelope. A postage stamp is not necessary. Subsequently, you will receive the questionnaire online or by mail.

Thank you for your time!

Sincerely,

Lieke van den Heuvel, MSc

Also on behalf of the research team and all the clinical geneticists and genetic counsellors involved from Amsterdam UMC, University Medical Centre Utrecht and University Medical Centre Groningen

Attachment: Information about your hospital ‘UMCG/Amsterdam UMC/UMCU’

Questions, suggestions or complaints
If you have any questions, suggestions or a complaint about this research study, please contact Lieke van den Heuvel. She will conduct this research study. She can be contacted by telephone (<telephone number>) or by e-mail (<e-mail address>).

Independent advice or support
If you prefer to discuss study participation with someone who is not involved in this research study, because you need further information or advice, you may contact <independent researcher> (<e-mail address>). He/she is a clinical geneticist at the department of Clinical Genetics at UMCG/Amsterdam UMC/UMCU.

For peer review only

Supplementary Material S1

Certificate of consent
For participation in a research study regarding informing relatives at risk of an inherited cardiac disease

– I am satisfied about the information I received about this research study. I have had sufficient time to consider participation in this study. I have had the opportunity to ask questions about it, and any questions that I have asked have been answered to my satisfaction.
– I can stop study participation at any time, without providing any reason and without any consequences.

By signing this form, I provide consent for:

6. Informing my clinician (clinical geneticist/genetic counselor) about my participation in this research study.
7. Collection of data using questionnaire(s), and inspection of my medical record until 12 months after signing this consent form and the use of these data for the research study described in this letter.
8. Being approached for other research projects in the future.
9. Storage of research data for 15 years after the research study has been finished
10. Inspection of the research data by the Safety Committee and the Dutch Health Care Inspectorate

I want to participate in this research study

Name of participant: ____

Date: ____/____/___

Signature of participant:____

I would like to be informed about the group results of this research study:

☐ Yes
☐ No

Statement by the researcher: I have accurately informed this person about the aforementioned research project. The person may stop participating at any time during the study without any consequences. Any questions from the person about study participation were answered sufficiently

Name of researcher:____

Date: ____/____/____

Signature of researcher: ____

For peer review only

Supplementary Material S2

Supplementary material S2: Overview of questionnaire items to assess secondary outcomes per time-point

Table 1 Questionnaire items probands per time-point

Questionnaire time-point	Items	Questionnaires
T1	Sociodemographic, clinical, family and personality factors	Self-constructed items<br>Trait scale of STAI<br>Shortened version of TMSI
	Advices for relatives at risk, eligible relatives at risk, the number of informed relatives at risk, risk perception and experiences with informing relatives at risk	Eleven self-constructed items
	Evaluation of used approach (incl. website)	Thirteen self-constructed items
	Perceived impact on family communication with relatives at risk	Adapted version of ODCF<br>One self-constructed item
	Impact on psychological functioning of proband	HADS<br><br>Adapted version of CWS
T2	Number of informed relatives at risk, risk perception and experiences with informing relatives at risk	Three self-constructed items
	Evaluation of approach used	One self-constructed item
	Perceived impact on family communication with relatives at risk	Adapted version of ODCF<br>One self-constructed item
	Impact on psychological functioning of proband	HADS<br>Adapted version of CWS

Table 2 Questionnaire items relatives

Questionnaire time-point	Items	Questionnaires
T1	Sociodemographic, family and personality factors	Eight self-constructed items<br>Trait scale of STAI<br>Shortened version of TMSI
	Evaluation of used approach (incl. website), risk perception	Thirteen self-constructed items
	Perceived impact on family communication with index patient	Adapted version of ODCF<br>One self-constructed item
	Impact on psychological functioning of family member	HADS<br>Adapted version of CWS

Table 3 Self-constructed items (telephone interview) - Experiences with informing relatives at risk (probands)

1. Did the genetic counsellor give you an advice for your relatives?<br>2. What was this advice? (open question)<br>3. For which relatives was this advice meant? (open question)	Yes/No

Supplementary Material S2

4. Have relatives at risk been informed about the advice of the genetic counsellor? If yes, which relatives have been informed?<br>5. Who informed your relatives?	Yes/No

Table 4 Self-constructed items (telephone interview) - Risk perception (probands)

1. How do you consider the risk of your relatives on being a carrier of the familial variant?	0% - 100%<br>1-10
2. How do you estimate the risk of your relatives on developing symptoms of the ICC?	0% - 100%<br>1-10

Table 5 Self-constructed items - Evaluation of the used approach (probands)

T1	*Closed questions*<br>*Below you can see statements regarding your experiences with how your relatives have been informed. Please rate each statement on a scale of 1-5 (1 totally disagree to 5 totally agree) how much each statement applies to you.*	
	1. I feel supported by the genetic counsellor in informing my relatives	1 2 3 4 5
	2. I think the used approach to inform relatives at risk is acceptable	1 2 3 4 5
	3. I felt a little coerced to inform my relatives	1 2 3 4 5
	4. The way my relatives are informed, can be improved	1 2 3 4 5
	5. I am satisfied with the way my relatives are informed	1 2 3 4 5
	Other:________________________________	
T2	1. Did your opinion regarding the used approach change?<br>a. Yes, my opinion regarding the used approach became more positive<br>b. Yes, my opinion regarding the used approach became more negative<br>c. No, my opinion regarding the used approach is still positive<br>d. No, my opinion regarding the used approach is still negative<br>e. No, my opinion regarding the used approach is still neutral	
T1/T2	1. Do you think another approach to inform relatives at risk would have been better?	Yes/No
	2. Are there relatives for which you would have preferred another approach to inform them?	Yes/No
T1/T2	*Open questions*<br>1. What are advantages of the approach used to inform your relatives?<br>2. What are disadvantages of the approach used to inform your relatives?	

Table 6 Self-constructed items - Impact on family relationships (probands)

1. Are there relatives with whom your relationship has changed after they are informed about their risk on the inherited cardiac disease?

For peer review only

Supplementary Material S2

a. Yes, our relationship improved<br>b. Yes, our relationship worsened<br>c. No, our relationship is still not good/not bad<br>d. No, our relationship is still good<br>e. No, our relationship is still bad

Table 7 Self-constructed items - Evaluation of the used approach (relatives)

Closed questions<br>1. How were you informed about the hereditary predisposition in your family?<br>a. With an information letter from the hospital, received from a relative<br>b. With a letter written by a relative<br>c. In person by a relative<br>d. With an information letter from the hospital, received from a genetic counsellor/clinical geneticist<br>e. In person by a genetic counsellor/clinical geneticist<br>2. What information did you receive (multiple answers are possible)?<br>a. The risk to be a carrier of the hereditary predisposition for the inherited cardiac disease in my family<br>b. The possibility to make an appointment for predictive DNA testing at an outpatient clinic Clinical Genetics<br>c. The advice to be regularly monitored by a cardiologist in the hospital<br>d. Something else, namely________________________________	
Below you can see statements regarding your experiences with how you have been informed about the inherited cardiac disease in your family. Please rate each statement on a scale of 1-5 (1 totally disagree to 5 totally agree) how much each statement applies to you.	
1. I appreciated to be informed about my risk on the inherited cardiac disease	1 2 3 4 5
2. I am satisfied with the way I have been informed	1 2 3 4 5
3. I preferred to have received more information before I contacted the clinical genetic centre	1 2 3 4 5
4. I understand why I have been informed	1 2 3 4 5
5. The way I have been informed, can be improved	1 2 3 4 5
6. I felt free to decide myself whether I wanted to contact the clinic genetic centre	1 2 3 4 5
7. I would have preferred not to be informed about my risk on the inherited cardiac disease in my family	1 2 3 4 5
8. I would have preferred to not know about the inherited cardiac disease in my family	1 2 3 4 5
Other:_________________________________	
1. Do you think another approach to be informed would have been better?	Yes/No
Open questions	

For peer review only - http://bmjopen.bmj.com/site/about/guidelines.xhtml

Supplementary Material S2

1. What are advantages of the way you have been informed?<br>2. What are disadvantages of the way you have been informed?	

Table 8 Self-constructed items - Impact on family relationships (relatives)

1. Did your relationship with your relative change after they were informed about their risk on the inherited cardiac disease?<br>a. Yes, our relationship improved<br>b. Yes, our relationship worsened<br>c. No, our relationship is still not good/not bad<br>d. No, our relationship is still good<br>e. No, our relationship is still bad

For peer review only

SPIRIT
STANDARD PROTOCOL ITEMS: RECOMMENDATIONS FOR INTERVENTIONAL TRIALS

SPIRIT 2013 Checklist: Recommended items to address in a clinical trial protocol and related documents*

Section/item	Item No	Description	Page no
**Administrative information**			
Title	1	Descriptive title identifying the study design, population, interventions, and, if applicable, trial acronym	1
Trial registration	2a	Trial identifier and registry name. If not yet registered, name of intended registry	2
	2b	All items from the World Health Organization Trial Registration Data Set	6-17
Protocol version	3	Date and version identifier	-
Funding	4	Sources and types of financial, material, and other support	18
Roles and responsibilities	5a	Names, affiliations, and roles of protocol contributors	1
	5b	Name and contact information for the trial sponsor	-
	5c	Role of study sponsor and funders, if any, in study design; collection, management, analysis, and interpretation of data; writing of the report; and the decision to submit the report for publication, including whether they will have ultimate authority over any of these activities	-
	5d	Composition, roles, and responsibilities of the coordinating centre, steering committee, endpoint adjudication committee, data management team, and other individuals or groups overseeing the trial, if applicable (see Item 21a for data monitoring committee)	-
**Introduction**			
Background and rationale	6a	Description of research question and justification for undertaking the trial, including summary of relevant studies (published and unpublished) examining benefits and harms for each intervention	4-6
	6b	Explanation for choice of comparators	4-6
Objectives	7	Specific objectives or hypotheses	6

For peer review only - http://bmjopen.bmj.com/site/about/guidelines.xhtml

Trial design	8	Description of trial design including type of trial (eg, parallel group, crossover, factorial, single group), allocation ratio, and framework (eg, superiority, equivalence, noninferiority, exploratory)	6

Methods: Participants, interventions, and outcomes

Study setting	9	Description of study settings (eg, community clinic, academic hospital) and list of countries where data will be collected. Reference to where list of study sites can be obtained	6
Eligibility criteria	10	Inclusion and exclusion criteria for participants. If applicable, eligibility criteria for study centres and individuals who will perform the interventions (eg, surgeons, psychotherapists)	7
Interventions	11a	Interventions for each group with sufficient detail to allow replication, including how and when they will be administered	9, 10
	11b	Criteria for discontinuing or modifying allocated interventions for a given trial participant (eg, drug dose change in response to harms, participant request, or improving/worsening disease)	17
	11c	Strategies to improve adherence to intervention protocols, and any procedures for monitoring adherence (eg, drug tablet return, laboratory tests)	-
	11d	Relevant concomitant care and interventions that are permitted or prohibited during the trial	-
Outcomes	12	Primary, secondary, and other outcomes, including the specific measurement variable (eg, systolic blood pressure), analysis metric (eg, change from baseline, final value, time to event), method of aggregation (eg, median, proportion), and time point for each outcome. Explanation of the clinical relevance of chosen efficacy and harm outcomes is strongly recommended	11-14
Participant timeline	13	Time schedule of enrolment, interventions (including any run-ins and washouts), assessments, and visits for participants. A schematic diagram is highly recommended (see Figure)	8, 11, 17
Sample size	14	Estimated number of participants needed to achieve study objectives and how it was determined, including clinical and statistical assumptions supporting any sample size calculations	15, 16
Recruitment	15	Strategies for achieving adequate participant enrolment to reach target sample size	-

Methods: Assignment of interventions (for controlled trials)

Allocation:

Sequence generation	16a	Method of generating the allocation sequence (eg, computer-generated random numbers), and list of any factors for stratification. To reduce predictability of a random sequence, details of any planned restriction (eg, blocking) should be provided in a separate document that is unavailable to those who enrol participants or assign interventions	9
Allocation concealment mechanism	16b	Mechanism of implementing the allocation sequence (eg, central telephone; sequentially numbered, opaque, sealed envelopes), describing any steps to conceal the sequence until interventions are assigned	9
Implementation	16c	Who will generate the allocation sequence, who will enrol participants, and who will assign participants to interventions	9
Blinding (masking)	17a	Who will be blinded after assignment to interventions (eg, trial participants, care providers, outcome assessors, data analysts), and how	9
	17b	If blinded, circumstances under which unblinding is permissible, and procedure for revealing a participant's allocated intervention during the trial	9
**Methods: Data collection, management, and analysis**			
Data collection methods	18a	Plans for assessment and collection of outcome, baseline, and other trial data, including any related processes to promote data quality (eg, duplicate measurements, training of assessors) and a description of study instruments (eg, questionnaires, laboratory tests) along with their reliability and validity, if known. Reference to where data collection forms can be found, if not in the protocol	11-15
	18b	Plans to promote participant retention and complete follow-up, including list of any outcome data to be collected for participants who discontinue or deviate from intervention protocols	-
Data management	19	Plans for data entry, coding, security, and storage, including any related processes to promote data quality (eg, double data entry; range checks for data values). Reference to where details of data management procedures can be found, if not in the protocol	17
Statistical methods	20a	Statistical methods for analysing primary and secondary outcomes. Reference to where other details of the statistical analysis plan can be found, if not in the protocol	16, 17
	20b	Methods for any additional analyses (eg, subgroup and adjusted analyses)	16, 17
	20c	Definition of analysis population relating to protocol non-adherence (eg, as randomised analysis), and any statistical methods to handle missing data (eg, multiple imputation)	16, 17

**Methods: Monitoring**			
Data monitoring	21a	Composition of data monitoring committee (DMC); summary of its role and reporting structure; statement of whether it is independent from the sponsor and competing interests; and reference to where further details about its charter can be found, if not in the protocol. Alternatively, an explanation of why a DMC is not needed	NA
	21b	Description of any interim analyses and stopping guidelines, including who will have access to these interim results and make the final decision to terminate the trial	17
Harms	22	Plans for collecting, assessing, reporting, and managing solicited and spontaneously reported adverse events and other unintended effects of trial interventions or trial conduct	-
Auditing	23	Frequency and procedures for auditing trial conduct, if any, and whether the process will be independent from investigators and the sponsor	NA
**Ethics and dissemination**			
Research ethics approval	24	Plans for seeking research ethics committee/institutional review board (REC/IRB) approval	17
Protocol amendments	25	Plans for communicating important protocol modifications (eg, changes to eligibility criteria, outcomes, analyses) to relevant parties (eg, investigators, REC/IRBs, trial participants, trial registries, journals, regulators)	-
Consent or assent	26a	Who will obtain informed consent or assent from potential trial participants or authorised surrogates, and how (see Item 32)	8, 9
	26b	Additional consent provisions for collection and use of participant data and biological specimens in ancillary studies, if applicable	NA
Confidentiality	27	How personal information about potential and enrolled participants will be collected, shared, and maintained in order to protect confidentiality before, during, and after the trial	17
Declaration of interests	28	Financial and other competing interests for principal investigators for the overall trial and each study site	18
Access to data	29	Statement of who will have access to the final trial dataset, and disclosure of contractual agreements that limit such access for investigators	17
Ancillary and post-trial care	30	Provisions, if any, for ancillary and post-trial care, and for compensation to those who suffer harm from trial participation	NA

For peer review only

Dissemination policy	31a	Plans for investigators and sponsor to communicate trial results to participants, healthcare professionals, the public, and other relevant groups (eg, via publication, reporting in results databases, or other data sharing arrangements), including any publication restrictions	17
	31b	Authorship eligibility guidelines and any intended use of professional writers	18
	31c	Plans, if any, for granting public access to the full protocol, participant-level dataset, and statistical code	-
**Appendices**			
Informed consent materials	32	Model consent form and other related documentation given to participants and authorised surrogates	-
Biological specimens	33	Plans for collection, laboratory evaluation, and storage of biological specimens for genetic or molecular analysis in the current trial and for future use in ancillary studies, if applicable	NA

*It is strongly recommended that this checklist be read in conjunction with the SPIRIT 2013 Explanation & Elaboration for important clarification on the items. Amendments to the protocol should be tracked and dated. The SPIRIT checklist is copyrighted by the SPIRIT Group under the Creative Commons "Attribution-NonCommercial-NoDerivs 3.0 Unported" license.

For peer review only
